# Validating Centralized Biobanking Workflows for NMR Metabolomics Using the PRIMA Panel

**DOI:** 10.1021/acs.analchem.4c04938

**Published:** 2025-01-28

**Authors:** Heidi Altmann, Marko Barovic, Katrin Straßburger, Maximilian Tschäpel, Sophie Jonas, David M. Poitz, Alexia Belavgeni, Triantafyllos Chavakis, Peter Mirtschink, Alexander M. Funk

**Affiliations:** † Medical Clinic & Polyclinic 1, University Hospital and Faculty of Medicine Carl Gustav Carus of TU Dresden, Dresden 01307, Germany; ‡ National Center for Tumor Diseases (NCT/UCC) Partner Site Dresden, Dresden 01307, Germany; § Institute for Clinical Chemistry and Laboratory Medicine, University Hospital and Faculty of Medicine Carl Gustav Carus of TU Dresden, Dresden 01307, Germany

## Abstract

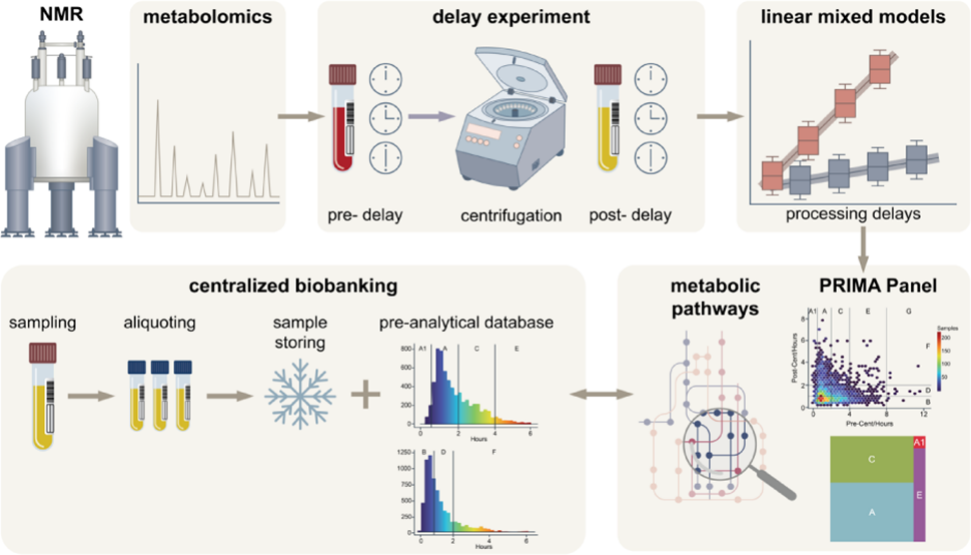

The quality of biological samples used in metabolomics research is significantly influenced by preanalytical factors, such as the timing of centrifugation and freezing. This study aimed to evaluate how preanalytical factors, like delays in centrifugation and freezing, affect metabolomics research. Blood samples, collected in various tube types, were subjected to controlled pre- and postcentrifugation delays. Metabolite levels were quantified using NMR spectroscopy and fitted in linear mixed models used to predict changes in metabolite concentrations over time. The results showed that some metabolites, such as lactic acid, were significantly affected by even short delays, while others remained stable for longer. The study introduced the concept of a “stability time point”, marking when a metabolite’s concentration changes by 20%. These predictive models were validated in a separate cohort. To apply these findings, the authors developed the PRIMA Panel, an open-source R Shiny tool. This tool allows researchers to assess the impact of preanalytical variations on their samples, predict metabolite stability, and generate performance reports. The PRIMA Panel was tested using samples from the Dresden Integrated Liquid Biobank, proving its utility in a real-world biobank setting. The study emphasizes the importance of tracking preanalytical factors to improve the reliability of metabolomics analyses. The PRIMA Panel is available online and for local deployment, providing a practical solution for quality control in metabolomics research. The results of the study underscore the importance of tracking preanalytical factors in biobanking. A versatile tool for assessing their impact on metabolic data is introduced, improving the reliability of future analyses.

## Introduction

Preanalytical factors can have an extensive impact on the quality of biological samples. The type of sample (e.g., blood or urine), the collection tube and anticoagulant, centrifugation, storage conditions and the time delay until processing and freezing can markedly alter the concentration of certain analytes. In fact, the time from collection to centrifugation (precentrifugation delay) and the time from centrifugation to measurement or storage (postcentrifugation delay) play a critical role in the variation observed in metabolic measurements.^1,2^ The rapid changes in metabolites during processing delays are often associated with cells still in contact with plasma or serum or enzymes that are still active.^3^ Another possible confounder, particularly in serum samples, is platelet (pre)activation and metabolism during clotting.^4^ It has been shown that platelets can use both glycolysis and fatty acid oxidation to support aggregation.^5^ Additionally, changes in amino acid levels have been associated with their abundance in proteins.^6,7^ Most recently, studies have shown linear relationships between precentrifugation delays and the concentration of a variety of metabolic parameters using NMR spectroscopy.^8,9^ However, cooling can drastically slow down these effects.^10^ Differences between serum and plasma have also been investigated extensively to identify the optimal collection tubes for metabolomics, yet the studies conducted so far have not yielded conclusive results.^11,12^ Cooling a peripheral blood sample can significantly impact the metabolome by slowing down metabolic alterations. However, it is important to distinguish between cooling before centrifugation and after centrifugation. Before centrifugation, it is not recommended to cool plasma samples as it may increase the risk of cell death and hemolysis.^13^ Serum is recommended to be at ambient temperature to facilitate clotting. Cooling after centrifugation can be incredibly helpful for samples that need to be temporarily stored or shipped before being frozen. Guidelines recommend processing a sample within 30 min and keeping it cool during the preanalytical process.^14^ Storing serum samples at 4 °C has been shown to be stable for up to 24 h.^15^ Freeze–thaw cycles are another factor that can significantly alter a sample’s physicochemical properties and should generally be avoided.^16,17^

In modern centralized biological repositories (biobanks), it is recommended to collect comprehensive preanalytical information for each submitted sample to effectively control for potential confounding factors and errors. This includes time-stamps for sample collection, centrifugation and freezing times.^17^ Such preanalytical information can be categorized using the SPREC (Standard PREanalytical Code), which is a seven-letter code that classifies the preanalytical quality of a sample.^18^ Each letter represents a different preanalytical condition, including sample material, type of tubes, as well as storage conditions. The pre- and postcentrifugation delays are categorized by letters 2 and 6, respectively. These letters categorize processing delays ranging from <30 min (A1) to >48 h (H) and indicate whether the delays occurred at room temperature (RT) or in a cooled state (2–10 °C). In a clinical setting or a centralized biobank the pre- and postcentrifugation delay can vary substantially from sample to sample.^19−21^

Measurements of metabolic parameters from such a collection can be problematic, due to the potential range of these processing delays. Here, we investigated the detailed effects of prolonged pre- and postcentrifugation times on metabolic parameters using NMR Spectroscopy with the objective of establishing a sample quality control tool for sample cohorts with varying processing delays. First, experiments were performed to observe the direct effect of prolonged pre- and postcentrifugation delays. These data were then used to generate linear mixed models that allow for prediction and quantification of the changes each metabolic parameter undergoes with prolonged processing delays. Finally, we aimed to validate samples obtained from a preanalytical workflow that contain variable pre- and postcentrifugation delays (e.g., centralized biobank) for NMR-based metabolomics. Here, we introduce the PRIMA Panel (**Pr**e-Analytical **I**nvestigator for NMR-based **M**et**a**bolomics). The PRIMA Panel, an open-source tool, was created to allow researchers to directly assess the preanalytical information on their sample cohorts and observe the effects of preanalytic variability on metabolic parameters. Bruker BioSpin offers a commercially available tool termed BiobankQC, for plasma/serum and urine samples analyzed using NMR spectroscopy and their IVDr methods. This panel provides an overview of proper sample preparation, potential contamination, and the accuracy of NMR measurements. Additionally, it detects the anticoagulant used in collection tubes (e.g., citrate) and evaluates matrix integrity parameters (e.g., lactate, glucose), which reflect preanalytical variations such as bacterial growth or improper temperature handling during preanalytical procedures. The panel also assesses changes in the concentration and chemical shift of rapidly varying metabolites like glucose and lactate caused by prolonged preanalytical delays. However, it only delivers a basic pass/fail output for these parameters. PRIMA was developed to enhance this functionality, offering a broader range of information on processing delays, and a user-friendly overview tailored for biobank users.

A cohort from the Biobank Dresden (BBD) was used to highlight a realistic range of preanalytical variations and provide an example workflow for the PRIMA Panel. The PRIMA Panel could prove useful in cases where samples were collected in a unique clinical situation with no possibility of repeating sample collection.

## Experimental Section

### Sample Collection

The study was approved by the ethics committee of the TU Dresden. Blood samples were obtained from 30 healthy male and female donors who provided written informed consent. Blood was collected (precentrifugation *n* = 30, postcentrifugation *n* = 27) in different collection tubes: ethylenediaminetetraacetic acid plasma (EDTA), lithium-heparin plasma (LiHep) and a tube with coagulation activator for collection of serum. The samples were left at RT for different periods of time (0, 2, 4, 6, and 8 h) before centrifugation or after centrifugation in separate experiments (Figure S1). The time of blood drawing was used as the data point for time 0 h; for serum the time 0 was 20 min after sampling to allow clotting. All collection tubes were centrifuged at 2,000 × *g* for 10 min at RT. The cell count was measured using a SYSMEX XN 9000 immediately after blood draw. After the time delays, the centrifuged samples were processed using a HAMILTON STARlet pipetting robot to generate aliquots of plasma/serum, which were immediately frozen and stored at −80 °C. For NMR spectroscopy, the samples were thawed at RT for 30 min.

### Case Study Cohort BBD

The preanalytical data of BBD samples were gathered from the CentraXX database (Version 3.17.0.7) for all EDTA and serum samples collected between 01-01-2020 and 31-12-2023, totaling 7410 samples. The timestamps for sampling, centrifugation and freezing were used to calculate exact delays for pre- and postcentrifugation times. Samples with either missing values of extreme processing times (longer than 6 h) were removed, leaving 6312 samples for analysis. For the final analysis using the PRIMA Panel, the cohort was reduced to serum samples collected between 2021 and 2022 (756 samples) to obtain a realistic sample size for a study cohort.

### NMR Spectroscopy

Sample preparation and ^1^H NMR spectroscopy measurements were performed according to established protocols^14,16^ and following the Bruker IVDr Methods protocols. All NMR measurements were carried out on a Bruker BioSpin 600 MHz AVANCE NEO equipped with a BBI-Probe (5 mm) and a Bruker SampleJet robot with a cooling system for sample storage at 4 °C. A full quantitative calibration was performed before each measurement. The panels used for the metabolic profiles were B.I.Quant-PS (version 2.0.0) and B.I.LISA (version 1.0.0), which include up to 42 metabolic parameters and 112 lipid subclass parameters. The names of the B.I.LISA parameters were changed for clarification (Table S1). The BiobankQC-PS (version 1.0.0) was also measured.

### Analysis and Programming

Metabolites with more than 30% missing values were removed from the analysis. Statistical analysis and model creation were performed in R utilizing the packages “rstatix”, “lme4” and “performance”. Models were created with about 2/3 of the measured samples (*n* = 20), and the remaining samples were used for the validation cohort (*n* = 10 for precentrifugation, *n* = 7 for postcentrifugation). The linear mixed models were created with cell count as a random slope and donors as random intercepts (Tables S4 and S5). The validation cohort was used to calculate the percentage mean error for the difference between predicted values from the models and actual values of the validation cohort. The PRIMA Panel application was written in the R environment^22^ and utilizes the package shiny^23^ to display it as a web application. A web version of the application is permanently hosted on the shinyapps.io platform via https://funkam.shinyapps.io/PRIMA/. The source code, as well as the original data tables, are available on GitHub via https://github.com/funkam/PRIMA and available for local deployment.

## Result and Discussion

### Case Report Dresden Integrated Liquid Biobank (DILB) Cohort

Pre- and postcentrifugation times are a common challenge in centralized biobanks. Depending on the biobank structure and workflows, multiple hours can pass before samples are processed or frozen. The Dresden Integrated Liquid Biobank (DILB), as part of the Biobank Dresden (BBD) is integrated into the clinical diagnostic workflow of the centralized clinical laboratory at the University Hospital Dresden. In this approach, samples are not collected at the site of processing, which may lead to prolonged pre- and postcentrifugation delays lasting up to multiple hours. Full preanalytical information is automatically tracked and stored in a database along with aliquoting information. Full SPREC information is calculated from time stamps.

**Figure 1:**
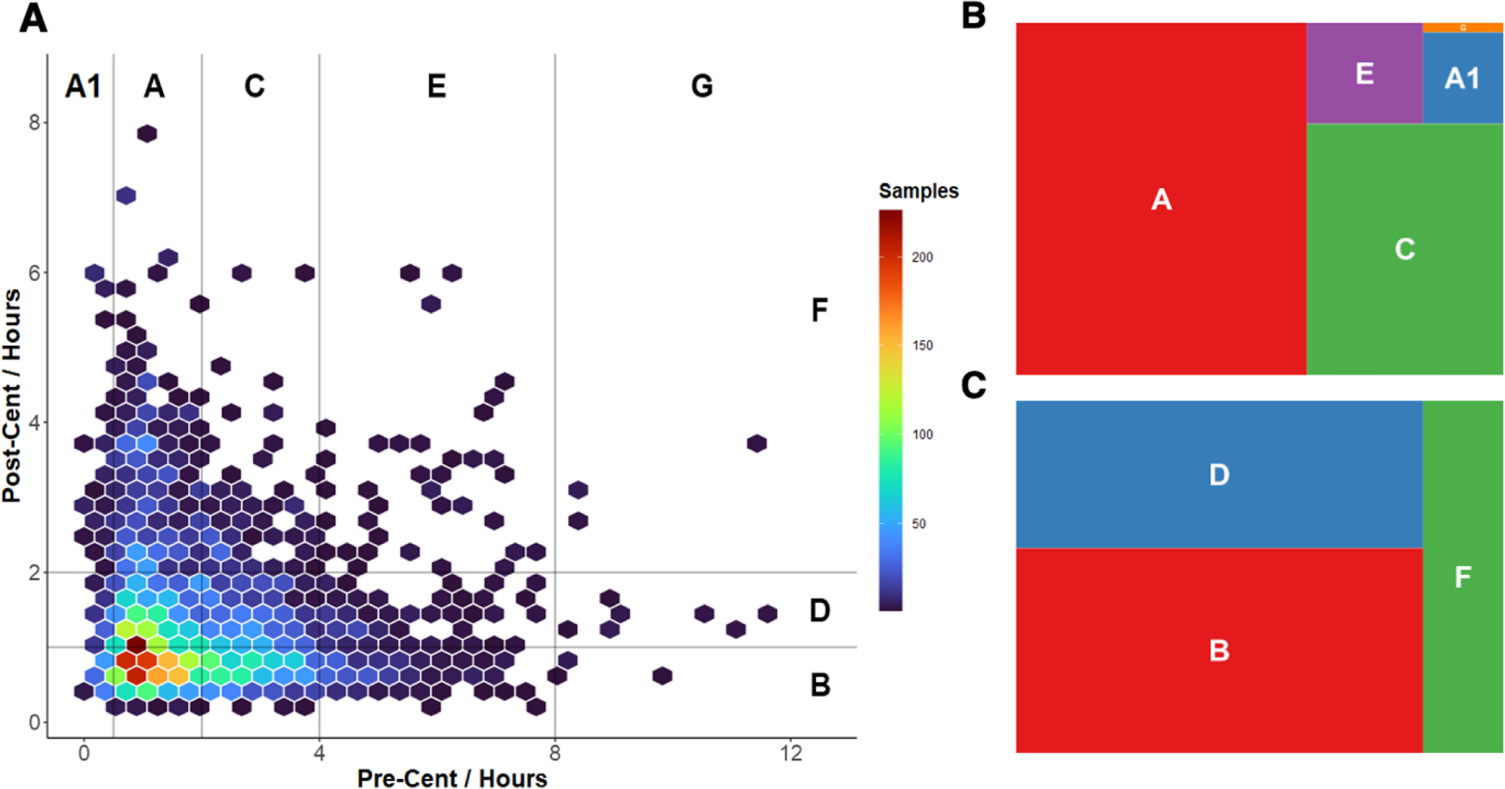
Preanalytical plots of the DILB cohort with (A) 2D hex-histogram with categorization according to SPREC (precentrifugation delay letters on top (A1 < 30 min, A < 2 h, C 2–4 h, E 4–8 h, G 8–12 h, postcentrifugation delay letters on the right (B < 1 h, D 1–2 h, F 2–8 h)), (B) distribution of SPREC category for precentrifugation delays (the small letter is category G), and (C) distribution of SPREC category for postcentrifugation delays.

To obtain an overview of the common delays experienced by the DILB collection, the preanalytical information on all samples (EDTA and serum) that were collected between 2020 and 2023 was analyzed (Table S2). The 2D hex density plot in Figure 1A illustrates the pre- and postcentrifugation time distribution in our cohort. A wide range of processing delays ranging up to 12 h in precentrifugation, and up to 8 h in postcentrifugation could be observed. Although the majority of samples were centrifuged within 2 h and could therefore be assigned to the SPREC category A and A1 (Figure 1B, 59.7 and 4.3% respectively), there is still a large number of samples in category C (2–4 h) (28.9%) and E (4–8 h) (6.8%). For the postcentrifugation delay, close to a majority of samples were in category B (<1 h) (48.6%) and D (1–2 h) (34.9%) (Figure 1B). Similarly, a subset of samples had prolonged postcentrifugation times placing them in category F (16.5%) (2–8 h).

The range of preanalytic variation observed here highlights the importance of tracking preanalytic information. For metabolic analyses, samples in the lower categories (A1, A in precentrifugation and B, D in postcentrifugation) could be used directly for downstream analysis. However, limiting the analysis only to these categories would have a severe impact on the final number of available samples. While the effect of prolonged precentrifugation delays has been investigated previously, the resources needed to facilitate quality assurance filtering based on this parameter are limited.^2,8^ To the best of our knowledge, the impact of postcentrifugation delays had not been investigated until now.

With the goal to comprehensively study the effect of pre- and postcentrifugation delays on a large number of metabolic parameters simultaneously, a series of samples was collected with controlled variation of pre- and postcentrifugation delay.

### Modeling Processing Delays

Two experiments were performed to investigate the direct effect of processing delays on metabolic parameters (Figure S1). First, a series of samples were left at RT for 2 h intervals (up to 8 h) before centrifugation and plasma/serum were then frozen. Second, another series of samples were centrifuged immediately after collection and then left at RT for 2 h intervals (up to 8 h). Each experiment was done to simulate the collection of plasma (with EDTA or LiHep as anticoagulants) and serum. Subsequently, plasma/serum metabolomic profiles were measured by NMR spectroscopy. In addition, a complete blood cell count was done for each donor, probing for potential effects of cell number variation on the rate of change of individual metabolic parameters (Table S3).

To investigate the rate of change of metabolic parameters due to processing delays linear mixed models were created analogous to previous studies.^8^ Linear mixed models allowed the flexibility needed for the range of values observed across the donors (e.g., alanine in Figure S3). This was achieved by assuming a different intercept for each donor for each parameter. To account for the potential influence of blood cells present in the sample on metabolite concentrations, additional models were created that also included the total cell number. Unexpectedly, the cellular component as a random factor had only a very limited influence on the models, however the models using the cellular component had more favorable estimated r^2^ value (Table S4). Energy metabolites (lactate, glucose, citrate) showed no differences between the models (Table S5). However, all measured cell counts in this study were within the physiological range (Table S3), therefore it is not possible to generalize these results also to samples with a pathological cell count.

The linear relationship between processing delays and the measured parameters allowed for the creation of a metric that directly reflects the extent of change each parameter undergoes with prolonged processing delays. This metric termed “the stability time point” was defined as the time it takes for a specific parameter to change by 20% compared to its initial value at no processing delay. For example, lactic acid had a precentrifugation stability time point of 0.41 h in EDTA, which means it took 0.41 h (24.6 min) for the concentration value of lactic acid to change by 20%. The shorter the stability time point, the stronger the alteration of a metabolic parameter is due to processing delays. The previously calculated linear mixed models were used to estimate the stability time point for each parameter and for both processing delays.

### Quantifying Stability before Centrifugation

The type of sample (plasma/serum) had a systematic effect on the concentrations of metabolic parameters. While the differences between the two types of plasma (EDTA, LiHep) were minimal, the differences in concentrations compared to serum were more apparent (Figure S2). These differences have been reported before and were confirmed here,^9,11^ emphasizing the importance of consistent material choice in metabolomics studies. The difference between plasma and serum was also found in the stability time points (Table S6).

**Figure 2:**
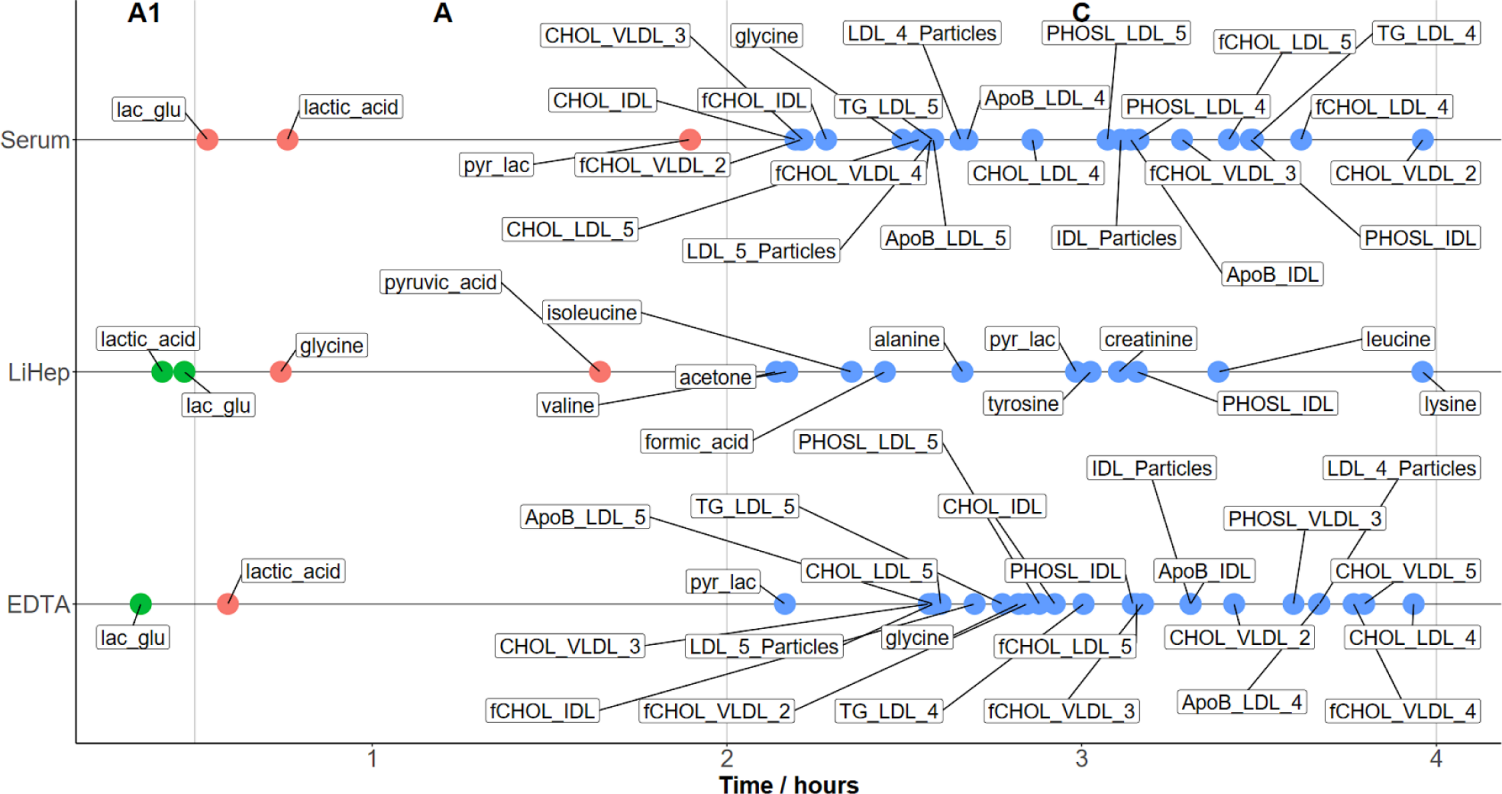
Timeline plots (to 20% change per parameter) for precentrifugation delays, letters are classification according to SPREC (A1 <30 min, with parameters labeled in green; A <2 h, with parameters labeled in red and C 2–4 h), with parameters labeled in blue. Full data available in Table S6.

In the precentrifugation experiment, the stability time points spanned a time frame from 21 min up to 48 h (Figure 2). The parameters with the shortest stability time points) were lactic acid (24 to 45 min) and the ratio of lactic acid to glucose (21 to 32 min). This finding aligns with previous studies using these parameters as markers for preanalytic quality.^24^ While glutamic acid has been described alongside lactic acid as a marker of sample quality, it had to be removed from this analysis due to a large number of missing values and concentration levels close to the limit of detection.^8^ Most of the included parameters remained largely stable throughout the first 2 h (SPREC category A), except for a few metabolites related to energy metabolism in LiHep plasma (e.g., glycine, pyruvate). Between 2 and 4 h (SPREC category C), a notable number of amino acids were observed in LiHep plasma. However, for serum and EDTA plasma mostly lipid parameters are found in this category. Overall there were 26 parameters with the precentrifugation stability time point within the first 4 h for EDTA plasma and serum and 15 for LiHep (Table S6). When choosing between these collection tubes and under the expectation of a precentrifugation delay, the timeline shown in Figure 2 could assist with the decision depending on the parameters of interest. Recent studies with similar methodology investigated the stability of metabolites during the precentrifugation period,^8,9^ but only the percentages of changes were reported. Similar to the results presented here, the parameters most affected by the precentrifugation time showed similar dynamics and the lipid parameters were more stable than nonlipid parameters. Overall, it seems that for samples centrifuged within the first 2 h, serum and EDTA plasma are the better choice. A similar conclusion was reached in previous studies, and in SOPs suggested by the Bruker IVDr methods.^11,12^ Lactic acid measurements would most likely have to be discarded with any precentrifugation delay, unless fluoride-supplemented EDTA tubes have been used to actively inhibit glycolysis.

### Quantifying Stability after Centrifugation

The stability time points were also estimated for postcentrifugation (Figure S4). As expected, centrifugation stopped most of the processes that altered metabolite concentrations by separating the cellular component. However, similar to the precentrifugation delays, lactic acid had a short stability time point of 0.81 h (48.6 min) in EDTA plasma and 1.63 h (97.8 min) in LiHep plasma. In serum, lactic acid is remarkably stable (4.3 h). Except for lactic acid, the lactic acid/glucose ratio, pyruvate and glycine, the levels of measured parameters in EDTA and LiHep plasma were notably stable for the first 2 h. In serum, however, concentrations of several lipid parameters (e.g., IDL and VLDL of CHOL and fCHOL) markedly changed during a 2-h postcentrifugation delay. The overall number of stability time points within the first 4 h in serum and EDTA plasma was similar to those in the precentrifugation experiment (23 in serum and 26 in EDTA plasma). Notably, in the postcentrifugation delay, LiHep plasma had only 8 parameters with stability time points within the first 4 h. However, adhering to the SPREC classification for postcentrifugation delays, category F extends from 2 to 8 h, which increases the number of stable parameters to around 60 in serum and EDTA plasma and 45 for LiHep plasma (Table S6). While the SPREC classification adequately categorized metabolites for precentrifugation delays, the results of this study suggest a need to redefine the large time span in category F. Overall, both LiHep and EDTA plasma showed changes only in lactic acid and the lactic acid/glucose ratio during the first 2 h (with additional changes in pyruvate and glycine observed in LiHep plasma only) and appear to be better choices for handling prolonged postcentrifugation delays. Furthermore, LiHep had fewer altered parameters even between the 2–4-h mark. Finally, when prolonged pre- and postcentrifugation delays are expected, the decision on the material to be collected should consider the study aims and parameters of interest.

### Validation

**Figure 3:**
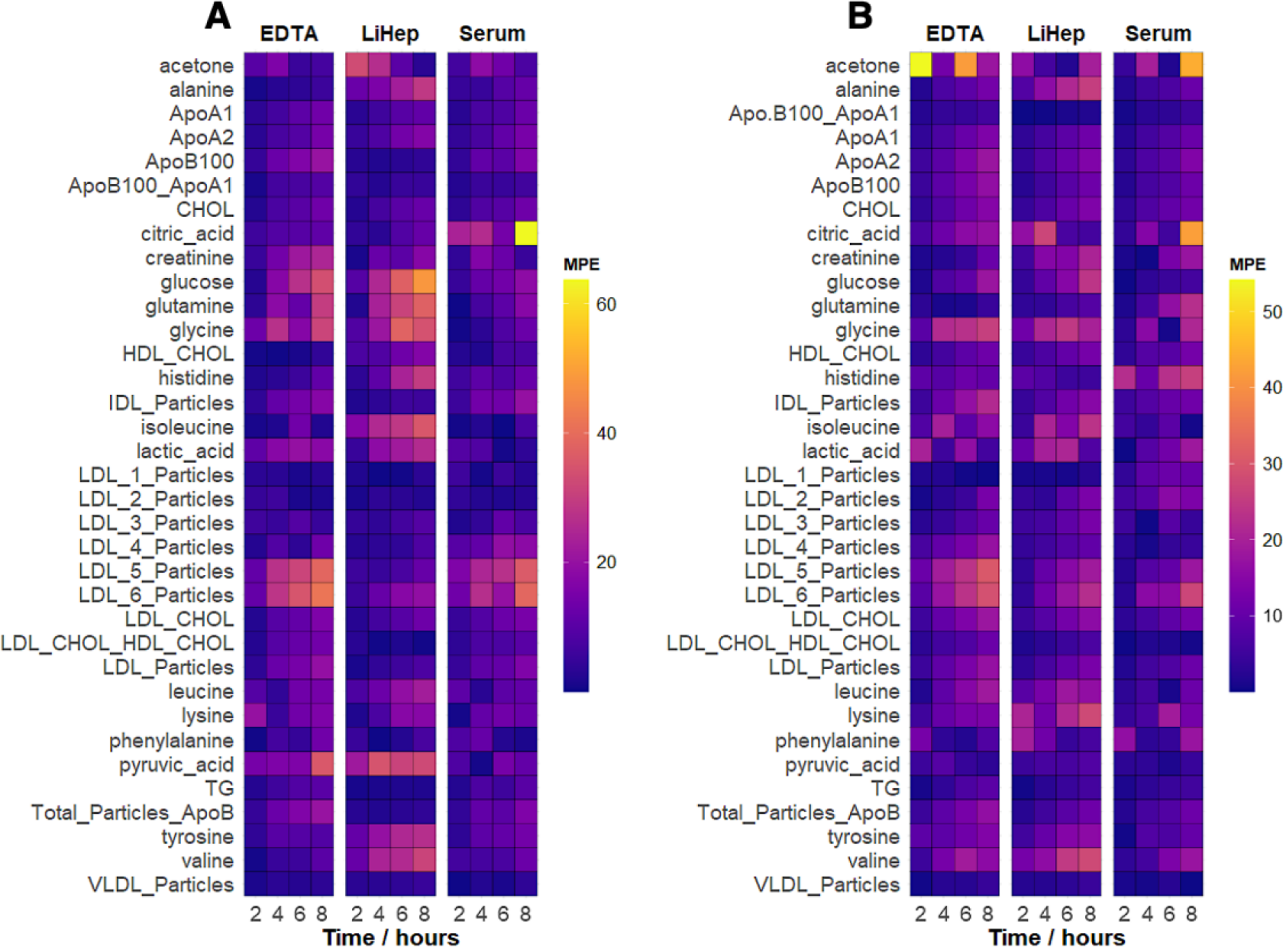
Heatmap overview of the estimates mean percentage error (MPE) between the (A) precentrifugation and (B) postcentrifugation models and the measured values of the validation cohort. Absolute values used. Not all parameters shown for reasons of clarity. Full data in Table S7.

A validation cohort was collected and measured in the same way as the cohort used for setting up the linear mixed models. The initial data point (time = 0) was used together with the linear mixed models to predict the values of the remaining time points (2, 4, 6, 8 h). The difference between the measured values at these later time points and the predicted values was estimated using the mean percentage error (MPE). This analysis was performed for both pre- and postcentrifugation delays (Figure 3, Table S7). The models offered a robust prediction of most parameter levels, especially for the 2- and 4-h time points, where the MPE remained well below 10% with the exception of postcentrifugation acetone in EDTA plasma.

Nevertheless, at later time points and for specific parameters, the models did not perform satisfactorily (MPE < 30%) for all parameters, with varying performance for pre- and postcentrifugation predictions. To understand these deviations from the models it is necessary to consider the metabolite’s concentration level, the rate of change it undergoes during the time-interval, and the estimated r^2^ of its linear mixed models (Table S4). Parameters measured close to the limit of detection are naturally associated with higher error and, consequently, greater variability in the model and the resulting MPE (e.g., acetone in EDTA, postcentrifugation). Similarly, a smaller gradient in the model lead to minimal changes during processing delays, and the inherent measurement error may result in higher variability in the validation cohort (e.g., glutamine in LiHep, precentrifugation). Additionally, the model itself, as quantified by r^2^ might poorly predict the metabolite’s response during processing delays. For example, glucose is measured at high concentrations and shows the second-highest rate of change among the metabolites. However, the MPE for glucose in EDTA and LiHep is unexpectedly large at later time points. This discrepancy likely arises because the concentration does not follow the linear response dictated by the linear mixed models (see r^2^ in Table S4), leading to poor predictions at later time points. Other factors, such as long-term storage, freeze–thaw cycles, or cooling, could also influence the metabolic profile in ways not accounted for by these models. However, for room temperature processing delays, the current models effectively predict changes in concentrations within the range relevant for the majority of samples processed in centralized biobanks, as demonstrated by the example cohort in Figure 1.

### Pre-analytical Investigator for NMR-Based Metabolomics (PRIMA Panel)

Furthermore, an R Shiny web application was created using the previously presented linear mixed models. This application enables users to assess the effects of preanalytic variation on their data and generate performance reports for their sample collection. The tool is hosted online permanently but can also be run locally using the code deposited in GitHub.

The panel is split into two different parts. First, in the “Data” module, the data of the preanalytical variation experiments, the linear mixed models and the stability time points are presented in interactive table and plots. Here, users can set their own threshold (in percent) for the stability time points and the metabolic parameters are automatically categorized into SPREC and sorted according to their time points as a means to explore the data in more detail. The timeline plots analogous to those in Figures 2 and S4 are also reproduced. This part of the panel is designed as a quick reference guide and as an extension of the data presented here.

**Figure 4:**
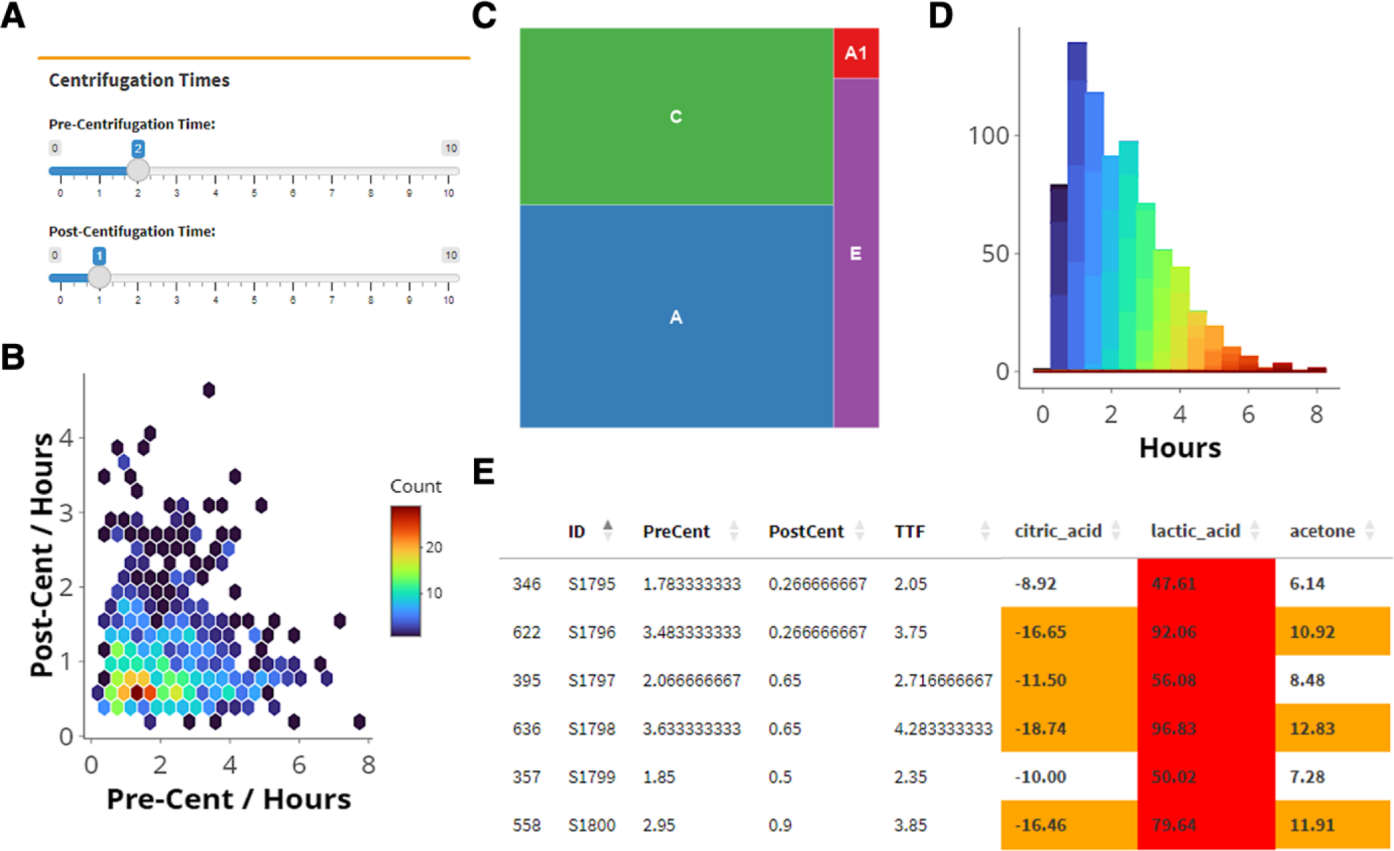
“Performance” module of the PRIMA Panel. (A) Snapshot of the user interface, and presentation of the analytical plots for the reduced DILB cohort of just serum samples (B–E). Histogram, density plot, and SPREC distribution are produced automatically.

The “Performance” module of the PRIMA Panel provides a more granular view of the concentration changes of the measured analytes, as well as an overview of the preanalytical information on a sample collection. Users can enter the data for a single sample and explore the effects using an interactive slider (Figure 4A) or upload a file with preanalytical information on a sample collection. A tool is available to prepare a table in the format required for the reports, which automatically calculates pre- and postcentrifugation delays from date-time stamps. To calculate the postcentrifugation delay accurately, it is also possible to set the centrifugation time in case the time stamps were obtained from the beginning of the centrifugation. The tool also generates performance reports containing the SPREC classification and a variety of visualizations that provide an overview of the preanalytic characteristics of the cohort (Figure 4B–D).

The final part of the PRIMA Panel involves predicting levels of metabolic parameters. Here, in vitro changes in concentrations of metabolic parameters are estimated using a combination of two different linear mixed models for pre- and postcentrifugation delays. The prediction results are presented as a dynamic table that users can customize for colors and thresholds (Figure 4E). The MPE, as mentioned above is included as an interactive input variable and parameters can be filtered using the “Tolerable Error” panel. In summary, users upload a table with datetime-stamps for collection, centrifugation and freezing and the tool creates automated analytical plots, as well as provides information about the metabolic changes expected for each sample. The output can be saved as an interactive HTML document. The PRIMA Panel is permanently hosted online (https://funkam.shinyapps.io/PRIMA/). The source code is openly available and can be deployed locally.

### PRIMA Panel Use Case on the DILB Cohort

To demonstrate the use of the PRIMA Panel, 765 serum samples collected between 2021 and 2022 within the DILB cohort were used. A tolerable error of 15% was applied in the metabolite predictions. The pre- and postcentrifugation delays reached up to 8 and 4 h, respectively. The distributions of pre- and postcentrifugation times as well as SPREC classifications were generated (Figure 4B–D). The final example report is included in the supplementary files. The data set can be further filtered according to the estimated percentage of concentration change of the parameters or the SPREC category of the sample. To aid in the filtering step, a table with predicted changes in the concentration of individual parameters is also provided (Figure 4E). In this demonstration, a large majority of samples fall into the SPREC categories A1, A and C. While this is generally considered acceptable, the detailed table highlights the potential drastic alterations in concentrations of specific metabolites. Taking into consideration the information provided by the 2D hex density plots, 1D histograms (Figure 4B,D, respectively); the metabolites table, and importantly, the aims of the study, tailored cutoff values for pre- and postcentrifugation times can be defined. This allows the analyst to achieve an individual balance between cohort size and integrated sample quality as assessed by the PRIMA Panel.

To aid in tuning the filtering options, the “Single Sample” performance report option can be used. In the example presented here, most metabolites were altered by around 5%, apart from glycine and lactate. This option is particularly useful when considering the postcentrifugation changes, as filtering according to SPREC categories might be too blunt due to the category F spanning 2 to 8 h, making a more granular cutoff more appropriate.

Overall, the PRIMA Panel can provide guidance and information on how to make educated decisions on filtering the samples and supports the idea of relative filtering instead of using a straight cutoff.

## Conclusion

In this study, a combination of pre- and postcentrifugation delays was modeled to obtain a comprehensive overview of preanalytical process for metabolomics samples in the setting of a centralized biobank. The models were used to identify metabolic parameters that change due to these processing delays. A validation cohort was used to investigate the robustness of these models. Next, these models and data were combined to create the PRIMA Panel, which allows the exploration of the data and provides output in form of performance reports. The data presented here highlight the importance of tracking preanalytical information for biobanking repositories. Additionally, it provides a flexible tool to investigate the direct effect of preanalytic delays on metabolic parameters facilitating data driven filtering of samples and parameters to increase the reliability of subsequent analyses.

## Data Availability

The raw data and models created from it are available on the GitHub page: https://github.com/funkam/PRIMA. The software is also permanently hosted at https://funkam.shinyapps.io/PRIMA.

## Supporting Information Available

Study outline, box plots for metabolic measurements, full table for the stability time points, SPREC cohort distribution, blood count, and full model information (PDF)
