# Supplementary material for: Validating Centralized Biobanking Workflows for NMR Metabolomics Using the PRIMA Panel
Source: Anal Chem. 2025 Jan 28;97(5):2762–9. doi: 10.1021/acs.analchem.4c04938 (PMC11822734; doi:10.1021/acs.analchem.4c04938)
Supplement: Supplementary file 1 — ac4c04938_si_001.pdf [file ac4c04938_si_001.pdf]

Validating centralized biobanking workflows for NMR Metabolomics using the PRIMA-Panel

Heidi Altmann[1,2#], Marko Barovic[3#], Katrin Straßburger[3], Maximilian Tschäpel[1], Sophie Jonas[3], David Poitz[3], Alexia Belavgeni[3], Triantafyllos Chavakis[2,3], Peter Mirtschink[2,3,&,*], Alexander M. Funk[2,3,&,*]

Author Information:

[1]Medical Clinic & Policlinic 1, University Hospital and Faculty of Medicine Carl Gustav Carus of TU Dresden, 01307 Dresden, Germany

[2]National Center for Tumor Diseases (NCT/UCC) Partner Site Dresden, 01307 Dresden, Germany

[3]Institute for Clinical Chemistry and Laboratory Medicine, University Hospital and Faculty of Medicine Carl Gustav Carus of TU Dresden, 01307 Dresden, Germany

[#]equal first authorship

[&]equal last authorship

*corresponding authors:

alexander.funk@ukdd.de; ORCID-ID: 0000-0002-7248-4599

peter.mirtschink@ukdd.de; ORCID-ID: 0000-0001-5832-1673

Table of Contents

Figure S1: Study Outline of the pre- and post-centrifugation experiments S2
Figure S2: Overview of the distribution of metabolic parameters in the pre-centrifugation experiment for EDTA (red), LiHep (orange) and Serum (yellow) at time point 0, 2 ,4 6, and 8 hours. X-Axis omitted for clarity........ S3
Figure S3: Overview of alanine fitting in serum for each donor. The blue line representing standard linear models and the green the linear mixed models with intercept set as a random effect............ S7
Figure S4: Timeline plots showing the time to 20% change per parameter for post-centrifugation delay. Letters are correspond to the SPREC classification (B <1h, D <2h, F 2-8h). S8

Table S1: Differences in the naming of lipid parameters between official Bruker names and the names used in this study. S9
Table S2: Overview of samples used in the DILB cohort (EDTA and serum samples combined). Pre-centrifugation corresponds to SPREC letter 2 and post-centrifugation corresponds to SPREC letter 6. .... S10
Table S3: Blood cell count for each donor from the model cohort in GPt/L. S11
Table S4: Model information with and without cells as a random factor for the linear mixed models of the pre-centrifugation experiment. S12
Table S5: Model Information for the linear mixed models of the post-centrifugation experiment... S19
Table S6:Overview of metabolic parameters that change significantly (>20%) in the times categorized by the SPREC classification. Time-point were calculated from linear-mixed models in hours for the pre-centrifugation (left) and post-centrifugation (right) experiments. S26
Table S7: Estimated mean percentage error values per time point for every parameter for pre-centrifugation and for post-centrifugation models. S33

Figure S1: Study Outline of the pre- and post-centrifugation experiments.

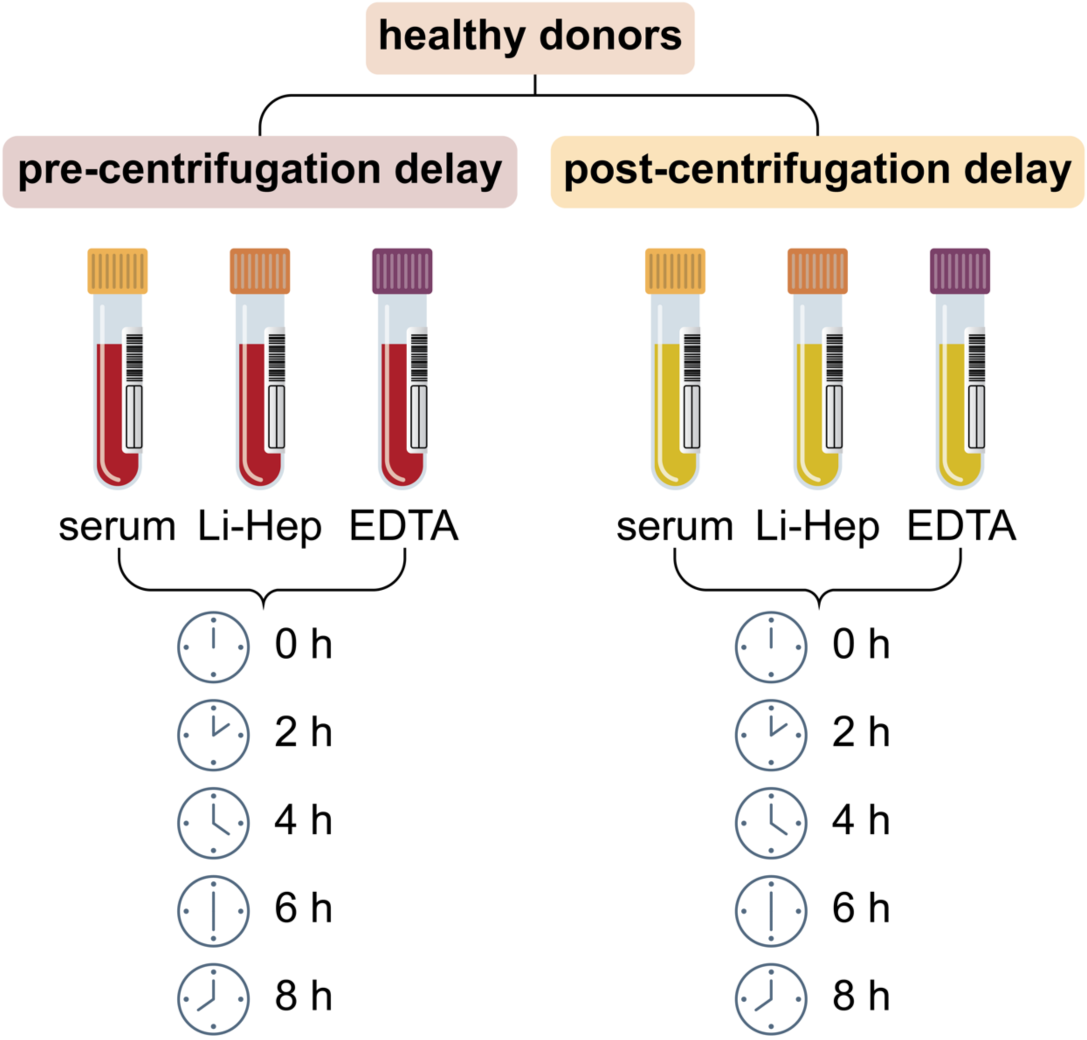

Figure S2: Overview of the distribution of metabolic parameters in the pre-centrifugation experiment for EDTA (red), LiHep (orange) and Serum (yellow) at time point 0, 2 ,4 6, and 8 hours. X-Axis omitted for clarity.

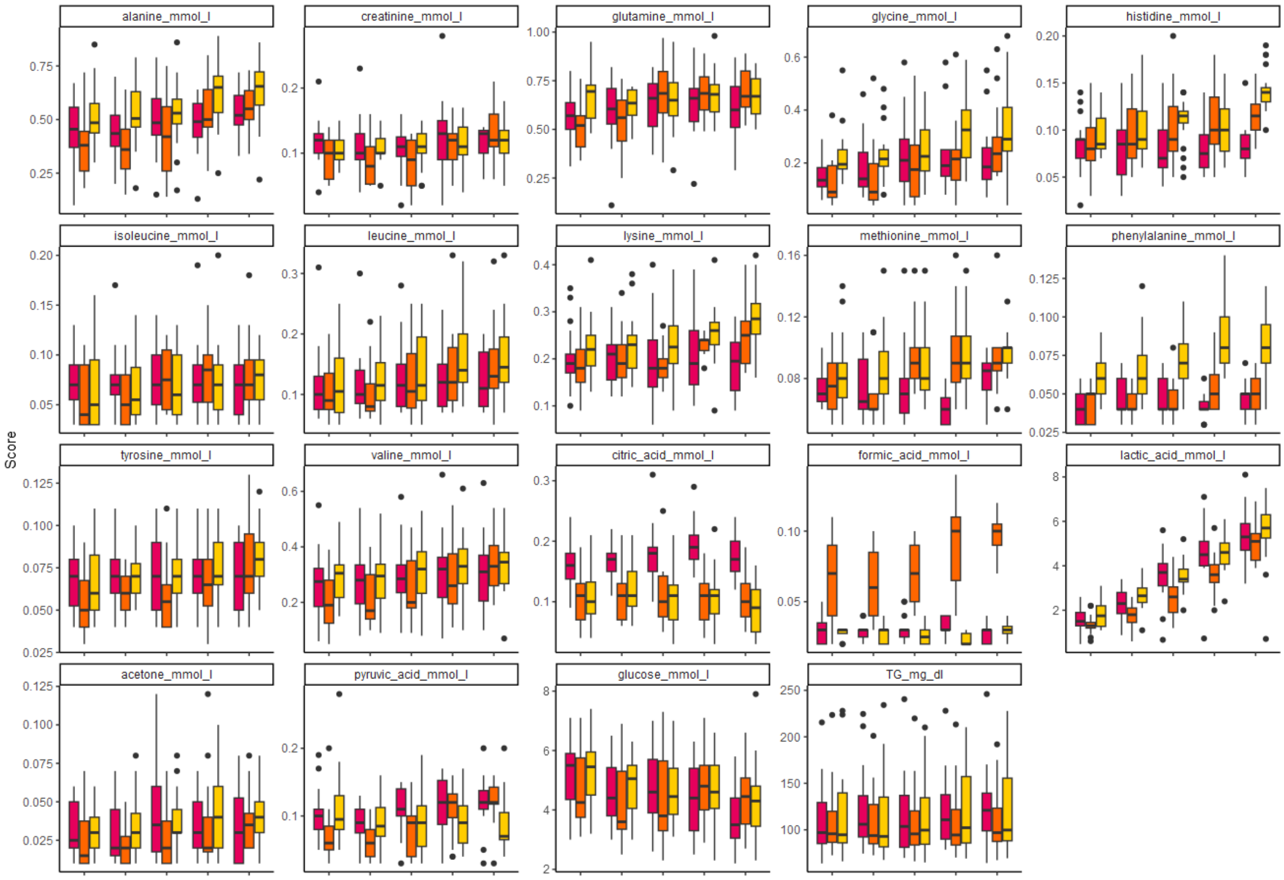

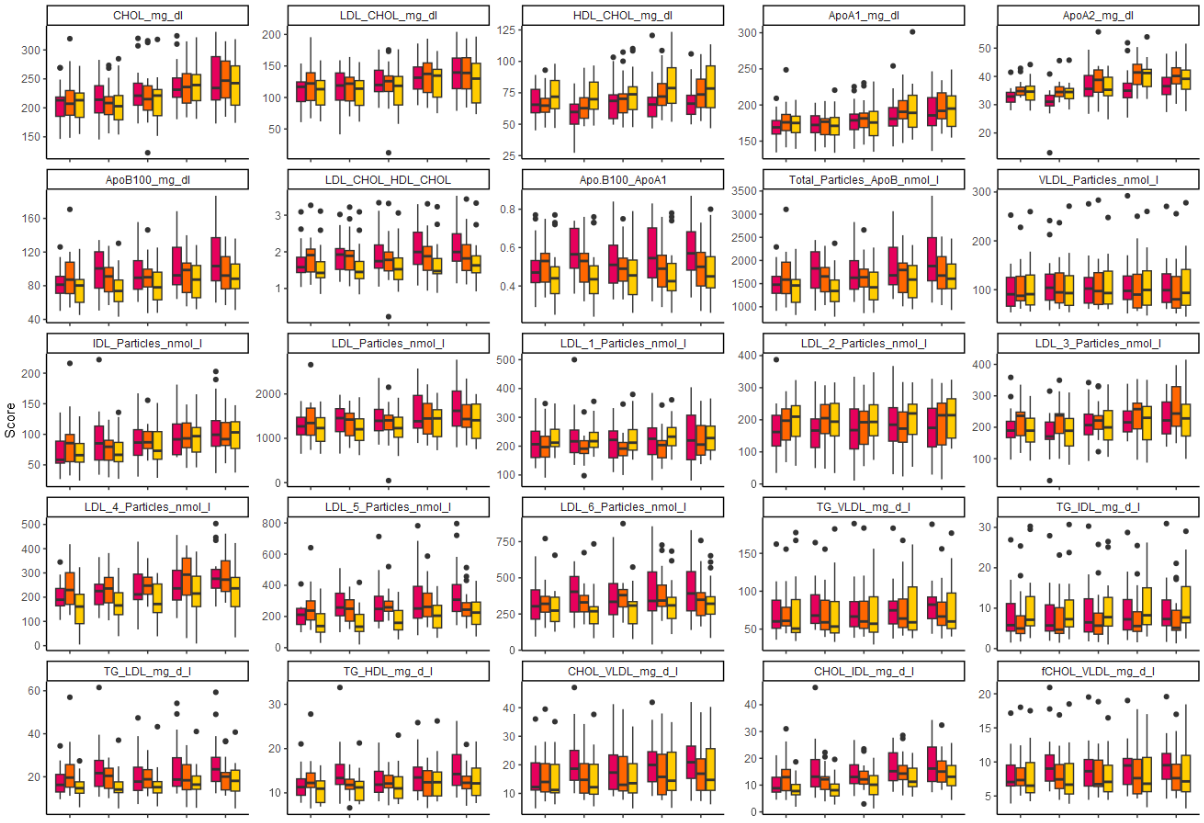
Score
CHOL_mg_dl
LDL_CHOL_mg_dl
HDL_CHOL_mg_dl
ApoA1_mg_dl
ApoA2_mg_dl
ApoB100_mg_dl
LDL_CHOL_HDL_CHOL
Apo.B100_ApoA1
Total_Particles_ApoB_nmol_l
VLDL_Particles_nmol_l
IDL_Particles_nmol_l
LDL_Particles_nmol_l
LDL_1_Particles_nmol_l
LDL_2_Particles_nmol_l
LDL_3_Particles_nmol_l
LDL_4_Particles_nmol_l
LDL_5_Particles_nmol_l
LDL_6_Particles_nmol_l
TG_VLDL_mg_d_l
TG_IDL_mg_d_l
TG_LDL_mg_d_l
TG_HDL_mg_d_l
CHOL_VLDL_mg_d_l
CHOL_IDL_mg_d_l
fCHOL_VLDL_mg_d_l

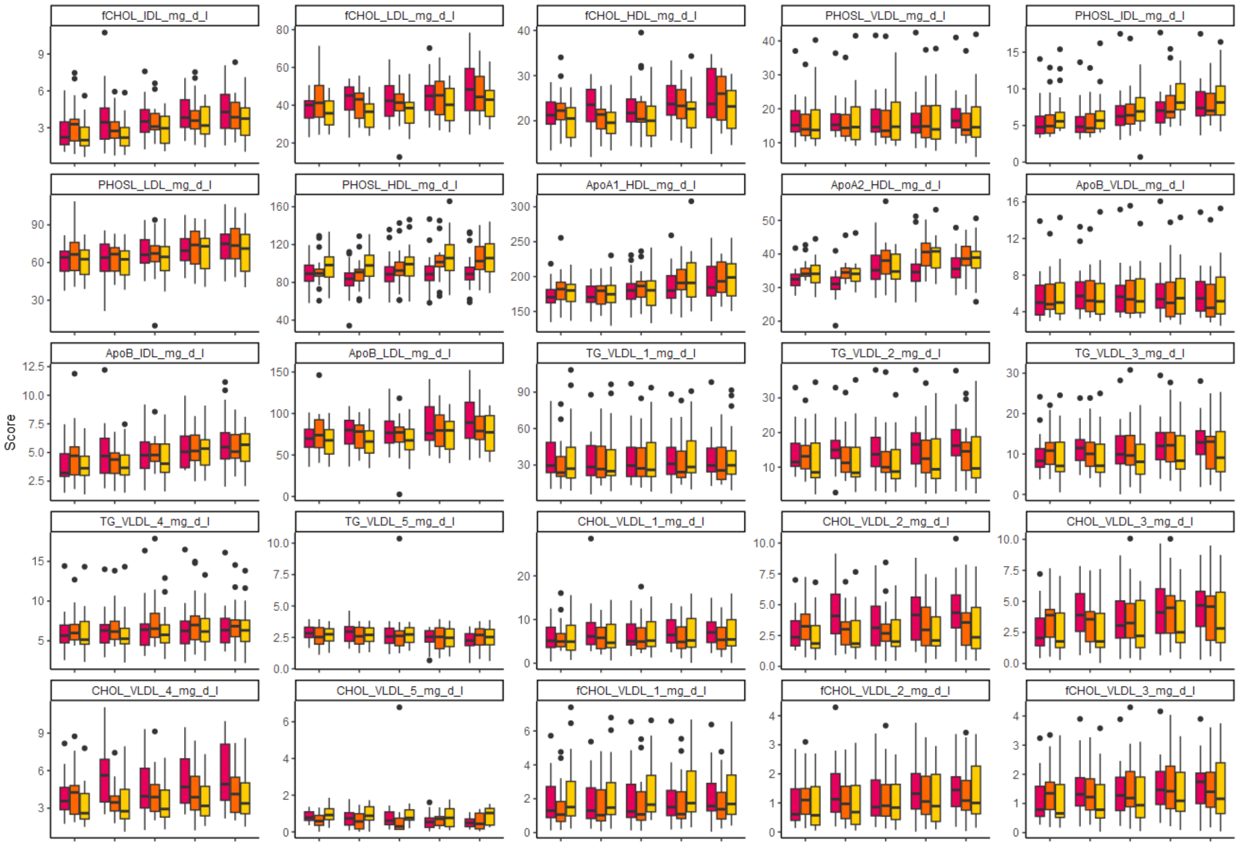
Score
fCHOL_IDL_mg_d_l
fCHOL_LDL_mg_d_l
fCHOL_HDL_mg_d_l
PHOSL_VLDL_mg_d_l
PHOSL_IDL_mg_d_l
PHOSL_LDL_mg_d_l
PHOSL_HDL_mg_d_l
ApoA1_HDL_mg_d_l
ApoA2_HDL_mg_d_l
ApoB_VLDL_mg_d_l
ApoB_IDL_mg_d_l
ApoB_LDL_mg_d_l
TG_VLDL_1_mg_d_l
TG_VLDL_2_mg_d_l
TG_VLDL_3_mg_d_l
TG_VLDL_4_mg_d_l
TG_VLDL_5_mg_d_l
CHOL_VLDL_1_mg_d_l
CHOL_VLDL_2_mg_d_l
CHOL_VLDL_3_mg_d_l
CHOL_VLDL_4_mg_d_l
CHOL_VLDL_5_mg_d_l
fCHOL_VLDL_1_mg_d_l
fCHOL_VLDL_2_mg_d_l
fCHOL_VLDL_3_mg_d_l

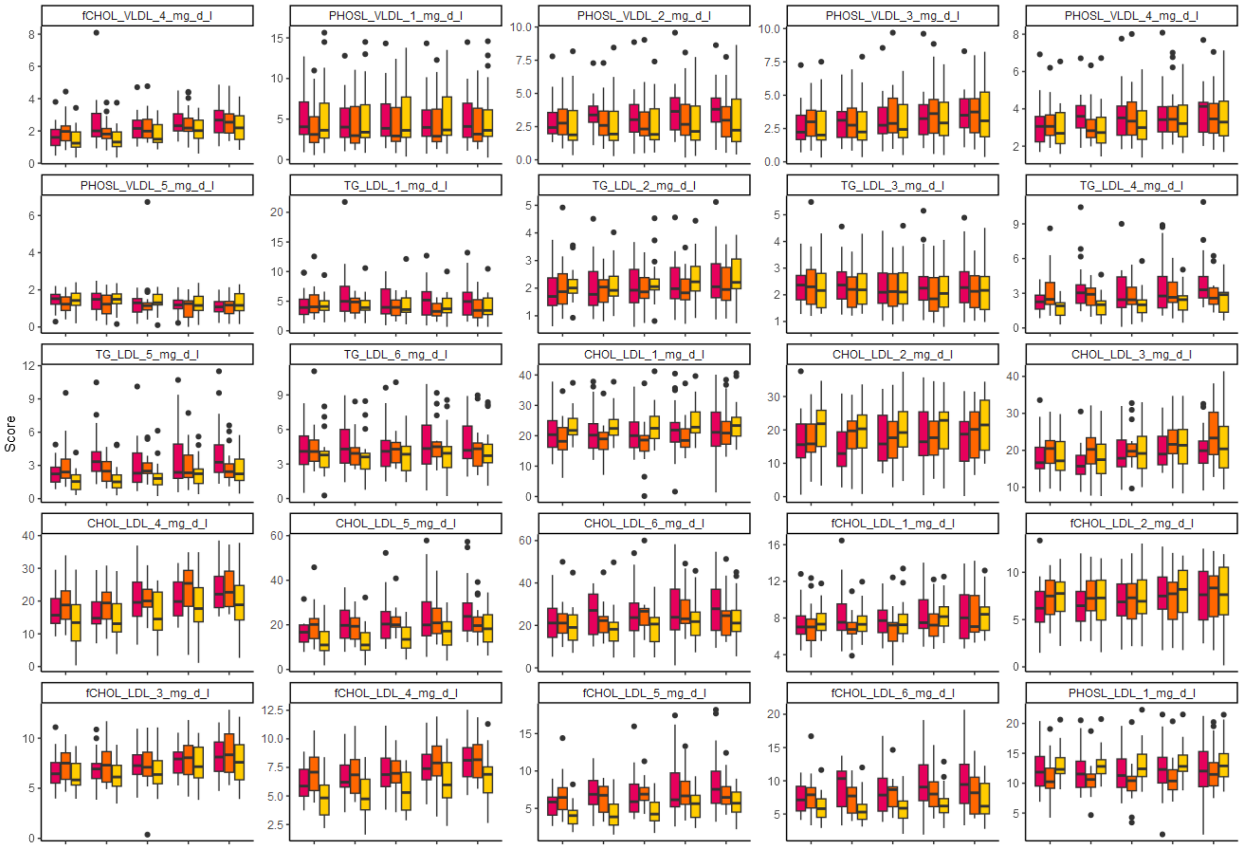
fCHOL_VLDL_4_mg_d_l
PHOSL_VLDL_1_mg_d_l
PHOSL_VLDL_2_mg_d_l
PHOSL_VLDL_3_mg_d_l
PHOSL_VLDL_4_mg_d_l
PHOSL_VLDL_5_mg_d_l
TG_LDL_1_mg_d_l
TG_LDL_2_mg_d_l
TG_LDL_3_mg_d_l
TG_LDL_4_mg_d_l
TG_LDL_5_mg_d_l
TG_LDL_6_mg_d_l
CHOL_LDL_1_mg_d_l
CHOL_LDL_2_mg_d_l
CHOL_LDL_3_mg_d_l
CHOL_LDL_4_mg_d_l
CHOL_LDL_5_mg_d_l
CHOL_LDL_6_mg_d_l
fCHOL_LDL_1_mg_d_l
fCHOL_LDL_2_mg_d_l
fCHOL_LDL_3_mg_d_l
fCHOL_LDL_4_mg_d_l
fCHOL_LDL_5_mg_d_l
fCHOL_LDL_6_mg_d_l
PHOSL_LDL_1_mg_d_l
Score

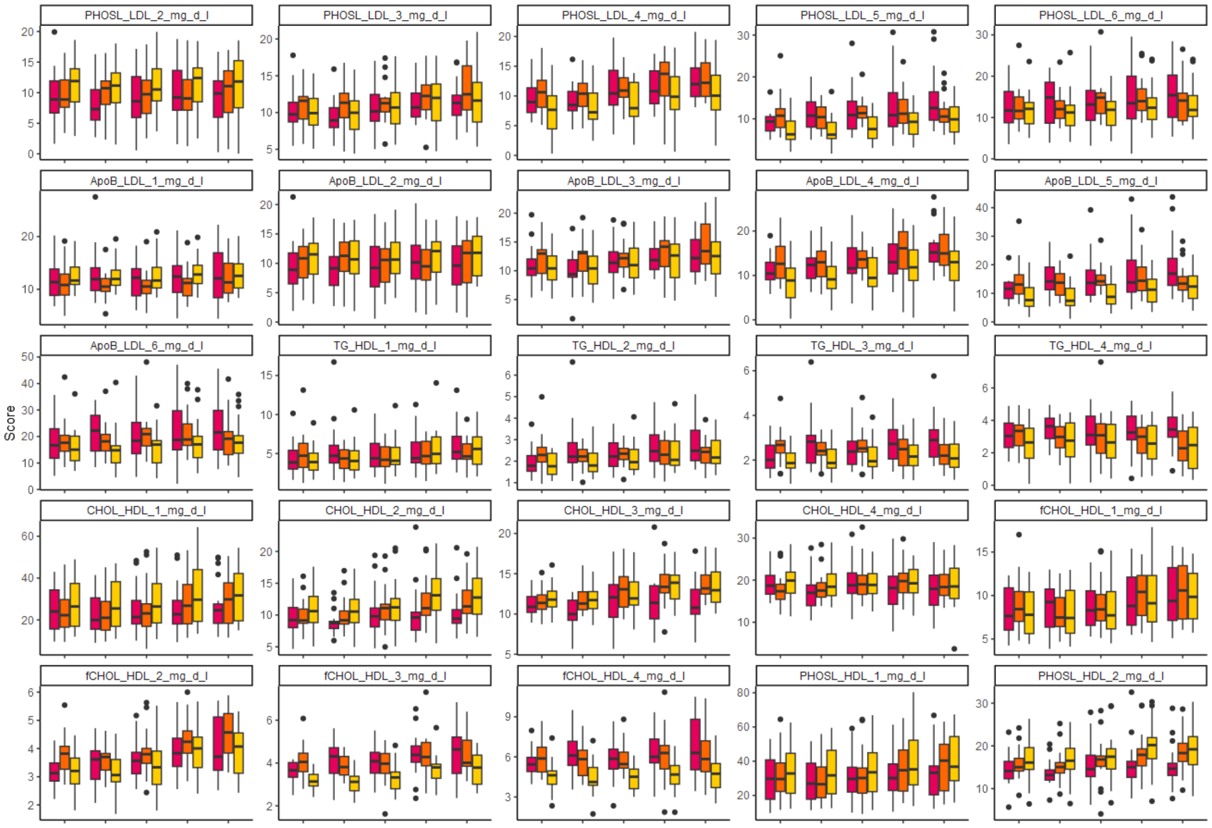
PHOSL_LDL_2_mg_d_l
PHOSL_LDL_3_mg_d_l
PHOSL_LDL_4_mg_d_l
PHOSL_LDL_5_mg_d_l
PHOSL_LDL_6_mg_d_l
ApoB_LDL_1_mg_d_l
ApoB_LDL_2_mg_d_l
ApoB_LDL_3_mg_d_l
ApoB_LDL_4_mg_d_l
ApoB_LDL_5_mg_d_l
ApoB_LDL_6_mg_d_l
TG_HDL_1_mg_d_l
TG_HDL_2_mg_d_l
TG_HDL_3_mg_d_l
TG_HDL_4_mg_d_l
CHOL_HDL_1_mg_d_l
CHOL_HDL_2_mg_d_l
CHOL_HDL_3_mg_d_l
CHOL_HDL_4_mg_d_l
fCHOL_HDL_1_mg_d_l
fCHOL_HDL_2_mg_d_l
fCHOL_HDL_3_mg_d_l
fCHOL_HDL_4_mg_d_l
PHOSL_HDL_1_mg_d_l
PHOSL_HDL_2_mg_d_l
Score

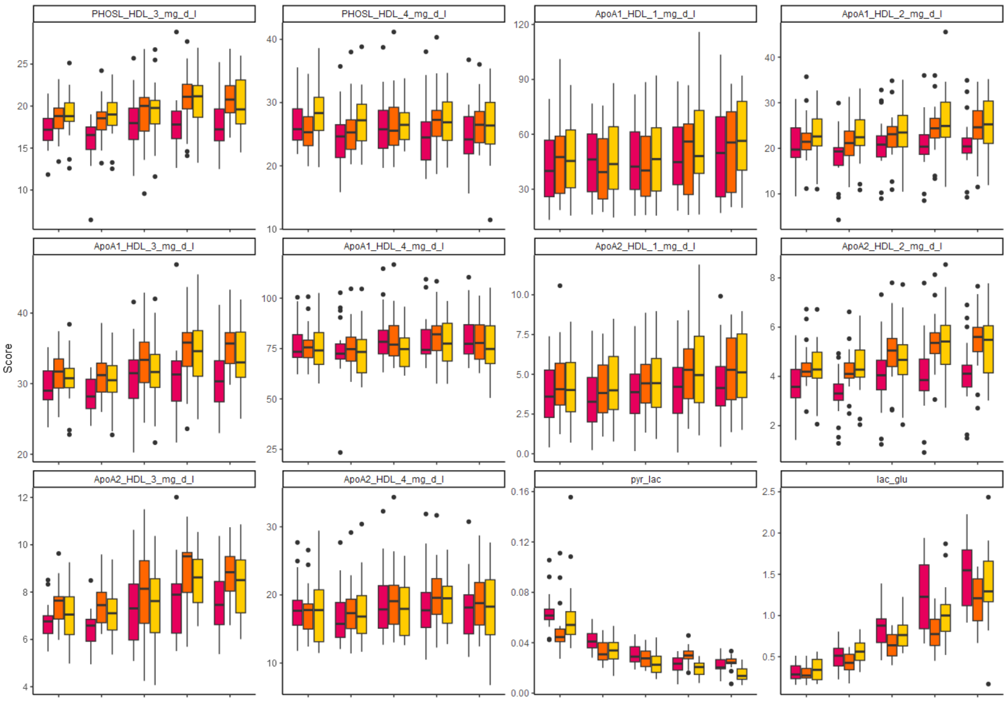
PHOSL_HDL_3_mg_d_l
PHOSL_HDL_4_mg_d_l
ApoA1_HDL_1_mg_d_l
ApoA1_HDL_2_mg_d_l
ApoA1_HDL_3_mg_d_l
ApoA1_HDL_4_mg_d_l
ApoA2_HDL_1_mg_d_l
ApoA2_HDL_2_mg_d_l
ApoA2_HDL_3_mg_d_l
ApoA2_HDL_4_mg_d_l
pyr_lac
lac_glu
Score

Figure S3: Overview of alanine fitting in serum for each donor. The blue line representing standard linear models and the green the linear mixed models with intercept set as a random effect.

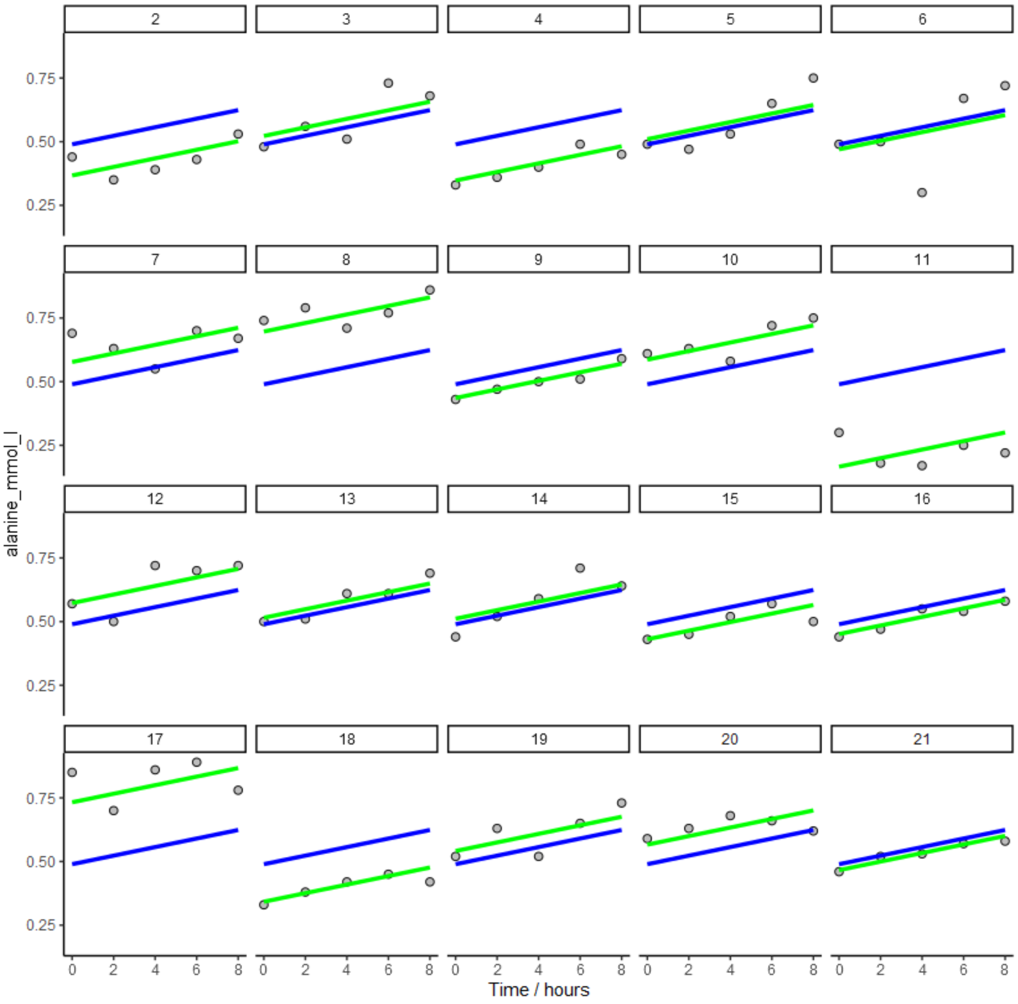

Figure S4: Timeline plots showing the time to 20% change per parameter for post-centrifugation delay. Letters are correspond to the SPREC classification (B <1h, D <2h, F 2-8h).

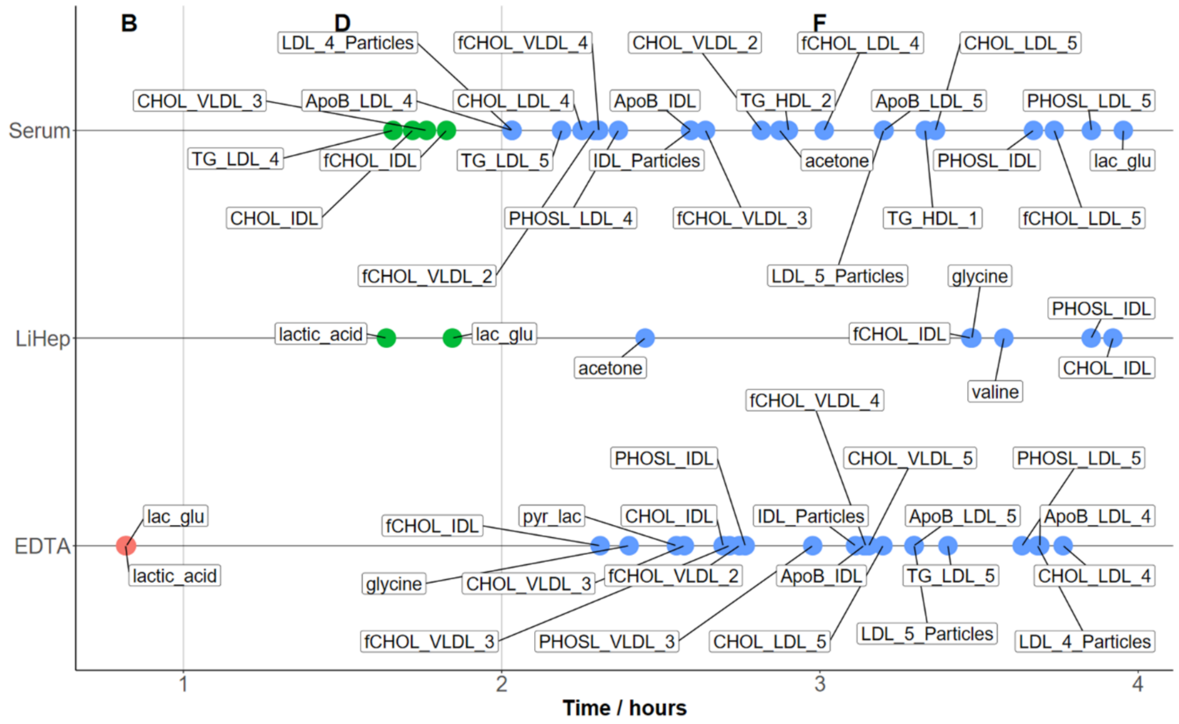

Table S1: Differences in the naming of lipid parameters between official Bruker names and the names used in this study.

Bruker Name	Name	Bruker Name	Name
TPTG [mg/dL]	TG_mg_dl	V1TG [mg/dL]	TG_VLDL-1_mg_dl
TPCH [mg/dL]	CHOL_mg_dl	V2TG [mg/dL]	TG_VLDL-2_mg_dl
LDCH [mg/dL]	LDL-CHOL_mg_dl	V3TG [mg/dL]	TG_VLDL-3_mg_dl
HDCH [mg/dL]	HDL-CHOL_mg_dl	V4TG [mg/dL]	TG_VLDL-4_mg_dl
TPA1 [mg/dL]	Apo-A1_mg_dl	V5TG [mg/dL]	TG_VLDL-5_mg_dl
TPA2 [mg/dL]	Apo-A2_mg_dl	V1CH [mg/dL]	CHOL_VLDL-1_mg_dl
TPAB [mg/dL]	Apo-B100_mg_dl	V2CH [mg/dL]	CHOL_VLDL-2_mg_dl
LDHD [-/-]	LDL-CHOL_HDL-CHOL	V3CH [mg/dL]	CHOL_VLDL-3_mg_dl
ABA1 [-/-]	Apo-B100_Apo-A1	V4CH [mg/dL]	CHOL_VLDL-4_mg_dl
TBPN [nmol/L]	Total_Particles_ApoB_nmol_l	V5CH [mg/dL]	CHOL_VLDL-5_mg_dl
VLPN [nmol/L]	VLDL-Particles_nmol_l	V1FC [mg/dL]	fCHOL_VLDL-1_mg_dl
IDPN [nmol/L]	IDL-Particles_nmol_l	V2FC [mg/dL]	fCHOL_VLDL-2_mg_dl
LDPN [nmol/L]	LDL-Particles_nmol_l	V3FC [mg/dL]	fCHOL_VLDL-3_mg_dl
L1PN [nmol/L]	LDL-1-Particles_nmol_l	V4FC [mg/dL]	fCHOL_VLDL-4_mg_dl
L2PN [nmol/L]	LDL-2-Particles_nmol_l	V5FC [mg/dL]	fCHOL_VLDL-5_mg_dl
L3PN [nmol/L]	LDL-3-Particles_nmol_l	V1PL [mg/dL]	PHOSL_VLDL-1_mg_dl
L4PN [nmol/L]	LDL-4-Particles_nmol_l	V2PL [mg/dL]	PHOSL_VLDL-2_mg_dl
L5PN [nmol/L]	LDL-5-Particles_nmol_l	V3PL [mg/dL]	PHOSL_VLDL-3_mg_dl
L6PN [nmol/L]	LDL-6-Particles_nmol_l	V4PL [mg/dL]	PHOSL_VLDL-4_mg_dl
VLTG [mg/dL]	TG_VLDL_mg_dl	V5PL [mg/dL]	PHOSL_VLDL-5_mg_dl
IDTG [mg/dL]	TG_IDL_mg_dl	L1TG [mg/dL]	TG_LDL-1_mg_dl
LDTG [mg/dL]	TG_LDL_mg_dl	L2TG [mg/dL]	TG_LDL-2_mg_dl
HDTG [mg/dL]	TG_HDL_mg_dl	L3TG [mg/dL]	TG_LDL-3_mg_dl
VLCH [mg/dL]	CHOL_VLDL_mg_dl	L4TG [mg/dL]	TG_LDL-4_mg_dl
IDCH [mg/dL]	CHOL_IDL_mg_dl	L5TG [mg/dL]	TG_LDL-5_mg_dl
VLFC [mg/dL]	fCHOL_VLDL_mg_dl	L6TG [mg/dL]	TG_LDL-6_mg_dl
IDFC [mg/dL]	fCHOL_IDL_mg_dl	L1CH [mg/dL]	CHOL_LDL-1_mg_dl
LDFC [mg/dL]	fCHOL_LDL_mg_dl	L2CH [mg/dL]	CHOL_LDL-2_mg_dl
HDFC [mg/dL]	fCHOL_HDL_mg_dl	L3CH [mg/dL]	CHOL_LDL-3_mg_dl
VLPL [mg/dL]	PHOSL_VLDL_mg_dl	L4CH [mg/dL]	CHOL_LDL-4_mg_dl
IDPL [mg/dL]	PHOSL_IDL_mg_dl	L5CH [mg/dL]	CHOL_LDL-5_mg_dl
LDPL [mg/dL]	PHOSL_LDL_mg_dl	L6CH [mg/dL]	CHOL_LDL-6_mg_dl
HDPL [mg/dL]	PHOSL_HDL_mg_dl	L1FC [mg/dL]	fCHOL_LDL-1_mg_dl
HDA1 [mg/dL]	ApoA1_HDL_mg_dl	L2FC [mg/dL]	fCHOL_LDL-2_mg_dl
HDA2 [mg/dL]	ApoA2_HDL_mg_dl	L3FC [mg/dL]	fCHOL_LDL-3_mg_dl
VLAB [mg/dL]	ApoB_VLDL_mg_dl	L4FC [mg/dL]	fCHOL_LDL-4_mg_dl
IDAB [mg/dL]	ApoB_IDL_mg_dl	L5FC [mg/dL]	fCHOL_LDL-5_mg_dl
LDAB [mg/dL]	ApoB_LDL_mg_dl	L6FC [mg/dL]	fCHOL_LDL-6_mg_dl
H1CH [mg/dL]	CHOL_HDL-1_mg_dl	L1PL [mg/dL]	PHOSL_LDL-1_mg_dl
H2CH [mg/dL]	CHOL_HDL-2_mg_dl	L2PL [mg/dL]	PHOSL_LDL-2_mg_dl
H3CH [mg/dL]	CHOL_HDL-3_mg_dl	L3PL [mg/dL]	PHOSL_LDL-3_mg_dl
H4CH [mg/dL]	CHOL_HDL-4_mg_dl	L4PL [mg/dL]	PHOSL_LDL-4_mg_dl
H1FC [mg/dL]	fCHOL_HDL-1_mg_dl	L5PL [mg/dL]	PHOSL_LDL-5_mg_dl
H2FC [mg/dL]	fCHOL_HDL-2_mg_dl	L6PL [mg/dL]	PHOSL_LDL-6_mg_dl
H3FC [mg/dL]	fCHOL_HDL-3_mg_dl	L1AB [mg/dL]	ApoB_LDL-1_mg_dl
H4FC [mg/dL]	fCHOL_HDL-4_mg_dl	L2AB [mg/dL]	ApoB_LDL-2_mg_dl
H1PL [mg/dL]	PHOSL_HDL-1_mg_dl	L3AB [mg/dL]	ApoB_LDL-3_mg_dl
H2PL [mg/dL]	PHOSL_HDL-2_mg_dl	L4AB [mg/dL]	ApoB_LDL-4_mg_dl
H3PL [mg/dL]	PHOSL_HDL-3_mg_dl	L5AB [mg/dL]	ApoB_LDL-5_mg_dl
H4PL [mg/dL]	PHOSL_HDL-4_mg_dl	L6AB [mg/dL]	ApoB_LDL-6_mg_dl
H1A1 [mg/dL]	ApoA1_HDL-1_mg_dl	H1TG [mg/dL]	TG_HDL-1_mg_dl
H2A1 [mg/dL]	ApoA1_HDL-2_mg_dl	H2TG [mg/dL]	TG_HDL-2_mg_dl
H3A1 [mg/dL]	ApoA1_HDL-3_mg_dl	H3TG [mg/dL]	TG_HDL-3_mg_dl

H4A1 [mg/dL]	ApoA1_HDL-4_mg_dl	H4TG [mg/dL]	TG_HDL-4_mg_dl
H1A2 [mg/dL]	ApoA2_HDL-1_mg_dl		
H2A2 [mg/dL]	ApoA2_HDL-2_mg_dl		
H3A2 [mg/dL]	ApoA2_HDL-3_mg_dl		
H4A2 [mg/dL]	ApoA2_HDL-4_mg_dl		

Table S2: Overview of samples used in the DILB cohort (EDTA and serum samples combined). Pre-centrifugation corresponds to SPREC letter 2 and post-centrifugation corresponds to SPREC letter 6.

Pre-Centrifugation		Post-Centrifugation	
A	3887	B	3164
A1	278	D	2275
C	1875	F	1074
E	444		
G	29		

Table S3: Blood cell count for each donor from the model cohort in GPt/L.

Donor	WBC	RBC	Total
2	5.68	5.9	11.58
3	4.81	5.58	10.39
4	6.47	4.47	10.94
5	7.45	4.95	12.4
6	5.65	4.96	10.61
7	7.7	4.74	12.44
8	4.19	4.16	8.35
9	4.27	4.79	9.06
10	7.57	4.61	12.18
11	9.17	4.44	13.61
12	9.13	4.86	13.99
13	5.23	4.75	9.98
14	5.27	4.5	9.77
15	5.42	4.86	10.28
16	4.34	4.9	9.24
17	5.04	5.26	10.3
18	4.83	4.76	9.59
19	8.94	4.98	13.92
20	2.76	4.3	7.06
21	4.78	5.31	10.09

Table S4: Model information with and without cells as a random factor for the linear mixed models of the pre-centrifugation experiment.

Parameter	Type	Cells | Donor				1 | Donor			
		conr2	margr2	intercept	slope	conr2	margr2	intercept	slope
alanine	EDTA	0.981	0.008	0.436	0.011	0.874	0.050	0.437	0.011
creatinine	EDTA	NA	0.001	0.113	0.000	0.535	0.000	0.115	0.000
glutamine	EDTA	0.931	0.002	0.603	0.006	0.524	0.010	0.572	0.005
glycine	EDTA	NA	0.148	0.129	0.009	0.693	0.053	0.156	0.009
histidine	EDTA	0.892	0.000	0.080	0.000	0.431	0.000	0.081	0.000
isoleucine	EDTA	0.968	0.001	0.065	0.001	0.766	0.007	0.067	0.001
leucine	EDTA	0.503	0.071	0.113	0.003	0.857	0.020	0.111	0.003
lysine	EDTA	0.989	0.000	0.180	0.002	0.848	0.004	0.185	0.002
phenylalanine	EDTA	0.879	0.002	0.042	0.000	0.507	0.007	0.042	0.000
tyrosine	EDTA	0.989	0.000	0.066	0.001	0.849	0.005	0.066	0.001
valine	EDTA	0.995	0.001	0.261	0.005	0.936	0.018	0.270	0.005
citric_acid	EDTA	0.836	0.010	0.165	0.003	0.354	0.041	0.163	0.003
formic_acid	EDTA	0.738	0.000	0.029	0.000	0.174	0.000	0.030	0.000
lactic_acid	EDTA	0.978	0.111	1.455	0.490	0.872	0.660	1.546	0.490
acetone	EDTA	0.408	0.012	0.036	0.001	0.782	0.004	0.036	0.001
pyruvic_acid	EDTA	NA	0.064	0.099	0.002	0.527	0.027	0.098	0.002
glucose	EDTA	0.917	0.079	5.156	-0.167	0.834	0.158	5.137	-0.167
TG	EDTA	NA	0.049	100.861	1.383	0.812	0.010	112.408	1.383
CHOL	EDTA	0.990	0.008	204.092	5.068	0.838	0.122	206.990	5.068
LDL_CHOL	EDTA	0.806	0.113	109.546	3.782	0.802	0.115	109.538	3.782
HDL_CHOL	EDTA	NA	0.038	63.792	0.584	0.702	0.012	63.664	0.584
ApoA1	EDTA	NA	0.301	168.196	2.728	0.760	0.103	168.125	2.728
ApoA2	EDTA	NA	0.199	31.517	0.632	0.586	0.103	31.962	0.632
ApoB100	EDTA	0.975	0.005	85.445	2.878	0.593	0.082	86.089	2.878
LDL_CHOL_HDL_CHOL	EDTA	0.288	0.103	1.752	0.046	0.652	0.050	1.766	0.046
Apo.B100_ApoA1	EDTA	0.906	0.006	0.510	0.008	0.630	0.025	0.515	0.008
Total_Particles_ApoB	EDTA	0.975	0.005	1553.523	52.325	0.593	0.082	1565.313	52.325
VLDL_Particles	EDTA	NA	0.016	94.303	0.758	0.888	0.002	106.728	0.758
IDL_Particles	EDTA	0.973	0.006	71.432	4.320	0.515	0.098	73.999	4.320
LDL_Particles	EDTA	0.988	0.003	1291.396	43.489	0.696	0.082	1283.717	43.489
LDL_1_Particles	EDTA	NA	0.011	216.599	1.707	0.672	0.004	219.306	1.707
LDL_2_Particles	EDTA	0.902	0.005	167.246	2.420	0.867	0.007	163.888	2.422
LDL_3_Particles	EDTA	NA	0.199	184.769	5.264	0.760	0.060	187.620	5.264
LDL_4_Particles	EDTA	NA	0.202	198.107	10.796	0.486	0.127	199.897	10.796
LDL_5_Particles	EDTA	0.968	0.005	203.121	15.738	0.476	0.085	218.284	15.738
LDL_6_Particles	EDTA	0.970	0.003	313.042	13.075	0.545	0.040	332.372	13.075
TG_VLDL	EDTA	NA	0.081	63.903	1.224	0.863	0.012	72.388	1.224
TG_IDL	EDTA	NA	0.120	6.295	0.140	0.973	0.004	8.112	0.140
TG_LDL	EDTA	0.971	0.002	19.594	0.820	0.472	0.043	19.359	0.820
TG_HDL	EDTA	0.948	0.005	12.400	0.356	0.528	0.046	12.527	0.356
CHOL_VLDL	EDTA	NA	0.068	15.949	0.476	0.678	0.023	17.600	0.476
CHOL_IDL	EDTA	0.866	0.021	11.599	0.793	0.424	0.091	11.963	0.793
fCHOL_VLDL	EDTA	NA	0.026	8.054	0.107	0.706	0.008	8.734	0.107
fCHOL_IDL	EDTA	0.908	0.017	2.773	0.206	0.510	0.092	2.947	0.206
fCHOL_LDL	EDTA	NA	0.224	40.573	1.189	0.667	0.096	38.823	1.189
fCHOL_HDL	EDTA	0.798	0.025	21.554	0.364	0.684	0.039	21.626	0.364
PHOSL_VLDL	EDTA	NA	0.016	16.445	0.082	0.928	0.001	17.016	0.082
PHOSL_IDL	EDTA	0.984	0.012	5.085	0.323	0.876	0.092	5.362	0.323
PHOSL_LDL	EDTA	0.990	0.005	60.594	1.692	0.804	0.095	60.928	1.692
PHOSL_HDL	EDTA	NA	0.047	86.188	0.690	0.786	0.011	86.319	0.690
ApoA1_HDL	EDTA	NA	0.302	169.308	2.799	0.786	0.093	169.303	2.799
ApoA2_HDL	EDTA	0.821	0.046	31.397	0.576	0.623	0.096	31.900	0.576

ApoB_VLDL	EDTA	NA	0.016	5.187	0.042	0.889	0.002	5.870	0.042
ApoB_IDL	EDTA	NA	0.172	3.929	0.238	0.515	0.098	4.070	0.238
ApoB_LDL	EDTA	0.988	0.003	71.024	2.392	0.696	0.082	70.601	2.392
TG_VLDL_1	EDTA	0.929	0.000	33.761	0.063	0.916	0.000	35.600	0.063
TG_VLDL_2	EDTA	0.935	0.007	12.704	0.447	0.700	0.030	13.840	0.447
TG_VLDL_3	EDTA	0.965	0.006	9.133	0.395	0.759	0.039	9.987	0.395
TG_VLDL_4	EDTA	0.996	0.000	5.869	0.062	0.898	0.004	6.260	0.062
TG_VLDL_5	EDTA	0.957	0.011	2.857	-0.076	0.706	0.075	2.901	-0.076
CHOL_VLDL_1	EDTA	NA	0.011	6.052	0.099	0.583	0.005	6.537	0.099
CHOL_VLDL_2	EDTA	0.784	0.021	3.085	0.180	0.477	0.051	3.179	0.180
CHOL_VLDL_3	EDTA	0.891	0.020	2.745	0.214	0.609	0.071	2.973	0.214
CHOL_VLDL_4	EDTA	NA	0.073	4.144	0.160	0.481	0.041	4.288	0.160
CHOL_VLDL_5	EDTA	0.837	0.020	0.775	-0.041	0.416	0.070	0.808	-0.040
fCHOL_VLDL_1	EDTA	NA	0.098	1.673	0.037	0.956	0.005	1.813	0.037
fCHOL_VLDL_2	EDTA	NA	0.094	0.816	0.057	0.695	0.031	1.063	0.057
fCHOL_VLDL_3	EDTA	0.939	0.009	1.011	0.064	0.720	0.040	1.188	0.064
fCHOL_VLDL_4	EDTA	0.607	0.039	1.821	0.097	0.373	0.061	1.946	0.097
PHOSL_VLDL_1	EDTA	NA	0.001	4.616	0.008	0.942	0.000	4.961	0.008
PHOSL_VLDL_2	EDTA	0.953	0.006	2.793	0.106	0.780	0.030	3.019	0.106
PHOSL_VLDL_3	EDTA	0.971	0.010	2.432	0.135	0.859	0.051	2.664	0.135
PHOSL_VLDL_4	EDTA	NA	0.131	3.045	0.073	0.843	0.024	3.289	0.073
PHOSL_VLDL_5	EDTA	0.988	0.004	1.370	-0.058	0.785	0.077	1.466	-0.058
TG_LDL_1	EDTA	0.863	0.000	4.475	0.042	0.537	0.001	4.959	0.042
TG_LDL_2	EDTA	0.936	0.004	1.928	0.043	0.737	0.018	1.943	0.043
TG_LDL_3	EDTA	NA	0.008	2.393	0.009	0.908	0.001	2.370	0.009
TG_LDL_4	EDTA	0.957	0.004	2.544	0.169	0.393	0.053	2.623	0.169
TG_LDL_5	EDTA	0.564	0.036	2.659	0.192	0.414	0.049	2.659	0.192
TG_LDL_6	EDTA	0.983	0.001	4.085	0.091	0.784	0.014	4.222	0.091
CHOL_LDL_1	EDTA	NA	0.012	21.306	0.158	0.744	0.003	21.266	0.158
CHOL_LDL_2	EDTA	NA	0.025	16.218	0.201	0.824	0.005	15.508	0.202
CHOL_LDL_3	EDTA	NA	0.100	17.183	0.386	0.696	0.034	17.372	0.386
CHOL_LDL_4	EDTA	0.877	0.040	16.211	0.824	0.624	0.122	16.296	0.824
CHOL_LDL_5	EDTA	0.970	0.006	15.710	1.207	0.496	0.095	16.868	1.207
CHOL_LDL_6	EDTA	0.974	0.003	20.999	0.887	0.612	0.041	22.277	0.887
fCHOL_LDL_1	EDTA	0.089	0.060	7.533	0.139	0.672	0.021	7.562	0.139
fCHOL_LDL_2	EDTA	NA	0.152	6.408	0.160	0.863	0.025	6.335	0.160
fCHOL_LDL_3	EDTA	0.585	0.142	6.678	0.177	0.784	0.074	6.691	0.177
fCHOL_LDL_4	EDTA	0.947	0.017	6.158	0.270	0.523	0.155	6.103	0.270
fCHOL_LDL_5	EDTA	0.962	0.006	5.470	0.347	0.444	0.087	5.726	0.347
fCHOL_LDL_6	EDTA	0.955	0.004	7.504	0.315	0.428	0.052	7.643	0.315
PHOSL_LDL_1	EDTA	0.353	0.003	12.114	0.049	0.735	0.001	12.150	0.049
PHOSL_LDL_2	EDTA	NA	0.007	9.235	0.058	0.787	0.001	8.838	0.058
PHOSL_LDL_3	EDTA	NA	0.122	9.698	0.197	0.725	0.038	9.797	0.197
PHOSL_LDL_4	EDTA	0.930	0.021	9.082	0.410	0.622	0.115	9.105	0.410
PHOSL_LDL_5	EDTA	0.965	0.006	8.782	0.610	0.486	0.089	9.335	0.610
PHOSL_LDL_6	EDTA	0.444	0.056	12.304	0.411	0.654	0.035	12.467	0.411
ApoB_LDL_1	EDTA	NA	0.011	11.912	0.094	0.672	0.004	12.062	0.094
ApoB_LDL_2	EDTA	0.903	0.005	9.198	0.133	0.867	0.007	9.014	0.133
ApoB_LDL_3	EDTA	NA	0.199	10.161	0.290	0.760	0.060	10.318	0.290
ApoB_LDL_4	EDTA	NA	0.202	10.895	0.594	0.486	0.127	10.993	0.594
ApoB_LDL_5	EDTA	0.968	0.005	11.170	0.866	0.476	0.085	12.005	0.866
ApoB_LDL_6	EDTA	0.970	0.003	17.215	0.719	0.545	0.040	18.280	0.719
TG_HDL_1	EDTA	0.969	0.003	4.540	0.159	0.659	0.031	4.607	0.159
TG_HDL_2	EDTA	0.910	0.012	2.117	0.089	0.462	0.070	2.122	0.089
TG_HDL_3	EDTA	0.850	0.013	2.318	0.084	0.360	0.057	2.312	0.084
TG_HDL_4	EDTA	0.535	0.009	3.157	0.030	0.711	0.005	3.191	0.030
CHOL_HDL_1	EDTA	NA	0.038	23.553	0.258	0.889	0.004	23.716	0.258
CHOL_HDL_2	EDTA	0.971	0.001	8.932	0.136	0.711	0.014	9.304	0.136

CHOL_HDL_3	EDTA	0.873	0.006	10.769	0.114	0.641	0.017	10.768	0.114
CHOL_HDL_4	EDTA	0.816	0.004	18.421	-0.129	0.748	0.006	18.913	-0.129
fCHOL_HDL_1	EDTA	0.879	0.038	8.074	0.235	0.850	0.047	8.093	0.235
fCHOL_HDL_2	EDTA	NA	0.249	3.166	0.101	0.654	0.115	3.193	0.101
fCHOL_HDL_3	EDTA	NA	0.150	3.716	0.117	0.351	0.112	3.648	0.117
fCHOL_HDL_4	EDTA	0.100	0.057	5.578	0.108	0.444	0.035	5.683	0.108
PHOSL_HDL_1	EDTA	NA	0.092	30.170	0.393	0.937	0.006	29.760	0.393
PHOSL_HDL_2	EDTA	0.954	0.002	13.938	0.165	0.790	0.011	14.248	0.165
PHOSL_HDL_3	EDTA	NA	0.040	16.713	0.133	0.650	0.015	16.693	0.133
PHOSL_HDL_4	EDTA	0.968	0.003	25.642	-0.229	0.783	0.019	26.288	-0.229
ApoA1_HDL_1	EDTA	0.586	0.118	43.163	1.125	0.928	0.021	42.201	1.125
ApoA1_HDL_2	EDTA	0.863	0.007	20.246	0.190	0.825	0.008	19.606	0.190
ApoA1_HDL_3	EDTA	NA	0.089	29.090	0.258	0.696	0.030	28.971	0.258
ApoA1_HDL_4	EDTA	0.864	0.009	73.959	0.643	0.716	0.019	76.124	0.643
ApoA2_HDL_1	EDTA	NA	0.264	3.801	0.103	0.953	0.017	3.629	0.103
ApoA2_HDL_2	EDTA	0.473	0.103	3.508	0.089	0.807	0.038	3.470	0.089
ApoA2_HDL_3	EDTA	0.491	0.148	6.553	0.147	0.677	0.094	6.567	0.147
ApoA2_HDL_4	EDTA	NA	0.016	17.151	0.072	0.884	0.002	17.732	0.072
alanine	Serum	0.975	0.015	0.490	0.017	0.829	0.098	0.491	0.017
creatinine	Serum	0.949	0.006	0.102	0.002	0.654	0.042	0.102	0.002
glutamine	Serum	0.324	0.005	0.646	0.002	0.545	0.003	0.643	0.002
glycine	Serum	0.991	0.005	0.186	0.015	0.769	0.114	0.214	0.015
histidine	Serum	0.909	0.024	0.089	0.004	0.357	0.170	0.091	0.004
isoleucine	Serum	0.953	0.003	0.062	0.001	0.747	0.015	0.062	0.002
leucine	Serum	0.937	0.024	0.120	0.005	0.854	0.057	0.121	0.005
lysine	Serum	0.993	0.003	0.211	0.006	0.890	0.053	0.215	0.006
phenylalanine	Serum	NA	0.355	0.063	0.003	0.702	0.166	0.061	0.003
tyrosine	Serum	0.988	0.004	0.064	0.002	0.841	0.055	0.065	0.002
valine	Serum	0.984	0.004	0.285	0.006	0.885	0.030	0.285	0.006
citric_acid	Serum	0.096	0.031	0.118	-0.002	0.455	0.018	0.118	-0.002
formic_acid	Serum	NA	0.005	0.026	0.000	0.142	0.004	0.026	0.000
lactic_acid	Serum	0.970	0.182	1.782	0.468	0.885	0.690	1.764	0.468
acetone	Serum	0.783	0.037	0.032	0.001	0.763	0.040	0.031	0.001
pyruvic_acid	Serum	NA	0.078	0.104	-0.002	0.731	0.023	0.102	-0.002
glucose	Serum	NA	0.228	5.229	-0.108	0.772	0.067	5.218	-0.108
TG	Serum	NA	0.135	98.496	0.995	0.974	0.004	111.295	0.995
CHOL	Serum	0.584	0.229	201.971	4.900	0.789	0.116	202.718	4.900
LDL_CHOL	Serum	0.583	0.226	104.179	2.899	0.876	0.067	104.926	2.899
HDL_CHOL	Serum	NA	0.166	70.169	1.128	0.843	0.031	70.836	1.128
ApoA1	Serum	0.522	0.168	170.168	3.102	0.726	0.096	170.150	3.102
ApoA2	Serum	0.876	0.038	33.707	0.754	0.502	0.153	33.881	0.754
ApoB100	Serum	0.981	0.012	71.815	1.834	0.920	0.049	75.432	1.834
LDL_CHOL_HDL_CHOL	Serum	NA	0.206	1.433	0.016	0.975	0.006	1.552	0.016
Apo.B100_ApoA1	Serum	NA	0.118	0.437	0.003	0.982	0.002	0.452	0.003
Total_Particles_ApoB	Serum	0.981	0.012	1305.867	33.344	0.920	0.049	1371.552	33.344
VLDL_Particles	Serum	NA	0.099	100.349	0.773	0.985	0.002	107.277	0.773
IDL_Particles	Serum	0.645	0.285	62.616	4.026	0.843	0.126	65.677	4.026
LDL_Particles	Serum	0.968	0.018	1133.279	27.729	0.923	0.043	1168.834	27.729
LDL_1_Particles	Serum	0.401	0.022	226.913	1.044	0.922	0.003	227.014	1.044
LDL_2_Particles	Serum	0.997	0.000	196.579	0.627	0.919	0.001	198.966	0.634
LDL_3_Particles	Serum	0.435	0.274	188.357	5.122	0.904	0.046	186.580	5.122
LDL_4_Particles	Serum	0.965	0.036	125.308	9.426	0.924	0.079	151.928	9.421
LDL_5_Particles	Serum	0.966	0.020	127.104	9.860	0.862	0.081	148.743	9.860
LDL_6_Particles	Serum	0.991	0.001	261.628	7.087	0.839	0.021	275.267	7.087
TG_VLDL	Serum	0.994	0.001	54.149	0.893	0.977	0.004	69.335	0.893
TG_IDL	Serum	NA	0.050	7.560	0.095	0.973	0.001	9.712	0.095
TG_LDL	Serum	0.995	0.002	14.527	0.452	0.927	0.032	15.026	0.452
TG_HDL	Serum	0.989	0.008	10.462	0.309	0.932	0.051	10.545	0.309

CHOL_VLDL	Serum	0.969	0.015	13.730	0.398	0.965	0.017	14.981	0.398
CHOL_IDL	Serum	0.915	0.071	7.592	0.692	0.827	0.144	8.025	0.692
fCHOL_VLDL	Serum	NA	0.121	7.045	0.064	0.981	0.003	7.770	0.064
fCHOL_IDL	Serum	0.622	0.323	2.112	0.185	0.851	0.127	2.260	0.185
fCHOL_LDL	Serum	0.593	0.223	34.674	0.838	0.874	0.069	34.975	0.838
fCHOL_HDL	Serum	0.560	0.163	19.843	0.366	0.875	0.046	19.626	0.366
PHOSL_VLDL	Serum	NA	0.001	15.268	0.012	0.979	0.000	17.139	0.012
PHOSL_IDL	Serum	0.638	0.177	5.680	0.327	0.822	0.087	6.257	0.327
PHOSL_LDL	Serum	0.421	0.306	58.925	1.258	0.903	0.052	59.447	1.258
PHOSL_HDL	Serum	NA	0.198	96.799	1.265	0.891	0.027	96.398	1.265
ApoA1_HDL	Serum	0.268	0.228	174.527	2.854	0.778	0.069	174.224	2.854
ApoA2_HDL	Serum	0.873	0.037	33.396	0.702	0.519	0.141	33.620	0.702
ApoB_VLDL	Serum	NA	0.099	5.519	0.043	0.985	0.002	5.900	0.043
ApoB_IDL	Serum	0.899	0.081	3.476	0.222	0.843	0.126	3.612	0.222
ApoB_LDL	Serum	0.967	0.018	62.324	1.525	0.923	0.043	64.283	1.525
TG_VLDL_1	Serum	NA	0.021	31.496	0.189	0.978	0.000	36.280	0.189
TG_VLDL_2	Serum	NA	0.072	7.719	0.132	0.973	0.002	11.759	0.132
TG_VLDL_3	Serum	NA	0.237	7.500	0.242	0.963	0.012	8.446	0.242
TG_VLDL_4	Serum	0.997	0.001	5.612	0.100	0.948	0.011	5.886	0.100
TG_VLDL_5	Serum	NA	0.179	2.713	-0.038	0.916	0.018	2.723	-0.038
CHOL_VLDL_1	Serum	NA	0.176	5.760	0.096	0.979	0.004	5.990	0.096
CHOL_VLDL_2	Serum	NA	0.233	1.776	0.090	0.943	0.017	2.478	0.090
CHOL_VLDL_3	Serum	0.759	0.203	1.852	0.167	0.950	0.042	2.389	0.167
CHOL_VLDL_4	Serum	0.874	0.059	2.812	0.099	0.947	0.025	3.150	0.099
CHOL_VLDL_5	Serum	0.986	0.004	0.920	-0.041	0.823	0.051	0.980	-0.042
fCHOL_VLDL_1	Serum	NA	0.060	2.111	0.022	0.982	0.001	2.254	0.022
fCHOL_VLDL_2	Serum	NA	0.394	0.578	0.052	0.961	0.025	0.874	0.052
fCHOL_VLDL_3	Serum	NA	0.407	0.901	0.055	0.963	0.025	1.039	0.055
fCHOL_VLDL_4	Serum	0.750	0.289	1.309	0.102	0.900	0.115	1.418	0.102
PHOSL_VLDL_1	Serum	NA	0.001	4.876	0.006	0.983	0.000	5.219	0.006
PHOSL_VLDL_2	Serum	NA	0.137	1.609	0.040	0.981	0.003	2.705	0.040
PHOSL_VLDL_3	Serum	0.962	0.020	2.203	0.107	0.956	0.023	2.656	0.107
PHOSL_VLDL_4	Serum	0.812	0.115	2.756	0.080	0.951	0.030	2.949	0.080
PHOSL_VLDL_5	Serum	0.983	0.004	1.411	-0.041	0.852	0.037	1.469	-0.041
TG_LDL_1	Serum	0.976	0.001	4.012	-0.043	0.933	0.004	4.519	-0.043
TG_LDL_2	Serum	0.923	0.030	2.085	0.041	0.937	0.025	2.106	0.041
TG_LDL_3	Serum	NA	0.060	2.296	-0.011	0.980	0.001	2.268	-0.011
TG_LDL_4	Serum	NA	0.446	1.918	0.110	0.916	0.068	1.666	0.110
TG_LDL_5	Serum	0.992	0.004	1.499	0.116	0.891	0.060	1.586	0.116
TG_LDL_6	Serum	0.991	0.001	3.383	0.058	0.937	0.008	3.635	0.058
CHOL_LDL_1	Serum	NA	0.077	23.234	0.175	0.915	0.007	23.208	0.175
CHOL_LDL_2	Serum	0.998	0.000	20.018	0.072	0.931	0.001	20.191	0.073
CHOL_LDL_3	Serum	0.335	0.276	18.006	0.511	0.891	0.045	17.686	0.511
CHOL_LDL_4	Serum	NA	0.485	11.451	0.800	0.924	0.072	13.062	0.800
CHOL_LDL_5	Serum	0.938	0.036	10.129	0.796	0.846	0.089	11.739	0.796
CHOL_LDL_6	Serum	0.483	0.083	17.299	0.540	0.838	0.026	18.654	0.540
fCHOL_LDL_1	Serum	0.703	0.052	7.521	0.103	0.866	0.023	7.505	0.103
fCHOL_LDL_2	Serum	0.510	0.033	7.317	0.083	0.887	0.008	7.087	0.083
fCHOL_LDL_3	Serum	0.685	0.143	6.195	0.175	0.855	0.066	6.177	0.175
fCHOL_LDL_4	Serum	0.971	0.029	4.494	0.248	0.879	0.118	4.686	0.248
fCHOL_LDL_5	Serum	0.972	0.017	3.633	0.213	0.843	0.094	3.902	0.213
fCHOL_LDL_6	Serum	0.975	0.003	5.517	0.143	0.763	0.030	5.746	0.143
PHOSL_LDL_1	Serum	NA	0.026	13.171	0.050	0.919	0.002	13.151	0.050
PHOSL_LDL_2	Serum	0.998	0.000	11.068	0.031	0.928	0.000	11.140	0.031
PHOSL_LDL_3	Serum	0.374	0.270	9.983	0.246	0.899	0.043	9.837	0.246
PHOSL_LDL_4	Serum	0.923	0.074	6.466	0.409	0.926	0.071	7.297	0.409
PHOSL_LDL_5	Serum	0.922	0.045	5.968	0.388	0.848	0.087	6.731	0.388
PHOSL_LDL_6	Serum	0.979	0.003	10.815	0.256	0.839	0.024	11.247	0.256

ApoB_LDL_1	Serum	0.439	0.021	12.480	0.057	0.922	0.003	12.486	0.057
ApoB_LDL_2	Serum	0.997	0.000	10.811	0.035	0.919	0.001	10.942	0.035
ApoB_LDL_3	Serum	0.448	0.268	10.359	0.282	0.904	0.046	10.261	0.282
ApoB_LDL_4	Serum	0.962	0.039	6.941	0.518	0.924	0.079	8.356	0.518
ApoB_LDL_5	Serum	0.962	0.022	7.001	0.542	0.862	0.081	8.180	0.542
ApoB_LDL_6	Serum	0.988	0.002	14.151	0.390	0.838	0.021	15.140	0.390
TG_HDL_1	Serum	0.996	0.003	4.209	0.185	0.931	0.053	4.189	0.185
TG_HDL_2	Serum	0.665	0.332	1.818	0.085	0.894	0.105	1.830	0.085
TG_HDL_3	Serum	0.613	0.155	1.915	0.046	0.923	0.031	1.951	0.046
TG_HDL_4	Serum	NA	0.135	2.566	-0.036	0.959	0.006	2.716	-0.036
CHOL_HDL_1	Serum	0.937	0.017	27.018	0.618	0.935	0.017	27.032	0.618
CHOL_HDL_2	Serum	0.956	0.019	10.334	0.325	0.865	0.058	10.608	0.325
CHOL_HDL_3	Serum	NA	0.219	11.653	0.235	0.715	0.080	11.690	0.235
CHOL_HDL_4	Serum	NA	0.011	18.721	-0.067	0.821	0.002	19.697	-0.067
fCHOL_HDL_1	Serum	0.789	0.110	7.854	0.257	0.900	0.052	7.787	0.257
fCHOL_HDL_2	Serum	0.801	0.136	3.023	0.119	0.789	0.144	3.026	0.119
fCHOL_HDL_3	Serum	0.963	0.011	3.053	0.084	0.592	0.120	3.065	0.084
fCHOL_HDL_4	Serum	0.978	0.000	4.122	0.025	0.818	0.004	4.419	0.025
PHOSL_HDL_1	Serum	NA	0.262	34.147	0.776	0.951	0.018	33.602	0.776
PHOSL_HDL_2	Serum	NA	0.327	16.239	0.414	0.901	0.048	16.291	0.414
PHOSL_HDL_3	Serum	0.797	0.042	18.712	0.232	0.807	0.040	18.709	0.232
PHOSL_HDL_4	Serum	0.802	0.028	27.349	-0.213	0.869	0.019	27.860	-0.213
ApoA1_HDL_1	Serum	0.316	0.294	47.274	1.276	0.951	0.021	45.745	1.276
ApoA1_HDL_2	Serum	NA	0.219	22.817	0.389	0.898	0.028	22.375	0.389
ApoA1_HDL_3	Serum	0.570	0.146	30.371	0.494	0.736	0.090	30.310	0.494
ApoA1_HDL_4	Serum	NA	0.021	70.751	0.248	0.855	0.003	74.925	0.248
ApoA2_HDL_1	Serum	0.338	0.338	4.352	0.149	0.941	0.030	4.222	0.149
ApoA2_HDL_2	Serum	0.751	0.121	4.233	0.147	0.803	0.096	4.221	0.147
ApoA2_HDL_3	Serum	0.512	0.195	6.944	0.189	0.655	0.138	6.948	0.189
ApoA2_HDL_4	Serum	NA	0.019	16.271	0.087	0.861	0.003	17.872	0.087
alanine	LiHep	0.732	0.197	0.346	0.026	0.711	0.212	0.346	0.026
creatinine	LiHep	NA	0.179	0.078	0.005	0.385	0.134	0.078	0.005
glutamine	LiHep	0.550	0.247	0.515	0.024	0.634	0.202	0.505	0.025
glycine	LiHep	NA	0.422	0.063	0.017	0.822	0.128	0.112	0.017
histidine	LiHep	0.971	0.007	0.079	0.004	0.591	0.105	0.085	0.004
isoleucine	LiHep	0.870	0.033	0.048	0.004	0.547	0.115	0.049	0.004
leucine	LiHep	0.747	0.064	0.100	0.006	0.658	0.086	0.100	0.006
lysine	LiHep	0.893	0.041	0.169	0.009	0.614	0.139	0.174	0.008
phenylalanine	LiHep	NA	0.109	0.041	0.001	0.592	0.048	0.042	0.001
tyrosine	LiHep	0.948	0.018	0.047	0.003	0.590	0.147	0.048	0.003
valine	LiHep	0.882	0.064	0.185	0.017	0.688	0.170	0.189	0.017
citric_acid	LiHep	NA	0.013	0.103	0.001	0.504	0.005	0.101	0.001
formic_acid	LiHep	0.928	0.037	0.057	0.005	0.568	0.222	0.059	0.005
lactic_acid	LiHep	NA	0.781	0.960	0.470	0.783	0.736	1.002	0.470
acetone	LiHep	NA	0.144	0.019	0.002	0.653	0.060	0.021	0.002
pyruvic_acid	LiHep	0.422	0.319	0.058	0.007	0.574	0.231	0.057	0.007
glucose	LiHep	NA	0.001	4.463	-0.006	0.733	0.000	4.451	-0.006
TG	LiHep	NA	0.000	97.448	0.058	0.875	0.000	109.048	0.058
CHOL	LiHep	0.662	0.101	204.449	5.093	0.636	0.109	205.746	5.093
LDL_CHOL	LiHep	0.266	0.090	113.902	2.590	0.595	0.050	115.038	2.590
HDL_CHOL	LiHep	NA	0.220	62.887	1.422	0.749	0.070	64.433	1.422
ApoA1	LiHep	NA	0.210	171.830	3.042	0.536	0.123	171.991	3.042
ApoA2	LiHep	NA	0.320	34.885	0.788	0.585	0.192	34.951	0.788
ApoB100	LiHep	0.931	0.002	87.098	0.827	0.619	0.009	89.643	0.827
LDL_CHOL_HDL_CHOL	LiHep	0.893	0.000	1.740	0.004	0.525	0.000	1.825	0.004
Apo.B100_ApoA1	LiHep	0.937	0.001	0.501	-0.003	0.668	0.004	0.520	-0.003
Total_Particles_ApoB	LiHep	0.932	0.002	1583.669	15.031	0.619	0.009	1629.939	15.031
VLDL_Particles	LiHep	NA	0.005	95.791	-0.395	0.906	0.000	108.593	-0.395

IDL_Particles	LiHep	0.726	0.031	76.723	2.541	0.553	0.051	80.188	2.541
LDL_Particles	LiHep	0.827	0.005	1328.158	16.494	0.566	0.013	1348.131	16.494
LDL_1_Particles	LiHep	0.513	0.015	193.176	2.288	0.606	0.012	192.927	2.288
LDL_2_Particles	LiHep	0.726	0.002	203.575	-0.862	0.822	0.001	189.586	-0.862
LDL_3_Particles	LiHep	0.510	0.062	211.338	5.155	0.599	0.051	212.905	5.155
LDL_4_Particles	LiHep	NA	0.095	211.706	6.542	0.543	0.048	233.978	6.542
LDL_5_Particles	LiHep	0.893	0.002	246.940	3.267	0.485	0.008	253.153	3.267
LDL_6_Particles	LiHep	0.947	0.001	329.953	3.479	0.594	0.005	344.036	3.479
TG_VLDL	LiHep	NA	0.016	58.801	0.567	0.846	0.003	70.754	0.567
TG_IDL	LiHep	NA	0.011	5.926	0.068	0.904	0.001	7.193	0.068
TG_LDL	LiHep	0.770	0.002	20.853	-0.177	0.490	0.004	20.913	-0.177
TG_HDL	LiHep	NA	0.002	12.547	-0.040	0.501	0.001	12.615	-0.040
CHOL_VLDL	LiHep	NA	0.031	14.373	0.239	0.797	0.006	16.241	0.239
CHOL_IDL	LiHep	0.925	0.008	10.795	0.456	0.548	0.047	12.004	0.456
fCHOL_VLDL	LiHep	NA	0.000	7.314	0.001	0.845	0.000	8.318	0.001
fCHOL_IDL	LiHep	0.932	0.010	2.651	0.129	0.654	0.052	3.016	0.129
fCHOL_LDL	LiHep	0.153	0.058	40.141	0.673	0.540	0.031	40.416	0.673
fCHOL_HDL	LiHep	0.920	0.006	21.046	0.345	0.498	0.038	21.486	0.345
PHOSL_VLDL	LiHep	NA	0.006	14.404	0.064	0.886	0.001	16.344	0.064
PHOSL_IDL	LiHep	0.978	0.011	5.215	0.331	0.784	0.107	5.595	0.331
PHOSL_LDL	LiHep	0.092	0.085	62.988	1.142	0.578	0.040	63.427	1.142
PHOSL_HDL	LiHep	NA	0.177	87.447	1.770	0.680	0.068	89.279	1.770
ApoA1_HDL	LiHep	NA	0.139	175.421	2.451	0.543	0.073	176.484	2.451
ApoA2_HDL	LiHep	0.970	0.013	34.623	0.704	0.597	0.168	34.719	0.704
ApoB_VLDL	LiHep	NA	0.005	5.268	-0.022	0.906	0.000	5.972	-0.022
ApoB_IDL	LiHep	0.713	0.033	4.218	0.140	0.553	0.051	4.411	0.140
ApoB_LDL	LiHep	0.826	0.005	73.046	0.907	0.566	0.013	74.144	0.907
TG_VLDL_1	LiHep	NA	0.001	28.518	0.090	0.817	0.000	32.524	0.090
TG_VLDL_2	LiHep	NA	0.006	10.667	0.094	0.775	0.001	13.256	0.094
TG_VLDL_3	LiHep	0.970	0.001	9.161	0.132	0.821	0.004	10.908	0.132
TG_VLDL_4	LiHep	0.987	0.001	6.441	0.091	0.826	0.008	6.762	0.091
TG_VLDL_5	LiHep	NA	0.001	2.621	-0.011	0.293	0.001	2.630	-0.011
CHOL_VLDL_1	LiHep	NA	0.000	5.608	0.000	0.730	0.000	6.125	0.000
CHOL_VLDL_2	LiHep	NA	0.003	2.567	0.019	0.706	0.001	3.152	0.019
CHOL_VLDL_3	LiHep	0.929	0.004	2.984	0.088	0.772	0.012	3.539	0.088
CHOL_VLDL_4	LiHep	0.888	0.003	3.497	0.057	0.681	0.008	3.891	0.057
CHOL_VLDL_5	LiHep	NA	0.013	0.716	-0.031	0.347	0.006	0.754	-0.026
fCHOL_VLDL_1	LiHep	NA	0.046	1.368	0.040	0.863	0.007	1.531	0.040
fCHOL_VLDL_2	LiHep	NA	0.024	0.818	0.022	0.810	0.005	1.138	0.022
fCHOL_VLDL_3	LiHep	0.976	0.001	0.964	0.026	0.832	0.006	1.419	0.026
fCHOL_VLDL_4	LiHep	NA	0.179	1.683	0.081	0.719	0.061	1.933	0.081
PHOSL_VLDL_1	LiHep	NA	0.005	3.651	0.034	0.811	0.001	4.263	0.034
PHOSL_VLDL_2	LiHep	NA	0.011	2.216	0.028	0.829	0.002	2.982	0.028
PHOSL_VLDL_3	LiHep	0.928	0.007	2.643	0.084	0.825	0.017	3.119	0.084
PHOSL_VLDL_4	LiHep	NA	0.121	2.959	0.070	0.842	0.022	3.241	0.070
PHOSL_VLDL_5	LiHep	0.619	0.004	1.288	-0.026	0.234	0.008	1.308	-0.026
TG_LDL_1	LiHep	0.131	0.109	4.572	-0.153	0.595	0.051	4.707	-0.153
TG_LDL_2	LiHep	0.456	0.001	1.998	0.008	0.559	0.001	1.995	0.008
TG_LDL_3	LiHep	NA	0.019	2.190	-0.030	0.466	0.010	2.326	-0.030
TG_LDL_4	LiHep	0.744	0.000	3.035	-0.014	0.449	0.001	2.960	-0.014
TG_LDL_5	LiHep	0.829	0.000	2.892	0.003	0.363	0.000	2.886	0.003
TG_LDL_6	LiHep	0.933	0.000	4.212	0.028	0.523	0.002	4.275	0.028
CHOL_LDL_1	LiHep	NA	0.083	17.441	0.451	0.611	0.035	17.522	0.451
CHOL_LDL_2	LiHep	0.447	0.003	18.480	0.089	0.826	0.001	17.607	0.092
CHOL_LDL_3	LiHep	0.495	0.106	19.137	0.635	0.614	0.081	19.090	0.635
CHOL_LDL_4	LiHep	0.916	0.016	17.586	0.656	0.604	0.075	18.886	0.656
CHOL_LDL_5	LiHep	0.889	0.004	18.954	0.360	0.491	0.017	19.480	0.360
CHOL_LDL_6	LiHep	0.944	0.002	21.751	0.392	0.600	0.014	22.542	0.392

fCHOL_LDL_1	LiHep	0.488	0.056	6.617	0.174	0.572	0.046	6.609	0.174
fCHOL_LDL_2	LiHep	0.363	0.005	7.446	0.038	0.772	0.002	7.174	0.038
fCHOL_LDL_3	LiHep	0.228	0.064	7.156	0.147	0.555	0.037	7.174	0.147
fCHOL_LDL_4	LiHep	0.952	0.007	6.407	0.173	0.568	0.062	6.752	0.173
fCHOL_LDL_5	LiHep	0.910	0.001	6.609	0.066	0.539	0.006	6.621	0.066
fCHOL_LDL_6	LiHep	0.924	0.001	7.586	0.083	0.595	0.007	7.642	0.083
PHOSL_LDL_1	LiHep	0.813	0.012	10.157	0.177	0.645	0.022	10.247	0.177
PHOSL_LDL_2	LiHep	0.111	0.005	10.277	0.040	0.835	0.001	9.732	0.040
PHOSL_LDL_3	LiHep	0.423	0.101	10.702	0.286	0.614	0.068	10.682	0.286
PHOSL_LDL_4	LiHep	0.913	0.016	9.822	0.335	0.600	0.073	10.287	0.335
PHOSL_LDL_5	LiHep	0.864	0.003	10.551	0.152	0.450	0.012	10.726	0.152
PHOSL_LDL_6	LiHep	0.936	0.002	12.552	0.205	0.570	0.015	12.817	0.205
ApoB_LDL_1	LiHep	0.512	0.015	10.623	0.126	0.606	0.012	10.609	0.126
ApoB_LDL_2	LiHep	NA	0.006	11.199	-0.047	0.822	0.001	10.426	-0.047
ApoB_LDL_3	LiHep	0.510	0.062	11.623	0.284	0.599	0.051	11.709	0.284
ApoB_LDL_4	LiHep	NA	0.095	11.643	0.360	0.543	0.048	12.869	0.360
ApoB_LDL_5	LiHep	0.893	0.002	13.581	0.180	0.485	0.008	13.923	0.180
ApoB_LDL_6	LiHep	0.947	0.001	18.143	0.191	0.594	0.005	18.921	0.191
TG_HDL_1	LiHep	0.802	0.001	4.535	0.036	0.498	0.003	4.641	0.036
TG_HDL_2	LiHep	0.791	0.000	2.297	0.003	0.539	0.000	2.308	0.003
TG_HDL_3	LiHep	NA	0.042	2.651	-0.034	0.605	0.017	2.597	-0.034
TG_HDL_4	LiHep	0.919	0.017	3.317	-0.091	0.764	0.049	3.200	-0.091
CHOL_HDL_1	LiHep	0.982	0.003	20.513	0.809	0.755	0.035	22.654	0.809
CHOL_HDL_2	LiHep	0.969	0.009	9.292	0.313	0.742	0.076	9.768	0.313
CHOL_HDL_3	LiHep	NA	0.383	11.521	0.318	0.744	0.159	11.515	0.318
CHOL_HDL_4	LiHep	0.630	0.035	17.531	0.213	0.777	0.021	18.098	0.213
fCHOL_HDL_1	LiHep	0.975	0.004	8.161	0.240	0.645	0.051	8.355	0.240
fCHOL_HDL_2	LiHep	NA	0.270	3.488	0.117	0.540	0.170	3.524	0.117
fCHOL_HDL_3	LiHep	0.804	0.015	3.848	0.069	0.355	0.049	3.875	0.069
fCHOL_HDL_4	LiHep	0.952	0.000	5.439	0.032	0.554	0.004	5.802	0.032
PHOSL_HDL_1	LiHep	0.977	0.002	27.291	0.899	0.705	0.029	29.837	0.899
PHOSL_HDL_2	LiHep	0.953	0.010	14.547	0.408	0.716	0.062	15.215	0.408
PHOSL_HDL_3	LiHep	0.601	0.136	18.156	0.370	0.699	0.103	18.149	0.370
PHOSL_HDL_4	LiHep	0.989	0.001	25.361	0.180	0.861	0.013	25.721	0.180
ApoA1_HDL_1	LiHep	0.983	0.001	42.628	0.875	0.696	0.012	44.330	0.875
ApoA1_HDL_2	LiHep	NA	0.093	20.301	0.409	0.576	0.043	21.203	0.409
ApoA1_HDL_3	LiHep	0.638	0.178	30.825	0.597	0.708	0.144	30.824	0.597
ApoA1_HDL_4	LiHep	NA	0.073	73.005	0.530	0.810	0.015	76.532	0.530
ApoA2_HDL_1	LiHep	NA	0.070	3.682	0.135	0.579	0.032	4.207	0.135
ApoA2_HDL_2	LiHep	NA	0.278	4.192	0.159	0.629	0.142	4.272	0.159
ApoA2_HDL_3	LiHep	0.983	0.008	7.295	0.198	0.657	0.161	7.372	0.198
ApoA2_HDL_4	LiHep	NA	0.131	16.610	0.252	0.839	0.024	17.777	0.252

Table S5: Model Information for the linear mixed models of the post-centrifugation experiment.

Parameter	Post-Centrifugation				Type
	conr2	margr2	Intercept	Slope	
alanine	NA	0.252	0.455	0.009	EDTA
creatinine	NA	0.010	0.111	0.001	EDTA
glutamine	NA	0.014	0.534	0.004	EDTA
glycine	0.940	0.016	0.132	0.011	EDTA
histidine	NA	0.001	0.084	0.000	EDTA
isoleucine	0.989	0.000	0.059	0.001	EDTA
leucine	0.986	0.001	0.100	0.002	EDTA
lysine	0.992	0.001	0.184	0.004	EDTA
phenylalanine	0.858	0.001	0.041	0.000	EDTA
tyrosine	0.993	0.001	0.063	0.001	EDTA
valine	0.990	0.004	0.256	0.007	EDTA
citric_acid	NA	0.132	0.158	0.004	EDTA
lactic_acid	NA	0.620	1.228	0.300	EDTA
acetone	0.977	0.001	0.031	0.001	EDTA
pyruvic_acid	0.594	0.007	0.094	0.001	EDTA
glucose	NA	0.014	5.279	-0.019	EDTA
TG	NA	0.071	101.343	1.319	EDTA
CHOL	0.533	0.254	202.964	5.041	EDTA
LDL_CHOL	0.297	0.292	108.971	3.397	EDTA
HDL_CHOL	NA	0.092	61.276	0.847	EDTA
ApoA1	NA	0.354	164.858	3.025	EDTA
ApoA2	NA	0.348	31.182	0.777	EDTA
ApoB100	0.843	0.023	83.476	2.357	EDTA
LDL_CHOL_HDL_CHOL	0.874	0.009	1.744	0.033	EDTA
Apo.B100_ApoA1	0.918	0.002	0.501	0.005	EDTA
Total_Particles_ApoB	0.839	0.024	1517.769	42.867	EDTA
VLDL_Particles	NA	0.066	91.280	1.082	EDTA
IDL_Particles	0.638	0.109	63.862	4.105	EDTA
LDL_Particles	0.823	0.030	1266.892	35.116	EDTA
LDL_1_Particles	0.005	0.005	206.313	0.790	EDTA
LDL_2_Particles	NA	0.001	172.126	0.462	EDTA
LDL_3_Particles	0.556	0.055	188.101	4.411	EDTA
LDL_4_Particles	0.632	0.094	190.121	10.322	EDTA
LDL_5_Particles	0.677	0.039	219.750	13.334	EDTA
LDL_6_Particles	0.851	0.011	327.485	12.138	EDTA
TG_VLDL	0.964	0.006	65.126	1.418	EDTA
TG_IDL	NA	0.106	6.678	0.150	EDTA
TG_LDL	0.698	0.016	18.380	0.580	EDTA
TG_HDL	NA	0.106	11.776	0.308	EDTA
CHOL_VLDL	NA	0.084	15.685	0.444	EDTA
CHOL_IDL	0.888	0.025	10.205	0.757	EDTA
fCHOL_VLDL	NA	0.049	7.921	0.105	EDTA
fCHOL_IDL	NA	0.222	2.350	0.204	EDTA
fCHOL_LDL	0.195	0.149	38.812	0.970	EDTA
fCHOL_HDL	0.551	0.039	21.758	0.316	EDTA
PHOSL_VLDL	0.919	0.001	16.132	0.116	EDTA
PHOSL_IDL	0.981	0.011	4.803	0.347	EDTA
PHOSL_LDL	0.288	0.254	60.712	1.438	EDTA
PHOSL_HDL	NA	0.107	83.215	0.830	EDTA
ApoA1_HDL	NA	0.308	165.253	2.831	EDTA
ApoA2_HDL	NA	0.338	30.910	0.720	EDTA
ApoB_VLDL	NA	0.066	5.022	0.060	EDTA

ApoB_IDL	0.543	0.137	3.542	0.226	EDTA
ApoB_LDL	0.822	0.031	69.675	1.931	EDTA
TG_VLDL_1	0.976	0.001	30.703	0.319	EDTA
TG_VLDL_2	NA	0.060	12.611	0.347	EDTA
TG_VLDL_3	NA	0.109	8.600	0.352	EDTA
TG_VLDL_4	0.997	0.001	5.855	0.124	EDTA
TG_VLDL_5	NA	0.191	2.882	-0.063	EDTA
CHOL_VLDL_1	NA	0.039	5.866	0.091	EDTA
CHOL_VLDL_2	0.742	0.014	2.899	0.135	EDTA
CHOL_VLDL_3	0.910	0.013	2.418	0.188	EDTA
CHOL_VLDL_4	NA	0.069	3.913	0.141	EDTA
CHOL_VLDL_5	0.899	0.024	0.749	-0.047	EDTA
fCHOL_VLDL_1	NA	0.109	1.583	0.050	EDTA
fCHOL_VLDL_2	NA	0.125	0.766	0.056	EDTA
fCHOL_VLDL_3	NA	0.163	0.851	0.063	EDTA
fCHOL_VLDL_4	NA	0.206	1.671	0.106	EDTA
PHOSL_VLDL_1	0.927	0.001	4.451	0.041	EDTA
PHOSL_VLDL_2	NA	0.086	2.580	0.093	EDTA
PHOSL_VLDL_3	NA	0.242	2.065	0.139	EDTA
PHOSL_VLDL_4	NA	0.208	2.941	0.089	EDTA
PHOSL_VLDL_5	0.982	0.007	1.515	-0.057	EDTA
TG_LDL_1	NA	0.000	4.019	-0.008	EDTA
TG_LDL_2	NA	0.045	1.775	0.030	EDTA
TG_LDL_3	0.934	0.001	2.263	-0.014	EDTA
TG_LDL_4	0.502	0.030	2.547	0.121	EDTA
TG_LDL_5	0.543	0.028	2.560	0.151	EDTA
TG_LDL_6	0.905	0.004	4.081	0.084	EDTA
CHOL_LDL_1	0.062	0.016	20.172	0.170	EDTA
CHOL_LDL_2	NA	0.002	15.934	0.087	EDTA
CHOL_LDL_3	NA	0.082	17.166	0.435	EDTA
CHOL_LDL_4	0.859	0.046	15.986	0.849	EDTA
CHOL_LDL_5	0.713	0.044	17.094	1.069	EDTA
CHOL_LDL_6	0.830	0.016	21.968	0.865	EDTA
fCHOL_LDL_1	0.094	0.051	7.266	0.111	EDTA
fCHOL_LDL_2	NA	0.022	6.550	0.095	EDTA
fCHOL_LDL_3	NA	0.155	6.636	0.164	EDTA
fCHOL_LDL_4	0.886	0.042	6.004	0.266	EDTA
fCHOL_LDL_5	0.720	0.034	5.831	0.291	EDTA
fCHOL_LDL_6	0.387	0.040	7.772	0.262	EDTA
PHOSL_LDL_1	0.354	0.001	11.503	0.027	EDTA
PHOSL_LDL_2	NA	0.000	9.109	0.002	EDTA
PHOSL_LDL_3	NA	0.082	9.715	0.201	EDTA
PHOSL_LDL_4	0.908	0.028	9.009	0.420	EDTA
PHOSL_LDL_5	0.626	0.050	9.506	0.523	EDTA
PHOSL_LDL_6	0.769	0.021	12.418	0.392	EDTA
ApoB_LDL_1	0.005	0.005	11.347	0.043	EDTA
ApoB_LDL_2	NA	0.001	9.467	0.026	EDTA
ApoB_LDL_3	NA	0.110	10.345	0.243	EDTA
ApoB_LDL_4	0.714	0.073	10.475	0.568	EDTA
ApoB_LDL_5	0.677	0.039	12.086	0.733	EDTA
ApoB_LDL_6	0.851	0.011	18.011	0.668	EDTA
TG_HDL_1	NA	0.207	3.523	0.156	EDTA
TG_HDL_2	NA	0.119	1.902	0.074	EDTA
TG_HDL_3	NA	0.054	2.254	0.060	EDTA
TG_HDL_4	NA	0.001	3.074	0.006	EDTA
CHOL_HDL_1	NA	0.073	19.324	0.385	EDTA
CHOL_HDL_2	0.968	0.003	8.402	0.189	EDTA
CHOL_HDL_3	NA	0.118	10.738	0.177	EDTA

CHOL_HDL_4	0.917	0.000	17.926	0.071	EDTA
fCHOL_HDL_1	NA	0.224	7.881	0.228	EDTA
fCHOL_HDL_2	NA	0.234	3.207	0.101	EDTA
fCHOL_HDL_3	NA	0.105	3.704	0.101	EDTA
fCHOL_HDL_4	NA	0.043	5.606	0.091	EDTA
PHOSL_HDL_1	NA	0.121	26.120	0.464	EDTA
PHOSL_HDL_2	0.970	0.003	12.982	0.204	EDTA
PHOSL_HDL_3	NA	0.087	16.735	0.156	EDTA
PHOSL_HDL_4	0.983	0.000	25.748	-0.054	EDTA
ApoA1_HDL_1	NA	0.205	39.894	1.011	EDTA
ApoA1_HDL_2	0.826	0.022	19.959	0.240	EDTA
ApoA1_HDL_3	NA	0.150	28.687	0.345	EDTA
ApoA1_HDL_4	0.643	0.071	75.016	0.764	EDTA
ApoA2_HDL_1	NA	0.354	3.270	0.112	EDTA
ApoA2_HDL_2	0.238	0.218	3.447	0.109	EDTA
ApoA2_HDL_3	0.540	0.210	6.490	0.175	EDTA
ApoA2_HDL_4	0.892	0.012	16.676	0.238	EDTA
pyr_lac	NA	0.504	0.063	-0.005	EDTA
lac_glu	0.798	0.264	0.265	0.065	EDTA
alanine	0.951	0.006	0.496	0.008	Serum
creatinine	0.942	0.011	0.103	0.003	Serum
glutamine	0.789	0.009	0.655	0.007	Serum
glycine	NA	0.041	0.183	0.003	Serum
histidine	0.875	0.012	0.089	0.003	Serum
isoleucine	0.964	0.004	0.061	0.002	Serum
leucine	0.954	0.003	0.124	0.002	Serum
lysine	0.992	0.001	0.220	0.002	Serum
phenylalanine	0.457	0.104	0.061	0.002	Serum
tyrosine	0.990	0.001	0.065	0.001	Serum
valine	0.991	0.002	0.280	0.005	Serum
citric_acid	0.069	0.017	0.105	-0.001	Serum
lactic_acid	0.749	0.052	1.651	0.077	Serum
acetone	0.966	0.009	0.029	0.002	Serum
pyruvic_acid	NA	0.009	0.105	0.001	Serum
glucose	NA	0.060	5.184	0.055	Serum
TG	0.987	0.002	99.989	1.301	Serum
CHOL	0.723	0.085	204.089	4.439	Serum
LDL_CHOL	0.241	0.127	106.119	2.469	Serum
HDL_CHOL	NA	0.047	70.071	0.524	Serum
ApoA1	0.138	0.132	171.566	2.261	Serum
ApoA2	0.789	0.039	32.920	0.482	Serum
ApoB100	0.969	0.013	73.584	1.994	Serum
LDL_CHOL_HDL_CHOL	NA	0.102	1.491	0.021	Serum
Apo.B100_ApoA1	0.982	0.002	0.426	0.006	Serum
Total_Particles_ApoB	0.969	0.013	1337.945	36.250	Serum
VLDL_Particles	NA	0.069	93.536	0.880	Serum
IDL_Particles	NA	0.435	61.404	4.735	Serum
LDL_Particles	0.928	0.013	1164.295	25.703	Serum
LDL_1_Particles	0.152	0.146	223.583	5.097	Serum
LDL_2_Particles	0.249	0.045	210.843	2.904	Serum
LDL_3_Particles	0.726	0.132	192.078	8.247	Serum
LDL_4_Particles	NA	0.480	130.858	12.876	Serum
LDL_5_Particles	0.952	0.013	138.506	8.657	Serum
LDL_6_Particles	0.969	0.000	270.293	2.057	Serum
TG_VLDL	NA	0.022	57.556	0.467	Serum
TG_IDL	NA	0.015	7.773	0.064	Serum
TG_LDL	0.988	0.007	14.863	0.709	Serum
TG_HDL	0.958	0.034	10.265	0.465	Serum

CHOL_VLDL	NA	0.344	14.222	0.535	Serum
CHOL_IDL	0.816	0.143	7.623	0.886	Serum
fCHOL_VLDL	NA	0.123	7.201	0.090	Serum
fCHOL_IDL	0.637	0.253	2.037	0.223	Serum
fCHOL_LDL	0.524	0.104	35.876	0.970	Serum
fCHOL_HDL	0.555	0.347	20.359	0.531	Serum
PHOSL_VLDL	NA	0.003	15.583	-0.032	Serum
PHOSL_IDL	0.965	0.014	5.762	0.314	Serum
PHOSL_LDL	0.286	0.096	59.929	1.112	Serum
PHOSL_HDL	NA	0.044	96.601	0.725	Serum
ApoA1_HDL	0.132	0.116	175.841	2.036	Serum
ApoA2_HDL	0.783	0.036	32.556	0.420	Serum
ApoB_VLDL	0.982	0.001	5.145	0.048	Serum
ApoB_IDL	NA	0.434	3.377	0.260	Serum
ApoB_LDL	0.928	0.013	64.034	1.414	Serum
TG_VLDL_1	0.982	0.000	32.446	-0.019	Serum
TG_VLDL_2	NA	0.073	8.508	0.210	Serum
TG_VLDL_3	0.983	0.005	7.560	0.271	Serum
TG_VLDL_4	0.997	0.000	5.643	0.035	Serum
TG_VLDL_5	0.044	0.000	2.674	0.005	Serum
CHOL_VLDL_1	NA	0.198	5.798	0.149	Serum
CHOL_VLDL_2	NA	0.286	1.913	0.136	Serum
CHOL_VLDL_3	NA	0.368	1.667	0.189	Serum
CHOL_VLDL_4	NA	0.284	2.870	0.137	Serum
CHOL_VLDL_5	NA	0.008	0.851	0.025	Serum
fCHOL_VLDL_1	NA	0.006	1.968	0.011	Serum
fCHOL_VLDL_2	0.981	0.010	0.634	0.055	Serum
fCHOL_VLDL_3	0.947	0.026	0.761	0.058	Serum
fCHOL_VLDL_4	NA	0.446	1.348	0.117	Serum
PHOSL_VLDL_1	NA	0.000	4.546	-0.006	Serum
PHOSL_VLDL_2	0.981	0.002	1.929	0.055	Serum
PHOSL_VLDL_3	0.870	0.034	2.182	0.094	Serum
PHOSL_VLDL_4	0.898	0.055	2.771	0.074	Serum
PHOSL_VLDL_5	0.514	0.000	1.356	-0.007	Serum
TG_LDL_1	0.984	0.001	4.290	0.118	Serum
TG_LDL_2	0.540	0.202	2.054	0.072	Serum
TG_LDL_3	NA	0.074	2.304	0.029	Serum
TG_LDL_4	0.980	0.019	1.524	0.184	Serum
TG_LDL_5	0.980	0.008	1.557	0.142	Serum
TG_LDL_6	0.990	0.000	3.414	0.030	Serum
CHOL_LDL_1	NA	0.146	22.919	0.531	Serum
CHOL_LDL_2	0.992	0.000	20.405	0.279	Serum
CHOL_LDL_3	0.723	0.106	18.380	0.750	Serum
CHOL_LDL_4	NA	0.471	11.204	0.995	Serum
CHOL_LDL_5	0.920	0.020	10.900	0.648	Serum
CHOL_LDL_6	0.970	0.000	18.177	0.142	Serum
fCHOL_LDL_1	0.202	0.196	7.559	0.241	Serum
fCHOL_LDL_2	0.282	0.069	7.483	0.170	Serum
fCHOL_LDL_3	0.522	0.154	6.445	0.218	Serum
fCHOL_LDL_4	0.961	0.036	4.569	0.303	Serum
fCHOL_LDL_5	0.960	0.013	3.902	0.209	Serum
fCHOL_LDL_6	0.948	0.003	5.881	0.118	Serum
PHOSL_LDL_1	NA	0.124	12.937	0.263	Serum
PHOSL_LDL_2	0.437	0.021	11.794	0.120	Serum
PHOSL_LDL_3	NA	0.299	10.190	0.384	Serum
PHOSL_LDL_4	NA	0.492	6.191	0.523	Serum
PHOSL_LDL_5	0.916	0.021	6.381	0.331	Serum
PHOSL_LDL_6	0.975	0.000	11.064	0.044	Serum

ApoB_LDL_1	0.151	0.146	12.297	0.280	Serum
ApoB_LDL_2	0.428	0.034	11.588	0.160	Serum
ApoB_LDL_3	NA	0.325	10.563	0.454	Serum
ApoB_LDL_4	NA	0.480	7.197	0.708	Serum
ApoB_LDL_5	0.952	0.012	7.619	0.476	Serum
ApoB_LDL_6	0.969	0.000	14.865	0.113	Serum
TG_HDL_1	0.994	0.006	4.093	0.246	Serum
TG_HDL_2	0.506	0.243	1.782	0.123	Serum
TG_HDL_3	0.231	0.116	1.878	0.086	Serum
TG_HDL_4	NA	0.005	2.360	-0.007	Serum
CHOL_HDL_1	0.960	0.015	26.651	0.582	Serum
CHOL_HDL_2	0.972	0.006	10.181	0.213	Serum
CHOL_HDL_3	NA	0.071	11.713	0.117	Serum
CHOL_HDL_4	0.944	0.012	18.956	-0.260	Serum
fCHOL_HDL_1	0.663	0.139	8.080	0.271	Serum
fCHOL_HDL_2	NA	0.197	3.147	0.110	Serum
fCHOL_HDL_3	0.515	0.166	3.200	0.107	Serum
fCHOL_HDL_4	0.952	0.003	4.313	0.061	Serum
PHOSL_HDL_1	NA	0.304	33.936	0.786	Serum
PHOSL_HDL_2	NA	0.157	16.047	0.282	Serum
PHOSL_HDL_3	NA	0.005	18.860	0.059	Serum
PHOSL_HDL_4	0.640	0.103	27.344	-0.340	Serum
ApoA1_HDL_1	0.379	0.375	47.390	1.376	Serum
ApoA1_HDL_2	NA	0.056	22.872	0.202	Serum
ApoA1_HDL_3	0.477	0.069	30.544	0.301	Serum
ApoA1_HDL_4	NA	0.019	72.660	-0.361	Serum
ApoA2_HDL_1	0.526	0.371	4.310	0.154	Serum
ApoA2_HDL_2	NA	0.288	4.205	0.119	Serum
ApoA2_HDL_3	0.505	0.155	6.871	0.149	Serum
ApoA2_HDL_4	NA	0.030	16.682	-0.098	Serum
pyr_lac	NA	0.092	0.058	-0.002	Serum
lac_glu	NA	0.092	0.321	0.016	Serum
alanine	0.889	0.055	0.405	0.019	LiHep
creatinine	0.197	0.086	0.095	0.004	LiHep
glutamine	NA	0.013	0.596	0.005	LiHep
glycine	NA	0.116	0.124	0.007	LiHep
histidine	NA	0.064	0.086	0.003	LiHep
isoleucine	0.934	0.005	0.060	0.002	LiHep
leucine	0.953	0.006	0.104	0.004	LiHep
lysine	0.957	0.007	0.184	0.007	LiHep
phenylalanine	0.850	0.005	0.044	0.001	LiHep
tyrosine	0.979	0.003	0.060	0.001	LiHep
valine	0.678	0.102	0.231	0.013	LiHep
citric_acid	NA	0.018	0.111	0.001	LiHep
lactic_acid	NA	0.449	1.334	0.163	LiHep
acetone	0.962	0.005	0.023	0.002	LiHep
pyruvic_acid	NA	0.094	0.080	0.003	LiHep
glucose	NA	0.183	4.889	0.123	LiHep
TG	NA	0.003	96.343	0.278	LiHep
CHOL	0.517	0.136	208.023	4.574	LiHep
LDL_CHOL	0.227	0.144	114.266	2.827	LiHep
HDL_CHOL	0.970	0.003	61.627	0.963	LiHep
ApoA1	NA	0.159	170.836	2.572	LiHep
ApoA2	0.928	0.015	34.072	0.642	LiHep
ApoB100	0.948	0.003	86.158	1.475	LiHep
LDL_CHOL_HDL_CHOL	0.933	0.002	1.748	0.020	LiHep
Apo.B100_ApoA1	0.930	0.000	0.499	0.001	LiHep
Total_Particles_ApoB	0.948	0.003	1566.493	26.822	LiHep

VLDL_Particles	NA	0.013	94.395	-0.561	LiHep
IDL_Particles	0.264	0.064	74.082	2.524	LiHep
LDL_Particles	0.421	0.039	1337.642	24.321	LiHep
LDL_1_Particles	0.073	0.010	204.354	1.178	LiHep
LDL_2_Particles	NA	0.010	198.870	1.194	LiHep
LDL_3_Particles	0.446	0.121	217.120	5.419	LiHep
LDL_4_Particles	0.771	0.031	234.786	7.467	LiHep
LDL_5_Particles	0.295	0.023	238.199	6.558	LiHep
LDL_6_Particles	NA	0.030	328.699	7.235	LiHep
TG_VLDL	NA	0.019	58.505	0.567	LiHep
TG_IDL	NA	0.004	5.728	0.045	LiHep
TG_LDL	NA	0.004	20.414	0.135	LiHep
TG_HDL	NA	0.000	12.382	0.013	LiHep
CHOL_VLDL	NA	0.023	14.510	0.223	LiHep
CHOL_IDL	NA	0.119	10.351	0.528	LiHep
fCHOL_VLDL	NA	0.001	7.388	0.013	LiHep
fCHOL_IDL	0.954	0.006	2.404	0.138	LiHep
fCHOL_LDL	0.456	0.084	41.119	0.873	LiHep
fCHOL_HDL	0.819	0.032	22.139	0.406	LiHep
PHOSL_VLDL	NA	0.007	14.978	-0.066	LiHep
PHOSL_IDL	0.967	0.007	5.098	0.265	LiHep
PHOSL_LDL	NA	0.125	63.703	1.231	LiHep
PHOSL_HDL	NA	0.089	88.562	1.162	LiHep
ApoA1_HDL	NA	0.114	172.514	2.227	LiHep
ApoA2_HDL	0.575	0.076	33.284	0.566	LiHep
ApoB_VLDL	NA	0.013	5.191	-0.031	LiHep
ApoB_IDL	0.184	0.070	4.081	0.139	LiHep
ApoB_LDL	0.421	0.039	73.573	1.338	LiHep
TG_VLDL_1	NA	0.001	28.619	-0.087	LiHep
TG_VLDL_2	NA	0.038	10.908	0.225	LiHep
TG_VLDL_3	0.971	0.001	7.908	0.201	LiHep
TG_VLDL_4	0.992	0.000	5.978	0.085	LiHep
TG_VLDL_5	0.675	0.003	2.700	-0.018	LiHep
CHOL_VLDL_1	NA	0.000	5.605	-0.011	LiHep
CHOL_VLDL_2	0.945	0.001	2.678	0.052	LiHep
CHOL_VLDL_3	NA	0.070	2.471	0.110	LiHep
CHOL_VLDL_4	NA	0.029	3.498	0.072	LiHep
CHOL_VLDL_5	NA	0.001	0.561	-0.003	LiHep
fCHOL_VLDL_1	NA	0.007	1.351	0.015	LiHep
fCHOL_VLDL_2	NA	0.030	0.775	0.025	LiHep
fCHOL_VLDL_3	NA	0.045	0.927	0.030	LiHep
fCHOL_VLDL_4	NA	0.148	1.659	0.080	LiHep
PHOSL_VLDL_1	NA	0.001	3.774	-0.014	LiHep
PHOSL_VLDL_2	NA	0.022	2.293	0.042	LiHep
PHOSL_VLDL_3	0.917	0.005	2.546	0.072	LiHep
PHOSL_VLDL_4	NA	0.071	2.904	0.055	LiHep
PHOSL_VLDL_5	0.899	0.005	1.338	-0.033	LiHep
TG_LDL_1	0.939	0.002	4.175	-0.075	LiHep
TG_LDL_2	NA	0.000	1.987	0.002	LiHep
TG_LDL_3	NA	0.010	2.386	-0.022	LiHep
TG_LDL_4	NA	0.004	3.070	0.029	LiHep
TG_LDL_5	NA	0.006	2.864	0.045	LiHep
TG_LDL_6	NA	0.014	4.250	0.060	LiHep
CHOL_LDL_1	0.044	0.028	19.257	0.217	LiHep
CHOL_LDL_2	0.368	0.010	18.221	0.163	LiHep
CHOL_LDL_3	0.357	0.180	19.713	0.600	LiHep
CHOL_LDL_4	0.852	0.031	18.625	0.676	LiHep
CHOL_LDL_5	0.593	0.019	18.314	0.573	LiHep

CHOL_LDL_6	0.879	0.005	21.238	0.551	LiHep
fCHOL_LDL_1	NA	0.092	7.072	0.140	LiHep
fCHOL_LDL_2	NA	0.047	7.285	0.104	LiHep
fCHOL_LDL_3	0.496	0.100	7.204	0.182	LiHep
fCHOL_LDL_4	0.912	0.023	6.681	0.221	LiHep
fCHOL_LDL_5	NA	0.048	6.364	0.167	LiHep
fCHOL_LDL_6	0.550	0.029	7.500	0.206	LiHep
PHOSL_LDL_1	0.412	0.006	11.021	0.065	LiHep
PHOSL_LDL_2	0.550	0.008	10.136	0.086	LiHep
PHOSL_LDL_3	0.328	0.172	10.995	0.281	LiHep
PHOSL_LDL_4	0.893	0.021	10.311	0.340	LiHep
PHOSL_LDL_5	0.530	0.018	10.199	0.269	LiHep
PHOSL_LDL_6	0.811	0.007	12.264	0.267	LiHep
ApoB_LDL_1	0.072	0.010	11.239	0.065	LiHep
ApoB_LDL_2	0.873	0.001	10.932	0.066	LiHep
ApoB_LDL_3	0.705	0.064	11.963	0.298	LiHep
ApoB_LDL_4	0.771	0.030	12.913	0.411	LiHep
ApoB_LDL_5	0.253	0.025	13.094	0.361	LiHep
ApoB_LDL_6	0.949	0.002	18.076	0.398	LiHep
TG_HDL_1	NA	0.002	4.416	0.025	LiHep
TG_HDL_2	NA	0.009	2.176	0.017	LiHep
TG_HDL_3	NA	0.000	2.465	-0.004	LiHep
TG_HDL_4	NA	0.116	3.129	-0.066	LiHep
CHOL_HDL_1	0.981	0.001	21.439	0.466	LiHep
CHOL_HDL_2	0.975	0.003	8.980	0.222	LiHep
CHOL_HDL_3	NA	0.217	11.439	0.257	LiHep
CHOL_HDL_4	0.386	0.019	17.480	0.143	LiHep
fCHOL_HDL_1	0.973	0.004	8.547	0.227	LiHep
fCHOL_HDL_2	NA	0.302	3.611	0.120	LiHep
fCHOL_HDL_3	NA	0.173	3.973	0.105	LiHep
fCHOL_HDL_4	0.961	0.004	5.410	0.109	LiHep
PHOSL_HDL_1	0.978	0.001	29.069	0.531	LiHep
PHOSL_HDL_2	0.974	0.003	14.418	0.273	LiHep
PHOSL_HDL_3	NA	0.186	18.304	0.290	LiHep
PHOSL_HDL_4	0.433	0.017	25.797	0.116	LiHep
ApoA1_HDL_1	0.811	0.005	44.259	0.673	LiHep
ApoA1_HDL_2	NA	0.031	20.502	0.232	LiHep
ApoA1_HDL_3	0.761	0.069	30.896	0.485	LiHep
ApoA1_HDL_4	0.870	0.014	73.137	0.598	LiHep
ApoA2_HDL_1	NA	0.027	3.981	0.086	LiHep
ApoA2_HDL_2	NA	0.116	4.138	0.108	LiHep
ApoA2_HDL_3	0.971	0.007	7.213	0.168	LiHep
ApoA2_HDL_4	0.754	0.018	16.662	0.197	LiHep
pyr_lac	NA	0.126	0.051	-0.002	LiHep
lac_glu	NA	0.273	0.271	0.029	LiHep

Table S6:Overview of metabolic parameters that change significantly (>20%) in the times categorized by the SPREC classification. Time-point were calculated from linear-mixed models in hours for the pre-centrifugation (left) and post-centrifugation (right) experiments.

Pre-Centrifugation				Post-Centrifugation			
**Metabolite**	**Type**	**Time**	**SPREC**	**Metabolite**	**Type**	**Time**	**SPREC**
lac_glu	EDTA	0.35	A1	lactic_acid	EDTA	0.82	B
lactic_acid	LiHep	0.41	A1	lac_glu	EDTA	0.82	B
lac_glu	LiHep	0.47	A1	lactic_acid	LiHep	1.64	D
lac_glu	Serum	0.54	A	TG_LDL_4	Serum	1.66	D
lactic_acid	EDTA	0.59	A	CHOL_IDL	Serum	1.72	D
glycine	LiHep	0.74	A	CHOL_VLDL_3	Serum	1.76	D
lactic_acid	Serum	0.76	A	fCHOL_IDL	Serum	1.83	D
pyruvic_acid	LiHep	1.64	A	lac_glu	LiHep	1.85	D
pyr_lac	Serum	1.90	A	ApoB_LDL_4	Serum	2.03	F
acetone	LiHep	2.14	C	LDL_4_Particles	Serum	2.03	F
pyr_lac	EDTA	2.16	C	TG_LDL_5	Serum	2.19	F
valine	LiHep	2.17	C	CHOL_LDL_4	Serum	2.25	F
CHOL_IDL	Serum	2.19	C	fCHOL_VLDL_2	Serum	2.29	F
fCHOL_VLDL_2	Serum	2.21	C	fCHOL_VLDL_4	Serum	2.31	F
CHOL_VLDL_3	Serum	2.21	C	fCHOL_IDL	EDTA	2.31	F
fCHOL_IDL	Serum	2.28	C	PHOSL_LDL_4	Serum	2.37	F
isoleucine	LiHep	2.35	C	glycine	EDTA	2.40	F
formic_acid	LiHep	2.45	C	acetone	LiHep	2.45	F
glycine	Serum	2.49	C	pyr_lac	EDTA	2.55	F
CHOL_LDL_5	Serum	2.54	C	CHOL_VLDL_3	EDTA	2.57	F
CHOL_VLDL_3	EDTA	2.57	C	IDL_Particles	Serum	2.59	F
fCHOL_VLDL_4	Serum	2.57	C	ApoB_IDL	Serum	2.59	F
LDL_5_Particles	Serum	2.58	C	fCHOL_VLDL_3	Serum	2.64	F
TG_LDL_5	Serum	2.58	C	CHOL_IDL	EDTA	2.70	F
ApoB_LDL_5	EDTA	2.58	C	fCHOL_VLDL_3	EDTA	2.72	F
LDL_5_Particles	EDTA	2.58	C	fCHOL_VLDL_2	EDTA	2.75	F
ApoB_LDL_5	Serum	2.58	C	PHOSL_IDL	EDTA	2.77	F
CHOL_LDL_5	EDTA	2.60	C	CHOL_VLDL_2	Serum	2.82	F
LDL_4_Particles	Serum	2.66	C	acetone	Serum	2.87	F
alanine	LiHep	2.66	C	TG_HDL_2	Serum	2.90	F
ApoB_LDL_4	Serum	2.68	C	PHOSL_VLDL_3	EDTA	2.98	F
fCHOL_IDL	EDTA	2.70	C	fCHOL_LDL_4	Serum	3.01	F
TG_LDL_5	EDTA	2.78	C	IDL_Particles	EDTA	3.11	F
glycine	EDTA	2.82	C	ApoB_IDL	EDTA	3.14	F
fCHOL_VLDL_2	EDTA	2.85	C	fCHOL_VLDL_4	EDTA	3.14	F
CHOL_LDL_4	Serum	2.86	C	CHOL_VLDL_5	EDTA	3.15	F
PHOSL_LDL_5	EDTA	2.88	C	CHOL_LDL_5	EDTA	3.20	F
CHOL_IDL	EDTA	2.92	C	LDL_5_Particles	Serum	3.20	F
pyr_lac	LiHep	2.98	C	ApoB_LDL_5	Serum	3.20	F
TG_LDL_4	EDTA	3.01	C	ApoB_LDL_5	EDTA	3.30	F
tyrosine	LiHep	3.03	C	LDL_5_Particles	EDTA	3.30	F
PHOSL_LDL_5	Serum	3.07	C	TG_HDL_1	Serum	3.33	F
creatinine	LiHep	3.10	C	CHOL_LDL_5	Serum	3.36	F
IDL_Particles	Serum	3.11	C	TG_LDL_5	EDTA	3.40	F
ApoB_IDL	Serum	3.14	C	fCHOL_IDL	LiHep	3.47	F
PHOSL_IDL	EDTA	3.14	C	glycine	LiHep	3.48	F
fCHOL_LDL_5	EDTA	3.15	C	valine	LiHep	3.58	F
PHOSL_IDL	LiHep	3.16	C	PHOSL_LDL_5	EDTA	3.64	F
PHOSL_LDL_4	Serum	3.16	C	PHOSL_IDL	Serum	3.67	F
fCHOL_VLDL_3	EDTA	3.17	C	LDL_4_Particles	EDTA	3.68	F

fCHOL_VLDL_3	Serum	3.28	C	ApoB_LDL_4	EDTA	3.69	F
IDL_Particles	EDTA	3.31	C	fCHOL_LDL_5	Serum	3.74	F
ApoB_IDL	EDTA	3.31	C	CHOL_LDL_4	EDTA	3.76	F
leucine	LiHep	3.39	C	PHOSL_IDL	LiHep	3.85	F
fCHOL_LDL_5	Serum	3.41	C	PHOSL_LDL_5	Serum	3.85	F
CHOL_VLDL_2	EDTA	3.43	C	CHOL_IDL	LiHep	3.92	F
PHOSL_IDL	Serum	3.48	C	lac_glu	Serum	3.95	F
TG_LDL_4	Serum	3.48	C	fCHOL_LDL_5	EDTA	4.00	F
PHOSL_VLDL_3	EDTA	3.60	C	fCHOL_VLDL_4	LiHep	4.13	F
fCHOL_LDL_4	Serum	3.62	C	CHOL_VLDL_4	Serum	4.18	F
ApoB_LDL_4	EDTA	3.67	C	TG_LDL	Serum	4.19	F
LDL_4_Particles	EDTA	3.67	C	TG_LDL_4	EDTA	4.20	F
fCHOL_VLDL_4	EDTA	3.77	C	PHOSL_LDL_4	EDTA	4.29	F
CHOL_VLDL_5	EDTA	3.80	C	CHOL_VLDL_2	EDTA	4.30	F
CHOL_LDL_4	EDTA	3.94	C	lactic_acid	Serum	4.32	F
lysine	LiHep	3.96	C	alanine	LiHep	4.34	F
CHOL_VLDL_2	Serum	3.96	C	TG_HDL_3	Serum	4.36	F
histidine	Serum	4.03	E	TG_HDL	Serum	4.41	F
histidine	LiHep	4.09	E	CHOL_VLDL_3	LiHep	4.49	F
PHOSL_VLDL_3	Serum	4.10	E	fCHOL_LDL_4	EDTA	4.52	F
fCHOL_IDL	LiHep	4.12	E	TG_HDL_1	EDTA	4.53	F
fCHOL_VLDL_4	LiHep	4.15	E	PHOSL_VLDL_3	Serum	4.65	F
phenylalanine	Serum	4.18	E	ApoB_LDL_3	Serum	4.66	F
glutamine	LiHep	4.21	E	LDL_3_Particles	Serum	4.66	F
TG_HDL_2	Serum	4.25	E	TG_VLDL_3	EDTA	4.88	F
PHOSL_LDL_4	EDTA	4.43	E	CHOL_LDL_3	Serum	4.90	F
acetone	Serum	4.44	E	CHOL_LDL_6	EDTA	5.08	F
CHOL_VLDL_5	Serum	4.44	E	TG_HDL_2	EDTA	5.13	F
TG_HDL_1	Serum	4.54	E	isoleucine	LiHep	5.22	F
fCHOL_LDL_4	EDTA	4.56	E	PHOSL_LDL_3	Serum	5.31	F
leucine	Serum	4.60	E	CHOL_VLDL	Serum	5.32	F
TG_VLDL_3	EDTA	4.63	E	PHOSL_VLDL_5	EDTA	5.32	F
CHOL_VLDL_5	LiHep	4.68	E	creatinine	LiHep	5.34	F
PHOSL_VLDL_5	EDTA	4.71	E	LDL_6_Particles	EDTA	5.40	F
CHOL_IDL	LiHep	4.73	E	ApoB_LDL_6	EDTA	5.40	F
CHOL_LDL_6	EDTA	4.73	E	leucine	LiHep	5.49	F
TG_HDL_2	EDTA	4.75	E	CHOL_LDL_4	LiHep	5.51	F
fCHOL_LDL_6	EDTA	4.76	E	PHOSL_VLDL_2	EDTA	5.52	F
TG_LDL	EDTA	4.78	E	CHOL_VLDL_4	EDTA	5.54	F
ApoB_LDL_6	EDTA	4.79	E	lysine	LiHep	5.57	F
LDL_6_Particles	EDTA	4.79	E	TG_VLDL_3	Serum	5.57	F
fCHOL_HDL_2	Serum	5.07	E	ApoA2_HDL_1	Serum	5.59	F
CHOL_HDL_1	LiHep	5.07	E	TG_LDL_2	Serum	5.67	F
CHOL_VLDL_4	EDTA	5.17	E	fCHOL_HDL_2	Serum	5.70	F
PHOSL_VLDL_2	EDTA	5.26	E	ApoA2_HDL_1	EDTA	5.86	F
ApoA2_HDL_2	LiHep	5.26	E	IDL_Particles	LiHep	5.87	F
CHOL_LDL_4	LiHep	5.36	E	ApoB_IDL	LiHep	5.89	F
ApoA2_HDL_1	LiHep	5.45	E	pyruvic_acid	LiHep	5.89	F
TG_HDL_3	EDTA	5.55	E	fCHOL_LDL_3	Serum	5.91	F
CHOL_VLDL_4	Serum	5.67	E	fCHOL_LDL_6	EDTA	5.93	F
TG_VLDL_2	EDTA	5.68	E	fCHOL_HDL_3	Serum	5.96	F
TG_HDL_1	EDTA	5.70	E	fCHOL_HDL_1	Serum	5.97	F
ApoA2_HDL_2	Serum	5.76	E	fCHOL_HDL_2	LiHep	6.00	F
LDL_CHOL	EDTA	5.79	E	fCHOL_LDL_4	LiHep	6.05	F
alanine	Serum	5.83	E	PHOSL_LDL_4	LiHep	6.06	F
ApoA2_HDL_1	Serum	5.85	E	fCHOL_VLDL_3	LiHep	6.13	F
PHOSL_LDL_4	LiHep	5.86	E	fCHOL_VLDL_2	LiHep	6.18	F
CHOL_HDL_2	LiHep	5.93	E	pyr_lac	LiHep	6.22	F

Total_Particles_ApoB	EDTA	5.94	E
ApoB100	EDTA	5.94	E
ApoB_LDL	EDTA	5.94	E
LDL_Particles	EDTA	5.94	E
fCHOL_HDL_2	LiHep	5.94	E
PHOSL_LDL_6	EDTA	5.98	E
TG_LDL_1	LiHep	5.99	E
CHOL_LDL_3	LiHep	6.03	E
ApoB_IDL	LiHep	6.04	E
IDL_Particles	LiHep	6.04	E
PHOSL_HDL_1	LiHep	6.07	E
fCHOL_HDL_1	Serum	6.11	E
glucose	EDTA	6.16	E
TG_VLDL_3	Serum	6.20	E
fCHOL_HDL_2	EDTA	6.24	E
PHOSL_VLDL_3	LiHep	6.26	E
fCHOL_HDL_3	EDTA	6.34	E
CHOL_HDL_2	Serum	6.35	E
CHOL_LDL_6	Serum	6.40	E
TG_LDL	Serum	6.43	E
LDL_4_Particles	LiHep	6.47	E
ApoB_LDL_4	LiHep	6.47	E
CHOL_VLDL	EDTA	6.71	E
TG_HDL	Serum	6.78	E
CHOL_VLDL_3	LiHep	6.79	E
fCHOL_HDL_1	LiHep	6.81	E
lysine	Serum	6.83	E
fCHOL_LDL	EDTA	6.83	E
fCHOL_VLDL_1	LiHep	6.83	E
fCHOL_HDL_1	EDTA	6.88	E
CHOL_VLDL	Serum	6.90	E
PHOSL_VLDL_4	Serum	6.91	E
PHOSL_VLDL_5	Serum	6.94	E
TG_HDL	EDTA	6.96	E
ApoB_LDL_3	EDTA	7.02	E
LDL_3_Particles	EDTA	7.02	E
CHOL_LDL_3	Serum	7.05	E
fCHOL_LDL_3	Serum	7.08	E
PHOSL_HDL_2	LiHep	7.13	E
PHOSL_LDL	EDTA	7.16	E
LDL_CHOL	Serum	7.19	E
CHOL_HDL_3	LiHep	7.25	E
TG_HDL_4	LiHep	7.26	E
ApoB_LDL_6	Serum	7.26	E
fCHOL_HDL_3	Serum	7.31	E
ApoA2_HDL_3	LiHep	7.35	E
LDL_3_Particles	Serum	7.35	E
ApoB_LDL_3	Serum	7.36	E
ApoA2_HDL_3	Serum	7.36	E
LDL_6_Particles	Serum	7.38	E
fCHOL_VLDL_3	LiHep	7.39	E
ApoA2_HDL_1	EDTA	7.41	E
fCHOL_LDL_4	LiHep	7.41	E
ApoA1_HDL_1	Serum	7.41	E
PHOSL_LDL_3	LiHep	7.47	E
TG_VLDL_5	EDTA	7.50	E
fCHOL_VLDL_2	LiHep	7.55	E
fCHOL_LDL_3	EDTA	7.56	E
fCHOL_LDL_1	Serum	6.28	F
LDL_4_Particles	LiHep	6.29	F
ApoB_LDL_4	LiHep	6.29	F
ApoA2_HDL_2	EDTA	6.30	F
histidine	LiHep	6.32	F
isoleucine	Serum	6.33	F
PHOSL_LDL_6	EDTA	6.33	F
TG_LDL	EDTA	6.33	F
fCHOL_HDL_2	EDTA	6.36	F
fCHOL_VLDL_1	EDTA	6.39	F
CHOL_LDL_5	LiHep	6.40	F
LDL_CHOL	EDTA	6.42	F
CHOL_LDL_3	LiHep	6.57	F
PHOSL_VLDL_4	EDTA	6.63	F
creatinine	Serum	6.73	F
pyr_lac	Serum	6.79	F
ApoA1_HDL_1	Serum	6.89	F
fCHOL_HDL_1	EDTA	6.90	F
CHOL_VLDL_5	Serum	6.93	F
histidine	Serum	6.93	F
PHOSL_VLDL_2	Serum	7.00	F
ApoA2_HDL_2	Serum	7.05	F
CHOL_VLDL	EDTA	7.06	F
Total_Particles_ApoB	EDTA	7.08	F
ApoB100	EDTA	7.08	F
PHOSL_VLDL_3	LiHep	7.10	F
ApoB_LDL	EDTA	7.22	F
LDL_Particles	EDTA	7.22	F
ApoB_LDL_5	LiHep	7.26	F
LDL_5_Particles	LiHep	7.26	F
TG_VLDL_2	EDTA	7.28	F
fCHOL_LDL_6	LiHep	7.28	F
phenylalanine	Serum	7.29	F
TG_LDL_1	Serum	7.29	F
fCHOL_HDL_3	EDTA	7.35	F
Total_Particles_ApoB	Serum	7.38	F
ApoB100	Serum	7.38	F
fCHOL_LDL	Serum	7.40	F
ApoA2_HDL_3	EDTA	7.40	F
PHOSL_VLDL_4	Serum	7.48	F
fCHOL_HDL_1	LiHep	7.52	F
fCHOL_HDL_3	LiHep	7.56	F
TG_HDL_3	EDTA	7.57	F
valine	EDTA	7.59	F
PHOSL_LDL_5	LiHep	7.59	F
fCHOL_LDL_5	LiHep	7.63	F
TG_HDL	EDTA	7.65	F
fCHOL_HDL	Serum	7.66	F
ApoA2_HDL_2	LiHep	7.69	F
CHOL_LDL_6	LiHep	7.71	F
CHOL_VLDL_1	Serum	7.77	F
PHOSL_LDL_3	LiHep	7.83	F
TG_VLDL_3	LiHep	7.88	F
CHOL_LDL_3	EDTA	7.89	F
ApoA1_HDL_1	EDTA	7.89	F
fCHOL_LDL_3	LiHep	7.93	F
citric_acid	EDTA	7.93	F
glucose	LiHep	7.97	F

LDL_CHOL_HDL_CHOL	EDTA	7.58	E	PHOSL_VLDL_5	LiHep	7.99	F
fCHOL_LDL_1	LiHep	7.62	E	fCHOL_LDL	EDTA	8.00	H
ApoA1_HDL_1	EDTA	7.68	E	LDL_3_Particles	LiHep	8.01	H
fCHOL_LDL_6	Serum	7.71	E	ApoA2	EDTA	8.03	H
CHOL_LDL_1	LiHep	7.74	E	ApoB_LDL_3	LiHep	8.03	H
phenylalanine	LiHep	7.82	E	CHOL	EDTA	8.05	H
ApoB100	Serum	7.83	E	LDL_CHOL	LiHep	8.08	H
Total_Particles_ApoB	Serum	7.83	E	CHOL_HDL_2	LiHep	8.09	H
PHOSL_HDL_2	Serum	7.84	E	TG_VLDL_2	Serum	8.10	H
ApoA2_HDL_2	EDTA	7.84	E	fCHOL_LDL_3	EDTA	8.11	H
leucine	EDTA	7.93	E	acetone	EDTA	8.18	H
tyrosine	Serum	7.94	E	PHOSL_LDL	EDTA	8.44	H
PHOSL_VLDL_2	Serum	7.96	E	ApoB_LDL_3	EDTA	8.52	H
CHOL	LiHep	8.03	G	LDL_3_Particles	EDTA	8.53	H
fCHOL_LDL_2	EDTA	8.03	G	ApoA2_HDL	EDTA	8.59	H
CHOL	EDTA	8.05	G	LDL_CHOL	Serum	8.60	H
PHOSL_LDL_3	Serum	8.10	G	ApoA2_HDL_3	LiHep	8.60	H
alanine	EDTA	8.15	G	CHOL_LDL_1	Serum	8.63	H
LDL_Particles	Serum	8.17	G	PHOSL_HDL_1	Serum	8.63	H
ApoB_LDL	Serum	8.17	G	LDL_1_Particles	Serum	8.77	H
LDL_3_Particles	LiHep	8.20	G	ApoB_LDL_1	Serum	8.78	H
ApoB_LDL_3	LiHep	8.20	G	tyrosine	LiHep	8.78	H
CHOL	Serum	8.24	G	fCHOL_LDL_2	Serum	8.80	H
pyruvic_acid	EDTA	8.25	G	CHOL_HDL_2	EDTA	8.88	H
fCHOL_LDL	Serum	8.27	G	CHOL_HDL_3	LiHep	8.89	H
isoleucine	Serum	8.36	G	TG_IDL	EDTA	8.91	H
PHOSL_VLDL_4	EDTA	8.39	G	ApoB_LDL	Serum	9.06	H
TG_HDL_3	Serum	8.41	G	LDL_Particles	Serum	9.06	H
PHOSL_LDL_6	Serum	8.44	G	ApoB_LDL_6	LiHep	9.08	H
PHOSL_VLDL_4	LiHep	8.51	G	LDL_6_Particles	LiHep	9.09	H
CHOL_HDL_1	Serum	8.74	G	CHOL	LiHep	9.10	H
LDL_CHOL	LiHep	8.80	G	TG_VLDL_5	EDTA	9.11	H
PHOSL_HDL_1	Serum	8.80	G	CHOL_HDL_1	Serum	9.15	H
valine	Serum	8.81	G	TG_VLDL	EDTA	9.18	H
HDL_CHOL	LiHep	8.85	G	PHOSL_LDL_6	LiHep	9.18	H
ApoA2	LiHep	8.85	G	CHOL	Serum	9.19	H
TG_LDL_2	EDTA	8.88	G	CHOL_HDL_1	LiHep	9.21	H
ApoA2_HDL_3	EDTA	8.89	G	ApoA2_HDL_3	Serum	9.23	H
CHOL_LDL_3	EDTA	8.90	G	ApoA2_HDL_1	LiHep	9.26	H
ApoA2	Serum	8.94	G	fCHOL_LDL	LiHep	9.42	H
TG_IDL	EDTA	8.98	G	TG_HDL_4	LiHep	9.48	H
TG_LDL_6	EDTA	8.98	G	TG_VLDL_4	EDTA	9.48	H
pyruvic_acid	Serum	9.03	G	CHOL_HDL_2	Serum	9.56	H
fCHOL_VLDL_1	EDTA	9.05	G	PHOSL_LDL_3	EDTA	9.65	H
PHOSL_LDL	Serum	9.37	G	TG_VLDL_2	LiHep	9.68	H
ApoA2_HDL	Serum	9.52	G	CHOL_VLDL_4	LiHep	9.69	H
valine	EDTA	9.60	G	TG_LDL_6	EDTA	9.73	H
glucose	Serum	9.70	G	PHOSL_LDL_1	Serum	9.85	H
ApoA1_HDL_1	LiHep	9.74	G	fCHOL_HDL_4	LiHep	9.90	H
fCHOL_LDL_3	LiHep	9.75	G	fCHOL_LDL_6	Serum	10.01	H
PHOSL_HDL_3	LiHep	9.82	G	CHOL_HDL_1	EDTA	10.03	H
ApoA2_HDL	LiHep	9.84	G	alanine	EDTA	10.08	H
PHOSL_LDL_3	EDTA	9.84	G	fCHOL_LDL_1	LiHep	10.13	H
PHOSL_HDL	LiHep	9.88	G	CHOL_VLDL_2	LiHep	10.24	H
ApoA1_HDL_2	LiHep	9.92	G	lysine	EDTA	10.33	H
CHOL_HDL_3	Serum	9.93	G	PHOSL_LDL	LiHep	10.35	H
PHOSL_VLDL_5	LiHep	9.94	G	PHOSL_VLDL_4	LiHep	10.54	H
ApoA2	EDTA	9.97	G	LDL_CHOL_HDL_CHOL	EDTA	10.58	H

TG_LDL_2	Serum	10.15	G
fCHOL_HDL_4	EDTA	10.32	G
ApoA1_HDL_3	LiHep	10.32	G
creatinine	Serum	10.42	G
TG_VLDL	EDTA	10.44	G
CHOL_LDL_5	LiHep	10.54	G
fCHOL_HDL	Serum	10.84	G
fCHOL_LDL_1	EDTA	10.84	G
ApoA2_HDL	EDTA	10.90	G
ApoA1	Serum	10.97	G
PHOSL_LDL	LiHep	11.03	G
CHOL_LDL_6	LiHep	11.09	G
fCHOL_HDL_3	LiHep	11.17	G
TG_VLDL_4	Serum	11.24	G
ApoA1	LiHep	11.30	G
citric_acid	Serum	11.37	G
PHOSL_LDL_1	LiHep	11.47	G
acetone	EDTA	11.49	G
TG_VLDL_2	Serum	11.72	G
TG_LDL_6	Serum	11.74	G
ApoA1_HDL_2	Serum	11.74	G
fCHOL_HDL	EDTA	11.83	G
fCHOL_LDL	LiHep	11.93	G
CHOL_VLDL_1	Serum	11.96	G
CHOL_VLDL	LiHep	12.01	I
ApoA1_HDL	EDTA	12.10	I
TG_VLDL	Serum	12.13	I
citric_acid	EDTA	12.16	I
fCHOL_HDL	LiHep	12.20	I
CHOL_VLDL_1	EDTA	12.20	I
ApoA1_HDL	Serum	12.23	I
CHOL_VLDL_4	LiHep	12.25	I
PHOSL_LDL_6	LiHep	12.25	I
ApoA1_HDL_3	Serum	12.30	I
ApoA1	EDTA	12.33	I
HDL_CHOL	Serum	12.45	I
Apo.B100_ApoA1	EDTA	13.07	I
CHOL_HDL_2	EDTA	13.10	I
ApoA2_HDL_4	LiHep	13.19	I
isoleucine	EDTA	13.61	I
LDL_2_Particles	EDTA	13.82	I
ApoB_LDL_2	EDTA	13.83	I
PHOSL_LDL_5	LiHep	13.86	I
TG_VLDL_3	LiHep	13.91	I
TG_VLDL_5	Serum	14.14	I
TG_HDL_4	Serum	14.21	I
TG_VLDL_4	LiHep	14.22	I
ApoA1_HDL	LiHep	14.31	I
TG_LDL_3	LiHep	14.54	I
fCHOL_LDL_1	Serum	14.56	I
TG	EDTA	14.59	I
LDL_5_Particles	LiHep	15.12	I
ApoB_LDL_5	LiHep	15.12	I
fCHOL_VLDL	EDTA	15.13	I
PHOSL_HDL	Serum	15.30	I
PHOSL_HDL_1	EDTA	15.36	I
TG_HDL_3	LiHep	15.53	I
PHOSL_VLDL_2	LiHep	15.63	I

PHOSL_HDL_2	LiHep	10.58	H
ApoA2	LiHep	10.62	H
PHOSL_LDL	Serum	10.78	H
ApoA1	EDTA	10.90	H
leucine	EDTA	10.90	H
fCHOL_HDL	LiHep	10.91	H
PHOSL_HDL_1	LiHep	10.95	H
LDL_Particles	LiHep	11.00	H
ApoB_LDL	LiHep	11.00	H
PHOSL_VLDL_2	LiHep	11.03	H
TG_LDL_1	LiHep	11.10	H
PHOSL_HDL_1	EDTA	11.25	H
valine	Serum	11.29	H
PHOSL_HDL_2	Serum	11.36	H
glycine	Serum	11.43	H
ApoA1_HDL	EDTA	11.67	H
Total_Particles_ApoB	LiHep	11.68	H
ApoB100	LiHep	11.68	H
alanine	Serum	11.70	H
tyrosine	Serum	11.74	H
ApoA2_HDL	LiHep	11.76	H
TG_LDL_2	EDTA	11.88	H
CHOL_HDL_3	EDTA	12.13	H
fCHOL_HDL_4	EDTA	12.31	H
PHOSL_HDL_3	LiHep	12.61	H
PHOSL_HDL_2	EDTA	12.72	H
ApoA1_HDL_3	LiHep	12.74	H
TG_LDL_5	LiHep	12.76	H
HDL_CHOL	LiHep	12.79	H
CHOL_VLDL_1	EDTA	12.88	H
CHOL_VLDL	LiHep	13.04	H
fCHOL_LDL_1	EDTA	13.11	H
ApoA1_HDL_1	LiHep	13.16	H
leucine	Serum	13.23	H
pyruvic_acid	EDTA	13.26	H
ApoA1	LiHep	13.28	H
ApoA2	Serum	13.65	H
fCHOL_HDL	EDTA	13.79	H
fCHOL_LDL_2	EDTA	13.85	H
fCHOL_LDL_2	LiHep	13.97	H
ApoA2_HDL_4	EDTA	14.02	H
TG_LDL_6	LiHep	14.05	H
fCHOL_HDL_4	Serum	14.06	H
TG_VLDL_4	LiHep	14.10	H
Apo.B100_ApoA1	Serum	14.31	H
tyrosine	EDTA	14.40	H
HDL_CHOL	EDTA	14.46	H
ApoB_LDL_2	Serum	14.50	H
LDL_CHOL_HDL_CHOL	Serum	14.50	H
phenylalanine	LiHep	14.50	H
LDL_2_Particles	Serum	14.52	H
CHOL_HDL_4	Serum	14.57	H
CHOL_LDL_2	Serum	14.61	H
fCHOL_VLDL	EDTA	15.04	H
ApoA1	Serum	15.18	H
PHOSL_HDL	LiHep	15.25	H
TG	EDTA	15.36	H
TG	Serum	15.37	H

TG_IDL	Serum	15.89	I	ApoA1_HDL	LiHep	15.49	H
LDL_Particles	LiHep	16.10	I	ApoA2_HDL	Serum	15.50	H
ApoB_LDL	LiHep	16.11	I	citric_acid	LiHep	15.95	H
PHOSL_HDL_3	Serum	16.12	I	PHOSL_HDL_4	Serum	16.07	H
CHOL_LDL_2	EDTA	16.12	I	fCHOL_VLDL	Serum	16.08	H
CHOL_HDL_4	LiHep	16.50	I	TG_LDL_3	Serum	16.15	H
ApoB_LDL_1	LiHep	16.86	I	isoleucine	EDTA	16.25	H
LDL_1_Particles	LiHep	16.88	I	ApoA1_HDL_2	EDTA	16.65	H
PHOSL_HDL_2	EDTA	16.94	I	ApoA1_HDL_3	EDTA	16.65	H
citric_acid	LiHep	16.94	I	VLDL_Particles	EDTA	16.87	H
TG_IDL	LiHep	17.31	I	ApoB_VLDL	EDTA	16.88	H
fCHOL_LDL_2	Serum	17.71	I	ApoA2_HDL_4	LiHep	16.90	H
LDL_CHOL_HDL_CHOL	Serum	17.77	I	ApoA1_HDL	Serum	17.27	H
CHOL_HDL_1	EDTA	18.29	I	ApoA1_HDL_2	LiHep	17.64	H
fCHOL_LDL_6	LiHep	18.38	I	CHOL_LDL_1	LiHep	17.73	H
TG_LDL_1	Serum	18.72	I	LDL_CHOL_HDL_CHOL	LiHep	17.74	H
CHOL_HDL_3	EDTA	18.84	I	glutamine	Serum	17.97	H
ApoB_LDL_6	LiHep	18.95	I	fCHOL_VLDL_1	LiHep	18.14	H
LDL_6_Particles	LiHep	18.97	I	glucose	Serum	18.84	H
TG_VLDL_4	EDTA	18.99	I	citric_acid	Serum	18.90	H
fCHOL_VLDL_1	Serum	19.16	I	TG_VLDL_1	EDTA	19.26	H
TG	Serum	19.80	I	lysine	Serum	19.51	H
fCHOL_LDL_5	LiHep	20.11	I	ApoA1_HDL_4	EDTA	19.65	H
glutamine	EDTA	20.72	I	PHOSL_LDL_2	Serum	19.73	H
TG_VLDL	LiHep	20.73	I	CHOL_HDL_3	Serum	20.01	H
TG_HDL_4	EDTA	21.01	I	PHOSL_HDL	EDTA	20.06	H
ApoB100	LiHep	21.07	I	ApoA1_HDL_3	Serum	20.29	H
Total_Particles_ApoB	LiHep	21.07	I	TG_VLDL	LiHep	20.65	H
ApoA1_HDL_2	EDTA	21.33	I	TG_LDL_4	LiHep	21.21	H
lysine	EDTA	21.46	I	VLDL_Particles	Serum	21.26	H
TG_LDL_1	EDTA	21.52	I	ApoB_VLDL	Serum	21.26	H
PHOSL_VLDL_1	LiHep	21.58	I	PHOSL_HDL_3	EDTA	21.41	H
HDL_CHOL	EDTA	21.85	I	PHOSL_VLDL_1	EDTA	21.75	H
fCHOL_VLDL	Serum	21.96	I	TG_LDL_3	LiHep	22.09	H
PHOSL_HDL_4	EDTA	22.40	I	Apo.B100_ApoA1	EDTA	22.13	H
ApoA1_HDL_3	EDTA	22.53	I	CHOL_LDL_2	LiHep	22.31	H
TG_VLDL_2	LiHep	22.62	I	ApoA1_HDL_2	Serum	22.66	H
phenylalanine	EDTA	23.00	I	creatinine	EDTA	22.95	H
ApoA1_HDL_4	EDTA	23.01	I	TG_LDL_6	Serum	23.09	H
TG_LDL	LiHep	23.63	I	glutamine	LiHep	23.15	H
VLDL_Particles	EDTA	24.88	K	PHOSL_LDL_2	LiHep	23.52	H
ApoB_VLDL	EDTA	24.91	K	CHOL_LDL_1	EDTA	23.70	H
TG_HDL_1	LiHep	24.94	K	CHOL_HDL_4	LiHep	24.40	J
PHOSL_HDL	EDTA	24.99	K	TG_IDL	Serum	24.47	J
PHOSL_HDL_3	EDTA	25.04	K	ApoA1_HDL_4	LiHep	24.47	J
tyrosine	EDTA	25.06	K	TG_VLDL	Serum	24.66	J
LDL_1_Particles	EDTA	25.38	K	TG_HDL_2	LiHep	25.40	J
ApoB_LDL_1	EDTA	25.39	K	TG_IDL	LiHep	25.52	J
PHOSL_HDL_4	Serum	25.73	K	CHOL_LDL_6	Serum	25.64	J
ApoB_VLDL	Serum	25.97	K	ApoB_LDL_6	Serum	26.28	J
VLDL_Particles	Serum	25.98	K	LDL_6_Particles	Serum	26.29	J
CHOL_LDL_1	Serum	26.50	K	PHOSL_HDL	Serum	26.66	J
CHOL_VLDL_2	LiHep	26.69	K	HDL_CHOL	Serum	26.73	J
CHOL_LDL_1	EDTA	26.92	K	glutamine	EDTA	27.35	J
ApoA1_HDL_4	LiHep	27.56	K	PHOSL_VLDL	EDTA	27.88	J
PHOSL_HDL_4	LiHep	28.15	K	pyruvic_acid	Serum	28.00	J
CHOL_HDL_4	EDTA	28.61	K	phenylalanine	EDTA	28.96	J
TG_LDL_6	LiHep	30.12	K	TG_LDL	LiHep	30.35	J

PHOSL_LDL_2	EDTA	31.85	K	TG_VLDL_5	LiHep	30.44	J
fCHOL_HDL_4	Serum	32.94	K	TG_VLDL_4	Serum	32.25	J
Apo.B100_ApoA1	LiHep	33.11	K	CHOL_VLDL_5	LiHep	32.52	J
TG_VLDL_1	Serum	33.28	K	TG_LDL_3	EDTA	32.95	J
fCHOL_HDL_4	LiHep	33.69	K	ApoB_LDL_2	LiHep	33.22	J
Apo.B100_ApoA1	Serum	33.96	K	LDL_2_Particles	LiHep	33.31	J
formic_acid	Serum	35.05	K	VLDL_Particles	LiHep	33.67	J
ApoA2_HDL_4	Serum	37.52	K	PHOSL_LDL_1	LiHep	33.76	J
fCHOL_LDL_2	LiHep	38.94	K	ApoB_VLDL	LiHep	33.81	J
PHOSL_VLDL	EDTA	40.27	K	ApoA2_HDL_4	Serum	34.13	J
CHOL_LDL_2	LiHep	41.47	K	ApoB_LDL_1	LiHep	34.68	J
TG_LDL_3	Serum	42.13	K	LDL_1_Particles	LiHep	34.70	J
TG_LDL_4	LiHep	42.75	K	TG_HDL_1	LiHep	35.47	J
ApoB_LDL_1	Serum	43.46	K	CHOL_LDL_2	EDTA	36.56	J
LDL_1_Particles	Serum	43.48	K	fCHOL_VLDL_1	Serum	37.34	J
PHOSL_VLDL	LiHep	45.14	K	PHOSL_VLDL_5	Serum	39.02	J
TG_LDL_2	LiHep	47.15	K	ApoA1_HDL_4	Serum	40.20	J
LDL_2_Particles	LiHep	47.23	K	PHOSL_HDL_4	LiHep	44.39	J
ApoB_LDL_2	LiHep	47.23	K	PHOSL_VLDL	LiHep	45.19	J
TG_VLDL_5	LiHep	47.43	K				
ApoA2_HDL_4	EDTA	47.69	K				

Table S7: Estimated mean percentage error values per time point for every parameter for pre-centrifugation and for post-centrifugation models.

ID	Time	EDTA		Serum		LiHep	
		Pre-	Post-	Pre-	Post-	Pre-	Post-
alanine	2	0.87	2.01	4.84	1.91	12.45	7.56
alanine	4	2.73	6.62	5.00	5.16	15.57	15.70
alanine	6	3.41	8.95	9.35	6.79	22.48	21.31
alanine	8	5.49	12.19	11.88	10.60	28.64	25.52
creatinine	2	-5.03	-1.45	4.04	1.17	1.14	5.36
creatinine	4	-13.96	2.21	16.37	-0.31	12.17	14.60
creatinine	6	-21.37	4.08	12.49	12.56	10.25	14.12
creatinine	8	-24.57	10.75	5.13	17.33	17.17	20.43
glutamine	2	-3.36	-2.88	0.13	1.40	2.04	3.34
glutamine	4	18.21	-0.99	6.82	5.96	23.45	3.55
glutamine	6	11.47	1.29	10.52	16.29	30.89	6.11
glutamine	8	29.68	4.36	17.56	22.43	37.41	7.76
glycine	2	-13.01	8.57	0.54	3.06	8.47	11.52
glycine	4	27.01	21.91	-3.23	15.23	20.90	21.23
glycine	6	17.11	22.88	8.26	0.76	38.00	24.43
glycine	8	31.40	25.72	12.42	21.08	34.29	20.07
histidine	2	-1.82	9.07	6.12	22.32	-3.49	8.78
histidine	4	3.09	8.15	10.37	-10.38	10.56	7.41
histidine	6	-5.45	10.94	8.21	22.71	23.67	4.86
histidine	8	11.29	10.02	12.28	25.81	29.53	4.98
isoleucine	2	1.01	7.38	-0.61	5.61	17.07	4.52
isoleucine	4	-1.73	19.46	2.32	3.76	25.32	20.49
isoleucine	6	13.66	8.87	0.00	8.61	28.37	14.13
isoleucine	8	1.97	15.60	7.45	0.58	35.45	23.41
leucine	2	9.17	1.77	10.49	2.96	7.95	8.34
leucine	4	4.05	9.11	2.90	5.76	12.80	12.80
leucine	6	13.49	14.63	10.34	-0.95	19.17	18.04
leucine	8	14.40	18.48	11.41	10.89	22.44	16.45
lysine	2	-19.98	-5.18	0.64	4.11	-1.78	20.53
lysine	4	-5.17	10.17	11.53	6.32	7.63	11.53
lysine	6	-13.64	12.79	13.58	18.84	17.58	21.33
lysine	8	-15.92	13.25	12.37	12.08	17.47	27.55
phenylalanine	2	0.37	-12.45	-8.22	-16.36	-5.13	19.36
phenylalanine	4	6.73	3.17	-11.89	2.91	0.20	11.46
phenylalanine	6	4.47	1.96	-2.33	-3.86	-2.35	4.05
phenylalanine	8	13.56	-7.26	1.36	-17.52	7.25	6.06
tyrosine	2	3.86	8.98	3.59	-0.47	11.70	5.33
tyrosine	4	9.64	8.87	10.39	7.50	19.68	10.13
tyrosine	6	9.17	11.54	11.41	6.78	24.86	14.46
tyrosine	8	8.71	14.05	13.96	10.03	26.53	15.58
valine	2	-0.64	4.74	6.54	3.33	12.10	12.38
valine	4	4.89	12.40	6.88	5.64	24.05	15.85
valine	6	5.47	18.86	7.54	13.31	25.37	24.67
valine	8	9.91	15.58	12.64	17.47	31.28	27.69
citric_acid	2	5.73	-6.60	-23.79	3.63	-4.05	16.47
citric_acid	4	8.82	11.06	-25.95	14.37	-3.04	27.06
citric_acid	6	9.53	15.51	-14.29	5.32	8.66	6.44
citric_acid	8	10.90	16.85	-63.64	42.28	12.10	5.74
lactic_acid	2	-10.68	19.91	-8.96	0.11	-4.87	10.24
lactic_acid	4	-17.49	5.01	-8.42	7.95	-18.53	19.61
lactic_acid	6	-19.68	16.44	0.75	11.74	-22.08	20.87
lactic_acid	8	-17.84	5.64	3.66	18.28	-25.81	-7.26

acetone	2	-9.57	-54.13	-6.47	-4.26	-33.01	15.95
acetone	4	15.99	-11.87	18.58	19.52	26.25	5.77
acetone	6	5.19	-41.74	13.63	-1.76	10.12	-1.94
acetone	8	6.88	-17.65	7.73	44.32	-3.11	19.48
pyruvic_acid	2	-14.56	-4.75	8.18	2.88	21.77	4.56
pyruvic_acid	4	-15.85	-7.87	-0.71	-3.20	34.72	6.04
pyruvic_acid	6	-15.94	-4.00	-14.39	1.41	30.88	6.11
pyruvic_acid	8	-35.62	-2.98	-11.35	4.19	32.50	7.41
glucose	2	2.55	1.42	4.79	0.17	9.24	3.90
glucose	4	17.19	7.13	11.67	3.15	24.85	9.40
glucose	6	26.88	9.24	13.85	4.45	37.36	14.74
glucose	8	33.58	17.57	18.51	5.70	48.27	23.72
TG	2	2.43	0.83	2.23	1.27	-0.95	0.43
TG	4	5.97	3.36	8.02	3.09	1.32	2.54
TG	6	8.62	6.86	5.72	4.20	1.79	4.27
TG	8	9.86	8.59	9.77	5.37	1.72	5.74
CHOL	2	2.19	3.59	3.43	3.43	2.38	3.98
CHOL	4	7.62	7.42	8.56	7.10	6.85	6.91
CHOL	6	9.72	11.35	9.52	9.53	9.90	10.56
CHOL	8	13.40	14.56	13.27	11.72	12.14	13.67
LDL_CHOL	2	2.25	4.93	4.81	2.93	2.67	4.57
LDL_CHOL	4	9.35	9.76	8.83	5.99	6.47	7.86
LDL_CHOL	6	11.31	13.66	12.62	9.02	9.49	12.04
LDL_CHOL	8	15.92	17.86	14.92	11.88	13.14	15.64
HDL_CHOL	2	0.70	3.00	2.36	3.32	7.72	4.26
HDL_CHOL	4	0.33	5.66	1.83	7.73	8.55	6.79
HDL_CHOL	6	1.09	8.99	7.05	8.11	13.23	9.68
HDL_CHOL	8	4.00	11.13	7.54	12.18	16.68	12.32
ApoA1	2	3.31	3.47	2.84	2.28	3.06	3.29
ApoA1	4	6.52	6.61	7.40	5.65	5.97	5.88
ApoA1	6	10.39	10.43	8.22	7.69	9.56	8.78
ApoA1	8	13.58	13.64	12.68	10.72	11.24	11.34
ApoA2	2	2.97	5.04	4.26	3.08	4.88	3.79
ApoA2	4	7.32	8.84	7.23	6.95	8.29	7.21
ApoA2	6	9.03	13.49	11.19	8.71	14.03	10.58
ApoA2	8	14.59	17.64	14.85	13.93	16.74	13.83
ApoB100	2	4.54	4.57	4.15	2.12	-2.70	2.37
ApoB100	4	12.53	8.95	11.12	6.01	2.01	5.18
ApoB100	6	16.26	12.51	10.12	7.49	2.94	8.39
ApoB100	8	20.33	16.40	16.04	11.12	3.84	11.21
LDL_CHOL_HDL_CHOL	2	2.43	2.86	3.46	-0.26	-2.90	1.55
LDL_CHOL_HDL_CHOL	4	9.26	5.56	7.80	-1.84	-0.38	2.76
LDL_CHOL_HDL_CHOL	6	11.86	7.34	7.83	1.54	-1.73	4.70
LDL_CHOL_HDL_CHOL	8	13.82	10.82	9.96	0.67	-0.52	6.71
Apo.B100_ApoA1	2	1.07	2.15	2.38	0.65	-3.93	0.04
Apo.B100_ApoA1	4	6.63	3.75	5.23	2.73	-2.39	0.34
Apo.B100_ApoA1	6	7.31	4.05	4.96	2.95	-4.93	1.17
Apo.B100_ApoA1	8	9.04	5.57	6.22	4.93	-4.87	1.73
Total_Particles_ApoB	2	4.53	4.56	4.15	2.12	-2.70	2.37
Total_Particles_ApoB	4	12.53	8.95	11.12	6.01	2.01	5.19
Total_Particles_ApoB	6	16.26	12.51	10.12	7.49	2.94	8.39
Total_Particles_ApoB	8	20.33	16.40	16.04	11.12	3.84	11.21
VLDL_Particles	2	1.35	0.41	0.16	-1.01	-3.07	-2.17
VLDL_Particles	4	3.17	2.36	2.25	0.72	-2.93	-2.22
VLDL_Particles	6	2.48	2.34	1.24	-2.36	-3.81	-2.90
VLDL_Particles	8	3.36	4.09	3.78	0.07	-4.94	-4.34
IDL_Particles	2	3.78	4.79	5.52	4.93	-1.86	4.09
IDL_Particles	4	11.36	10.85	13.85	7.67	3.42	7.46

IDL_Particles	6	14.23	16.68	13.44	10.21	5.70	11.70
IDL_Particles	8	17.26	21.75	19.77	11.45	5.59	14.79
LDL_Particles	2	3.57	4.75	4.86	1.88	-1.17	3.12
LDL_Particles	4	12.33	9.47	10.55	5.41	3.96	6.17
LDL_Particles	6	14.63	12.95	11.51	7.13	5.82	9.66
LDL_Particles	8	19.56	16.82	16.16	10.80	7.99	12.73
LDL_1_Particles	2	-3.16	-1.69	-5.71	3.54	-1.85	0.89
LDL_1_Particles	4	-3.05	-2.10	0.75	9.10	-0.19	1.03
LDL_1_Particles	6	-1.20	-0.46	-5.33	10.77	-0.51	0.85
LDL_1_Particles	8	-2.74	-0.24	-2.64	10.46	-3.22	2.44
LDL_2_Particles	2	-4.57	-0.75	-3.98	5.71	1.22	3.92
LDL_2_Particles	4	-5.82	2.60	-2.36	8.05	-3.54	3.26
LDL_2_Particles	6	1.24	4.59	1.91	15.23	-3.08	8.86
LDL_2_Particles	8	-1.56	-12.50	-2.72	13.29	-2.23	13.06
LDL_3_Particles	2	-6.00	2.60	1.87	5.00	4.47	4.94
LDL_3_Particles	4	-4.97	4.16	4.11	-0.27	3.67	5.88
LDL_3_Particles	6	-9.05	7.33	11.23	8.08	6.43	9.32
LDL_3_Particles	8	-4.93	10.36	7.79	4.04	9.11	12.74
LDL_4_Particles	2	2.39	6.23	9.49	2.73	2.60	3.90
LDL_4_Particles	4	8.67	9.68	11.14	-0.78	3.42	5.38
LDL_4_Particles	6	3.45	12.81	19.19	3.74	3.90	7.69
LDL_4_Particles	8	13.25	16.90	18.21	4.42	8.18	9.57
LDL_5_Particles	2	11.39	10.80	16.15	2.16	-5.56	3.38
LDL_5_Particles	4	27.16	19.19	24.52	7.73	5.01	10.02
LDL_5_Particles	6	31.56	23.65	26.88	9.70	7.37	15.07
LDL_5_Particles	8	38.73	30.29	36.12	17.86	12.03	18.64
LDL_6_Particles	2	11.64	8.37	13.39	2.57	-5.32	3.57
LDL_6_Particles	4	27.95	17.29	26.43	15.41	11.93	11.39
LDL_6_Particles	6	34.84	23.40	19.82	15.29	17.47	17.35
LDL_6_Particles	8	41.62	28.98	38.76	27.01	19.25	22.31
TG_VLDL	2	4.43	3.62	5.22	0.15	0.97	1.16
TG_VLDL	4	10.09	7.46	11.77	3.93	5.08	6.17
TG_VLDL	6	12.06	12.48	11.54	4.29	8.33	8.38
TG_VLDL	8	15.40	16.09	16.33	8.16	10.27	11.38
TG_IDL	2	1.79	0.65	2.48	1.91	2.41	1.85
TG_IDL	4	6.07	5.09	9.79	3.32	6.30	6.70
TG_IDL	6	11.35	10.40	8.05	6.77	10.14	10.16
TG_IDL	8	12.15	12.61	12.36	8.00	13.11	13.11
TG_LDL	2	5.60	3.51	3.34	0.41	-9.54	-1.11
TG_LDL	4	12.67	6.83	12.90	1.50	-8.43	-0.94
TG_LDL	6	18.04	10.04	8.40	2.11	-11.87	-0.47
TG_LDL	8	20.48	13.27	15.91	2.99	-17.06	-0.53
TG_HDL	2	4.44	4.06	1.23	1.33	-7.95	-2.71
TG_HDL	4	7.46	4.83	10.57	1.58	-10.22	-5.13
TG_HDL	6	13.99	6.25	4.33	2.10	-13.61	-7.92
TG_HDL	8	13.40	8.72	12.04	4.09	-19.38	-11.74
CHOL_VLDL	2	5.26	0.79	2.04	1.64	-8.81	-0.76
CHOL_VLDL	4	12.14	3.98	13.91	4.74	-2.15	0.03
CHOL_VLDL	6	16.46	7.17	4.89	4.32	-3.56	2.57
CHOL_VLDL	8	17.86	9.63	13.89	4.15	-6.95	3.38
CHOL_IDL	2	6.59	6.51	5.23	9.43	-5.06	5.03
CHOL_IDL	4	15.14	12.82	19.32	12.66	4.38	10.16
CHOL_IDL	6	21.30	19.88	14.03	17.09	5.26	15.62
CHOL_IDL	8	23.73	25.78	24.09	16.63	4.49	19.91
fCHOL_VLDL	2	4.91	1.36	1.64	0.31	-4.99	-1.33
fCHOL_VLDL	4	9.19	3.26	8.55	4.30	-2.02	-0.15
fCHOL_VLDL	6	11.44	5.28	2.97	2.10	-3.82	0.77
fCHOL_VLDL	8	13.54	7.34	9.08	4.70	-6.02	1.16

fCHOL_IDL	2	6.87	6.07	5.39	8.28	-3.43	5.18
fCHOL_IDL	4	15.23	12.93	18.45	11.81	5.24	10.34
fCHOL_IDL	6	21.46	20.00	14.15	15.54	7.03	15.85
fCHOL_IDL	8	23.58	25.91	23.83	15.36	6.64	20.05
fCHOL_LDL	2	3.74	4.42	4.17	3.06	0.23	3.52
fCHOL_LDL	4	10.46	7.96	11.03	5.12	4.76	6.76
fCHOL_LDL	6	13.05	12.26	11.50	9.08	6.66	10.47
fCHOL_LDL	8	17.77	16.46	15.48	10.04	8.27	14.16
fCHOL_HDL	2	5.34	3.16	3.84	5.71	0.79	3.42
fCHOL_HDL	4	8.57	4.54	11.93	8.67	5.09	6.10
fCHOL_HDL	6	12.89	9.50	9.13	13.88	7.15	9.59
fCHOL_HDL	8	16.63	12.22	14.99	13.45	6.76	13.01
PHOSL_VLDL	2	1.96	1.50	2.91	-0.52	-0.05	-1.30
PHOSL_VLDL	4	6.37	3.92	5.28	2.61	2.88	1.52
PHOSL_VLDL	6	4.88	5.51	5.81	1.55	4.71	1.59
PHOSL_VLDL	8	7.03	8.49	7.26	5.64	6.48	2.42
PHOSL_IDL	2	3.16	5.83	6.49	3.90	5.20	6.65
PHOSL_IDL	4	12.31	13.75	14.31	8.96	11.64	13.01
PHOSL_IDL	6	15.39	20.55	16.84	12.33	18.26	18.59
PHOSL_IDL	8	19.66	25.97	22.00	16.26	21.60	22.75
PHOSL_LDL	2	1.40	3.66	3.96	1.95	1.96	3.46
PHOSL_LDL	4	7.41	7.49	7.29	3.62	5.02	6.00
PHOSL_LDL	6	8.51	10.69	10.66	6.16	7.63	9.37
PHOSL_LDL	8	12.61	14.10	12.28	7.75	10.58	12.42
PHOSL_HDL	2	0.71	1.71	1.91	2.03	4.81	3.29
PHOSL_HDL	4	1.77	3.65	3.78	4.09	6.74	5.20
PHOSL_HDL	6	2.55	6.38	6.12	5.69	11.55	7.38
PHOSL_HDL	8	5.40	8.13	8.03	7.50	13.20	9.50
ApoA1_HDL	2	3.69	3.41	2.70	2.05	2.99	2.94
ApoA1_HDL	4	6.43	6.54	6.33	5.43	4.61	5.14
ApoA1_HDL	6	10.67	10.12	7.76	6.94	7.84	7.95
ApoA1_HDL	8	13.87	13.31	11.46	10.09	9.47	10.36
ApoA2_HDL	2	2.60	4.95	4.02	2.63	4.53	3.46
ApoA2_HDL	4	6.79	8.46	6.39	6.70	7.64	6.72
ApoA2_HDL	6	7.97	12.76	10.42	7.85	12.98	9.65
ApoA2_HDL	8	13.52	16.68	13.81	13.29	15.73	12.68
ApoB_VLDL	2	1.37	0.45	0.16	-1.01	-3.09	-2.18
ApoB_VLDL	4	3.20	2.38	2.27	0.75	-2.92	-2.20
ApoB_VLDL	6	2.52	2.39	1.23	-2.36	-3.81	-2.87
ApoB_VLDL	8	3.40	4.09	3.77	0.06	-4.91	-4.34
ApoB_IDL	2	3.80	4.76	5.54	4.89	-1.89	4.12
ApoB_IDL	4	11.39	10.85	13.86	7.65	3.40	7.46
ApoB_IDL	6	14.24	16.68	13.44	10.21	5.68	11.70
ApoB_IDL	8	17.28	21.74	19.78	11.45	5.57	14.77
ApoB_LDL	2	3.56	4.75	4.86	1.88	-1.17	3.12
ApoB_LDL	4	12.32	9.47	10.54	5.41	3.96	6.17
ApoB_LDL	6	14.63	12.95	11.51	7.13	5.82	9.66
ApoB_LDL	8	19.56	16.82	16.16	10.80	7.99	12.73
TG_VLDL_1	2	-0.43	3.15	6.90	1.08	4.67	1.76
TG_VLDL_1	4	3.93	6.26	6.51	4.71	6.30	6.29
TG_VLDL_1	6	-1.45	11.31	12.85	5.16	9.18	7.98
TG_VLDL_1	8	1.32	13.51	11.04	10.18	12.64	10.88
TG_VLDL_2	2	6.62	3.03	4.09	-0.78	-3.58	0.30
TG_VLDL_2	4	15.21	7.79	18.48	1.17	3.62	6.78
TG_VLDL_2	6	21.99	13.74	11.59	3.41	7.52	10.34
TG_VLDL_2	8	25.26	19.12	21.39	5.40	8.16	14.41
TG_VLDL_3	2	8.60	4.91	5.43	-0.43	-4.59	-0.92
TG_VLDL_3	4	15.98	9.44	21.30	1.17	2.10	4.79

TG_VLDL_3	6	23.76	15.20	14.88	2.59	6.16	8.68
TG_VLDL_3	8	26.79	21.38	26.66	3.83	6.05	12.10
TG_VLDL_4	2	5.06	3.38	3.55	0.92	1.48	-0.03
TG_VLDL_4	4	5.94	6.59	10.29	1.38	5.00	5.45
TG_VLDL_4	6	12.52	9.20	9.71	2.33	9.41	8.49
TG_VLDL_4	8	12.50	14.64	16.94	3.45	11.36	10.99
TG_VLDL_5	2	-2.29	-3.57	-3.76	4.57	0.69	-0.76
TG_VLDL_5	4	-12.29	-7.15	-7.67	5.63	0.62	-1.32
TG_VLDL_5	6	-15.78	-11.74	-10.90	8.47	-1.93	-2.48
TG_VLDL_5	8	-22.11	-15.78	-14.07	7.15	-2.33	-3.35
CHOL_VLDL_1	2	1.71	-0.11	1.51	1.54	-6.34	-0.48
CHOL_VLDL_1	4	7.50	1.68	7.99	7.22	-4.46	-1.95
CHOL_VLDL_1	6	4.69	4.30	2.14	4.84	-9.14	-1.81
CHOL_VLDL_1	8	7.70	4.36	6.94	7.66	-14.17	-2.57
CHOL_VLDL_2	2	10.92	3.35	3.06	0.11	-17.66	-2.37
CHOL_VLDL_2	4	23.36	9.00	22.23	6.47	-5.71	-0.35
CHOL_VLDL_2	6	30.99	14.36	7.57	5.41	-8.58	2.42
CHOL_VLDL_2	8	34.86	18.93	23.09	7.70	-14.80	3.71
CHOL_VLDL_3	2	11.85	8.43	6.93	1.06	-15.24	-1.21
CHOL_VLDL_3	4	24.91	14.55	29.08	5.99	-3.29	3.41
CHOL_VLDL_3	6	31.52	20.76	17.16	4.83	-0.51	8.41
CHOL_VLDL_3	8	36.07	27.58	33.46	7.42	-6.16	11.35
CHOL_VLDL_4	2	9.65	4.77	2.96	4.83	-16.39	-2.43
CHOL_VLDL_4	4	19.78	9.63	22.50	7.86	-2.50	3.49
CHOL_VLDL_4	6	30.77	14.45	7.29	9.99	-1.78	7.94
CHOL_VLDL_4	8	30.67	20.62	23.52	11.03	-8.03	10.51
CHOL_VLDL_5	2	-16.54	-8.51	-21.06	-19.87	-4.90	-22.01
CHOL_VLDL_5	4	-45.45	-14.57	-40.26	27.16	-11.22	-21.50
CHOL_VLDL_5	6	-39.55	-34.83	-59.94	10.80	-35.90	-21.84
CHOL_VLDL_5	8	-69.42	-52.31	-84.30	19.66	-34.46	-25.66
fCHOL_VLDL_1	2	1.02	1.07	5.66	0.11	4.57	3.60
fCHOL_VLDL_1	4	10.13	8.04	7.85	6.00	8.84	8.74
fCHOL_VLDL_1	6	10.10	13.21	11.12	6.71	13.30	11.80
fCHOL_VLDL_1	8	11.72	16.50	12.26	11.95	17.20	14.52
fCHOL_VLDL_2	2	6.81	2.67	2.78	-1.35	2.82	-0.48
fCHOL_VLDL_2	4	14.91	7.53	16.58	3.99	6.16	-1.41
fCHOL_VLDL_2	6	17.63	11.29	9.00	-0.41	4.27	-19.67
fCHOL_VLDL_2	8	21.81	13.97	19.24	1.40	0.99	0.28
fCHOL_VLDL_3	2	9.40	6.79	5.21	1.06	-9.55	-0.75
fCHOL_VLDL_3	4	19.11	12.30	21.15	5.98	-1.73	3.09
fCHOL_VLDL_3	6	22.67	17.58	13.88	3.29	0.35	6.65
fCHOL_VLDL_3	8	27.34	23.59	26.85	6.65	-3.23	8.64
fCHOL_VLDL_4	2	9.43	5.79	3.71	6.15	-8.39	3.22
fCHOL_VLDL_4	4	16.65	11.10	20.47	11.21	2.72	7.91
fCHOL_VLDL_4	6	23.26	16.96	11.02	10.28	4.34	12.80
fCHOL_VLDL_4	8	25.57	23.17	23.70	10.27	0.73	15.54
PHOSL_VLDL_1	2	-2.42	1.35	5.44	0.84	3.30	1.35
PHOSL_VLDL_1	4	3.96	4.96	5.93	3.33	6.16	5.94
PHOSL_VLDL_1	6	-1.46	9.31	11.40	4.63	10.17	7.39
PHOSL_VLDL_1	8	-0.06	11.77	8.76	8.53	13.61	10.27
PHOSL_VLDL_2	2	5.92	3.76	4.81	-1.01	-4.69	-0.69
PHOSL_VLDL_2	4	16.41	9.61	18.23	2.14	2.54	5.14
PHOSL_VLDL_2	6	21.26	14.42	12.38	3.20	7.08	8.23
PHOSL_VLDL_2	8	24.78	20.15	22.18	6.96	7.39	11.24
PHOSL_VLDL_3	2	6.95	6.54	6.65	-0.28	-2.08	0.68
PHOSL_VLDL_3	4	16.58	13.67	19.43	3.48	4.75	6.93
PHOSL_VLDL_3	6	20.98	18.68	17.80	3.19	10.48	10.89
PHOSL_VLDL_3	8	24.69	25.66	27.20	7.25	11.98	13.87

PHOSL_VLDL_4	2	5.31	2.31	3.12	1.78	-3.30	-0.04
PHOSL_VLDL_4	4	9.07	6.08	12.19	2.45	2.55	3.81
PHOSL_VLDL_4	6	15.19	8.30	7.82	3.26	4.63	6.52
PHOSL_VLDL_4	8	15.01	12.84	15.94	2.68	4.86	7.77
PHOSL_VLDL_5	2	-1.95	-5.82	-8.80	7.53	-6.29	-6.46
PHOSL_VLDL_5	4	-12.38	-11.32	-9.46	7.77	-5.05	-10.61
PHOSL_VLDL_5	6	-11.24	-22.43	-22.35	14.28	-16.21	-15.97
PHOSL_VLDL_5	8	-24.45	-30.80	-24.28	11.30	-18.78	-22.47
TG_LDL_1	2	-0.23	-3.98	-8.35	1.18	-9.81	-3.84
TG_LDL_1	4	-0.50	-4.69	-0.18	1.62	-12.91	-7.27
TG_LDL_1	6	5.35	-4.54	-11.63	2.21	-20.63	-10.65
TG_LDL_1	8	2.98	-6.01	-6.15	0.40	-31.90	-13.12
TG_LDL_2	2	-4.37	-2.05	8.03	-0.56	-0.84	16.98
TG_LDL_2	4	-6.31	-1.86	9.93	-2.30	-5.16	14.15
TG_LDL_2	6	-5.82	-1.11	11.56	-1.40	-6.83	12.12
TG_LDL_2	8	-8.13	-1.02	9.29	-5.35	-8.49	10.80
TG_LDL_3	2	-1.38	-1.10	-0.09	1.50	-4.35	-1.28
TG_LDL_3	4	0.64	-1.42	0.85	0.94	-4.55	-3.13
TG_LDL_3	6	-0.66	-2.55	-0.43	2.37	-7.32	-4.81
TG_LDL_3	8	-0.54	-3.76	-1.06	2.03	-9.71	-6.16
TG_LDL_4	2	7.59	5.25	8.75	-1.60	-12.43	-3.70
TG_LDL_4	4	14.63	7.09	24.78	-4.32	-16.06	-6.54
TG_LDL_4	6	16.08	8.60	24.45	-4.05	-24.84	-10.12
TG_LDL_4	8	21.07	11.32	26.61	-4.33	-29.21	-14.22
TG_LDL_5	2	11.85	8.43	13.43	0.09	-16.71	-1.78
TG_LDL_5	4	27.01	15.31	28.04	4.66	-4.61	2.79
TG_LDL_5	6	31.87	18.88	21.46	5.30	-8.88	3.70
TG_LDL_5	8	37.28	23.80	34.78	9.86	-11.02	3.35
TG_LDL_6	2	5.32	4.60	9.91	2.67	-2.96	2.24
TG_LDL_6	4	16.13	11.09	16.17	6.76	7.42	7.18
TG_LDL_6	6	17.22	15.65	14.04	6.48	11.70	11.12
TG_LDL_6	8	23.10	18.38	25.28	12.41	13.12	14.65
CHOL_LDL_1	2	-5.09	-1.56	-6.11	4.84	0.92	2.22
CHOL_LDL_1	4	-5.75	-1.34	-0.63	11.33	2.99	2.53
CHOL_LDL_1	6	-4.29	0.47	-5.16	13.90	3.40	3.09
CHOL_LDL_1	8	-6.76	1.19	-3.79	13.13	2.26	5.04
CHOL_LDL_2	2	-7.60	-0.32	-4.24	9.10	5.63	5.30
CHOL_LDL_2	4	-11.58	-16.46	-4.88	12.51	0.80	5.14
CHOL_LDL_2	6	-5.37	-12.74	2.58	22.12	3.22	11.54
CHOL_LDL_2	8	-9.12	-12.31	-4.92	19.07	6.35	-2.21
CHOL_LDL_3	2	-8.95	2.81	-10.28	7.13	7.36	6.15
CHOL_LDL_3	4	-10.11	4.45	-9.17	0.41	6.60	7.53
CHOL_LDL_3	6	-16.05	8.05	12.17	11.02	10.46	11.60
CHOL_LDL_3	8	-12.19	10.99	7.46	5.30	14.30	15.49
CHOL_LDL_4	2	-0.27	6.05	9.67	3.22	6.66	4.91
CHOL_LDL_4	4	4.36	9.21	7.74	-1.63	5.60	6.05
CHOL_LDL_4	6	-2.83	11.90	19.72	3.25	6.92	8.82
CHOL_LDL_4	8	6.72	15.57	15.73	3.29	13.01	10.64
CHOL_LDL_5	2	10.31	10.69	16.78	2.48	-2.49	4.42
CHOL_LDL_5	4	26.20	19.31	23.46	7.95	7.50	10.76
CHOL_LDL_5	6	29.81	23.53	28.11	10.27	10.26	16.31
CHOL_LDL_5	8	37.53	30.28	35.87	19.07	16.70	19.97
CHOL_LDL_6	2	11.57	9.30	15.34	2.96	-2.18	4.24
CHOL_LDL_6	4	28.20	18.26	25.99	15.63	14.73	13.19
CHOL_LDL_6	6	34.16	24.95	23.38	16.26	21.43	19.21
CHOL_LDL_6	8	41.51	30.70	32.38	28.42	25.21	24.48
fCHOL_LDL_1	2	-0.07	0.36	-3.65	6.56	-1.01	3.09
fCHOL_LDL_1	4	3.71	2.36	6.99	14.08	5.15	6.16

fCHOL_LDL_1	6	9.76	6.80	0.29	20.33	7.07	9.07
fCHOL_LDL_1	8	9.55	10.07	6.18	19.70	5.60	13.01
fCHOL_LDL_2	2	0.20	2.52	-1.26	9.77	2.73	4.33
fCHOL_LDL_2	4	1.80	7.20	3.70	16.72	0.74	6.60
fCHOL_LDL_2	6	11.28	12.76	6.63	25.72	3.06	13.17
fCHOL_LDL_2	8	11.72	0.15	6.14	25.49	4.21	19.05
fCHOL_LDL_3	2	0.49	3.98	2.28	3.67	4.59	4.78
fCHOL_LDL_3	4	0.70	5.33	6.62	2.53	4.19	6.62
fCHOL_LDL_3	6	1.93	9.75	10.61	8.80	6.32	9.83
fCHOL_LDL_3	8	6.48	13.07	10.91	4.95	8.10	13.55
fCHOL_LDL_4	2	3.69	6.83	8.93	3.74	4.28	4.98
fCHOL_LDL_4	4	7.60	10.18	10.77	2.15	4.45	7.33
fCHOL_LDL_4	6	6.30	14.09	18.15	6.01	5.88	10.76
fCHOL_LDL_4	8	14.54	18.33	18.36	7.44	9.73	13.60
fCHOL_LDL_5	2	11.71	10.18	14.08	3.22	-4.72	3.53
fCHOL_LDL_5	4	25.66	17.25	22.92	8.25	4.48	9.40
fCHOL_LDL_5	6	30.88	22.25	24.28	11.33	6.49	14.71
fCHOL_LDL_5	8	38.30	28.99	33.86	19.19	9.79	18.62
fCHOL_LDL_6	2	13.93	9.85	13.27	4.39	-6.61	4.33
fCHOL_LDL_6	4	30.55	18.38	32.63	15.70	12.11	14.83
fCHOL_LDL_6	6	41.72	27.11	23.60	21.54	19.69	22.45
fCHOL_LDL_6	8	46.89	34.47	43.32	31.18	20.73	29.62
PHOSL_LDL_1	2	-4.93	-2.55	-6.28	3.58	0.24	1.30
PHOSL_LDL_1	4	-6.52	-3.36	-1.62	7.96	1.11	0.86
PHOSL_LDL_1	6	-5.48	-2.33	-6.15	10.32	1.13	0.67
PHOSL_LDL_1	8	-8.40	-2.53	-5.48	9.17	-0.52	1.91
PHOSL_LDL_2	2	-8.29	-2.05	-3.97	5.82	4.63	4.62
PHOSL_LDL_2	4	-13.11	0.47	-5.03	6.09	-0.08	4.19
PHOSL_LDL_2	6	-9.00	1.95	1.33	13.63	1.68	9.30
PHOSL_LDL_2	8	-13.79	-17.51	-5.72	8.67	3.97	13.45
PHOSL_LDL_3	2	-7.56	2.15	1.76	5.57	5.82	5.17
PHOSL_LDL_3	4	-8.55	3.27	3.22	-0.99	4.67	5.97
PHOSL_LDL_3	6	-13.40	6.27	10.88	8.07	7.82	9.46
PHOSL_LDL_3	8	-10.44	8.76	6.78	2.57	11.03	12.85
PHOSL_LDL_4	2	0.02	5.33	9.22	2.55	5.48	4.49
PHOSL_LDL_4	4	3.67	8.05	7.35	-2.56	4.75	5.25
PHOSL_LDL_4	6	-3.06	10.44	18.48	1.86	5.88	7.42
PHOSL_LDL_4	8	5.75	13.65	14.77	1.27	11.30	9.09
PHOSL_LDL_5	2	9.97	9.88	15.64	1.74	-3.27	3.47
PHOSL_LDL_5	4	24.88	17.87	22.18	6.15	5.78	9.35
PHOSL_LDL_5	6	28.62	21.80	26.53	8.55	7.99	14.28
PHOSL_LDL_5	8	35.82	28.21	33.73	16.15	13.77	17.48
PHOSL_LDL_6	2	10.20	7.75	13.71	2.57	-1.04	3.99
PHOSL_LDL_6	4	25.45	15.99	22.21	13.31	13.71	11.93
PHOSL_LDL_6	6	30.38	22.09	21.05	14.09	20.33	17.46
PHOSL_LDL_6	8	37.45	27.40	35.31	24.67	24.03	22.21
ApoB_LDL_1	2	-3.16	-1.68	-5.71	3.54	-1.85	0.89
ApoB_LDL_1	4	-3.05	-2.10	0.74	9.06	-0.19	1.05
ApoB_LDL_1	6	-1.22	-0.46	-5.33	10.75	-0.50	0.86
ApoB_LDL_1	8	-2.75	-0.23	-2.65	10.42	-3.21	2.46
ApoB_LDL_2	2	-4.55	-0.72	-3.96	5.71	1.24	3.90
ApoB_LDL_2	4	-5.79	2.61	-2.36	8.03	-3.51	3.26
ApoB_LDL_2	6	1.28	4.62	1.93	15.21	-3.06	8.87
ApoB_LDL_2	8	-1.51	-12.48	-2.71	13.28	-2.22	13.09
ApoB_LDL_3	2	-6.01	2.60	1.88	4.97	4.46	4.94
ApoB_LDL_3	4	-4.98	4.16	4.10	-0.30	3.67	5.90
ApoB_LDL_3	6	-9.06	7.33	11.22	8.09	6.43	9.33
ApoB_LDL_3	8	-4.93	10.38	7.79	4.03	9.11	12.74

ApoB_LDL_4	2	2.38	6.23	9.49	2.75	2.60	3.90
ApoB_LDL_4	4	8.68	9.67	11.15	-0.79	3.42	5.38
ApoB_LDL_4	6	3.45	12.82	19.20	3.74	3.90	7.69
ApoB_LDL_4	8	13.25	16.90	18.21	4.42	8.19	9.55
ApoB_LDL_5	2	11.39	10.81	16.14	2.15	-5.57	3.37
ApoB_LDL_5	4	27.17	19.19	24.52	7.73	5.01	10.02
ApoB_LDL_5	6	31.57	23.66	26.86	9.70	7.36	15.06
ApoB_LDL_5	8	38.74	30.29	36.12	17.86	12.02	18.65
ApoB_LDL_6	2	11.64	8.36	13.40	2.57	-5.32	3.57
ApoB_LDL_6	4	27.95	17.27	26.43	15.42	11.94	11.39
ApoB_LDL_6	6	34.84	23.40	19.82	15.31	17.47	17.36
ApoB_LDL_6	8	41.62	28.97	38.76	27.01	19.25	22.32
TG_HDL_1	2	3.53	6.86	0.65	3.58	-6.78	-0.92
TG_HDL_1	4	5.71	8.09	11.04	6.60	-9.18	-4.77
TG_HDL_1	6	11.53	9.68	5.13	5.66	-11.85	-9.48
TG_HDL_1	8	10.39	11.94	14.05	9.37	-17.56	-15.87
TG_HDL_2	2	5.27	6.09	4.42	2.65	-7.60	-1.96
TG_HDL_2	4	7.78	7.49	12.10	2.47	-13.15	-4.93
TG_HDL_2	6	16.14	9.36	9.42	4.58	-16.02	-5.29
TG_HDL_2	8	15.41	12.75	15.39	5.88	-21.94	-10.37
TG_HDL_3	2	6.36	4.73	2.31	1.22	-9.21	-2.94
TG_HDL_3	4	10.69	7.30	11.10	2.40	-12.17	-3.77
TG_HDL_3	6	19.75	9.71	6.02	4.78	-15.42	-3.46
TG_HDL_3	8	21.02	14.49	13.85	7.80	-22.18	-5.37
TG_HDL_4	2	3.42	1.16	-1.42	-0.68	-8.20	-5.89
TG_HDL_4	4	6.81	0.94	4.13	-2.56	-10.17	-8.06
TG_HDL_4	6	13.01	0.46	-2.76	-2.50	-15.99	-11.43
TG_HDL_4	8	12.74	2.80	2.21	-0.95	-20.99	-14.61
CHOL_HDL_1	2	-1.96	3.83	2.56	8.43	9.12	7.08
CHOL_HDL_1	4	-1.54	7.45	5.44	17.71	12.40	11.72
CHOL_HDL_1	6	-2.94	12.17	9.77	20.31	18.53	14.66
CHOL_HDL_1	8	-2.11	14.32	11.57	25.90	20.75	17.68
CHOL_HDL_2	2	0.27	3.66	5.03	3.75	10.25	6.65
CHOL_HDL_2	4	-3.45	5.37	1.69	7.42	7.48	7.66
CHOL_HDL_2	6	-2.60	8.56	11.18	7.63	13.05	10.78
CHOL_HDL_2	8	-1.25	9.89	9.44	9.65	15.69	12.30
CHOL_HDL_3	2	1.25	3.12	3.46	2.59	7.94	4.92
CHOL_HDL_3	4	1.18	5.40	4.16	5.19	9.69	7.88
CHOL_HDL_3	6	1.85	9.63	9.38	7.52	16.07	11.22
CHOL_HDL_3	8	7.05	12.40	11.10	10.07	18.59	14.57
CHOL_HDL_4	2	0.08	2.05	0.46	1.72	6.74	2.09
CHOL_HDL_4	4	0.48	4.49	-0.07	3.36	9.91	5.04
CHOL_HDL_4	6	0.46	7.43	2.73	4.35	14.49	7.87
CHOL_HDL_4	8	4.32	9.77	4.07	9.35	19.75	11.21
fCHOL_HDL_1	2	5.88	6.62	6.85	9.04	3.47	6.31
fCHOL_HDL_1	4	12.18	10.80	16.30	14.70	9.96	11.21
fCHOL_HDL_1	6	15.15	17.88	16.08	19.58	14.80	15.88
fCHOL_HDL_1	8	20.11	22.31	22.24	21.19	15.32	20.08
fCHOL_HDL_2	2	6.86	6.95	7.20	8.04	3.64	6.83
fCHOL_HDL_2	4	12.56	10.69	17.04	10.50	10.39	12.00
fCHOL_HDL_2	6	15.88	18.37	16.88	16.70	17.37	17.30
fCHOL_HDL_2	8	23.12	23.27	23.66	16.27	17.36	22.04
fCHOL_HDL_3	2	9.17	7.06	6.75	6.97	-0.09	4.41
fCHOL_HDL_3	4	17.16	11.28	19.38	9.67	7.82	10.10
fCHOL_HDL_3	6	25.60	19.41	16.20	18.85	13.94	16.10
fCHOL_HDL_3	8	31.49	25.61	25.73	21.20	14.02	21.85
fCHOL_HDL_4	2	9.50	5.52	3.58	4.42	-2.92	1.70
fCHOL_HDL_4	4	17.12	8.16	17.57	6.12	5.81	7.37

fCHOL_HDL_4	6	25.86	14.75	9.97	14.14	10.39	11.89
fCHOL_HDL_4	8	32.11	20.26	22.07	17.84	9.67	17.46
PHOSL_HDL_1	2	-0.51	3.32	2.91	6.93	5.76	6.04
PHOSL_HDL_1	4	1.09	6.12	7.41	13.63	8.77	9.27
PHOSL_HDL_1	6	0.17	9.99	9.75	16.09	14.53	11.60
PHOSL_HDL_1	8	1.53	12.08	12.46	19.43	15.01	13.68
PHOSL_HDL_2	2	-0.42	2.29	4.26	2.26	6.73	4.97
PHOSL_HDL_2	4	-2.11	3.49	2.77	3.92	5.21	5.43
PHOSL_HDL_2	6	-2.57	5.79	9.78	4.64	10.72	7.60
PHOSL_HDL_2	8	-0.79	6.80	8.95	5.61	12.04	8.75
PHOSL_HDL_3	2	1.20	2.09	2.43	1.13	5.35	3.57
PHOSL_HDL_3	4	2.82	4.26	4.57	2.26	7.83	6.22
PHOSL_HDL_3	6	3.97	7.81	7.72	4.99	14.35	9.27
PHOSL_HDL_3	8	8.86	10.43	9.99	6.58	16.15	12.48
PHOSL_HDL_4	2	-0.39	0.65	0.10	0.89	4.03	1.21
PHOSL_HDL_4	4	0.86	2.43	1.07	0.55	7.68	3.55
PHOSL_HDL_4	6	1.03	4.53	1.60	2.92	12.00	5.88
PHOSL_HDL_4	8	4.23	6.44	3.41	5.01	15.58	8.74
ApoA1_HDL_1	2	5.74	5.73	4.39	6.56	1.93	5.39
ApoA1_HDL_1	4	9.46	10.51	10.78	16.49	3.43	7.98
ApoA1_HDL_1	6	14.39	14.93	11.19	17.12	6.47	10.97
ApoA1_HDL_1	8	16.19	18.69	15.92	21.58	6.05	12.02
ApoA1_HDL_2	2	-0.08	2.56	3.29	2.12	4.70	3.42
ApoA1_HDL_2	4	1.37	4.96	3.67	3.68	5.99	4.47
ApoA1_HDL_2	6	0.50	7.50	8.41	4.78	10.84	6.38
ApoA1_HDL_2	8	3.94	9.30	9.71	7.34	12.53	7.94
ApoA1_HDL_3	2	1.23	2.24	2.44	1.52	4.76	3.32
ApoA1_HDL_3	4	1.51	3.75	4.38	3.23	5.98	4.89
ApoA1_HDL_3	6	2.47	7.12	7.24	4.97	10.50	7.51
ApoA1_HDL_3	8	6.72	9.23	9.50	6.48	11.91	10.15
ApoA1_HDL_4	2	3.02	2.50	1.22	0.47	2.45	1.48
ApoA1_HDL_4	4	6.12	4.98	4.06	1.71	5.19	3.70
ApoA1_HDL_4	6	9.71	8.03	4.19	2.33	7.49	6.12
ApoA1_HDL_4	8	13.54	10.78	7.98	5.33	10.44	8.79
ApoA2_HDL_1	2	1.75	7.29	6.99	7.49	3.87	6.52
ApoA2_HDL_1	4	8.21	12.59	12.96	14.60	8.69	9.97
ApoA2_HDL_1	6	5.39	17.56	16.98	16.62	15.87	12.86
ApoA2_HDL_1	8	11.33	21.58	20.85	22.88	16.14	14.97
ApoA2_HDL_2	2	0.55	5.87	7.01	4.22	7.55	6.66
ApoA2_HDL_2	4	4.94	10.57	9.06	9.40	10.40	10.28
ApoA2_HDL_2	6	1.88	15.85	17.30	12.15	18.81	13.95
ApoA2_HDL_2	8	8.75	20.18	18.74	17.41	20.87	17.17
ApoA2_HDL_3	2	1.96	5.07	5.41	3.06	5.81	4.68
ApoA2_HDL_3	4	5.78	8.57	8.18	5.91	8.37	8.36
ApoA2_HDL_3	6	6.15	13.80	13.79	8.93	15.39	11.89
ApoA2_HDL_3	8	12.45	18.06	15.99	13.18	17.53	15.44
ApoA2_HDL_4	2	1.87	3.81	1.84	2.21	4.30	2.15
ApoA2_HDL_4	4	5.69	7.02	4.30	4.76	9.37	5.61
ApoA2_HDL_4	6	6.81	10.84	5.84	5.89	14.22	8.69
ApoA2_HDL_4	8	12.41	14.62	10.22	11.98	18.20	12.30
